# JAK inhibitor withdrawal causes a transient pro-inflammatory cascade: A potential mechanism for major adverse cardiac events

**DOI:** 10.1371/journal.pone.0311706

**Published:** 2025-06-16

**Authors:** Ilya Gurevic, Loic Meudec, Xavier Mariette, Gaetane Nocturne, Sara S. McCoy

**Affiliations:** 1 Department of Medicine, School of Medicine and Public Health, University of Wisconsin, Madison, Wisconsin, United States of America; 2 Center for Immunology of Viral Infections and Autoimmune Diseases, INSERM UMR, Université Paris-Saclay, Le Kremlin-Bicêtre, Paris, France; 3 Université Paris-Saclay, Center for Immunology of Viral Infections and Auto-immune Diseases (IMVA), Institut pour la Santé et la Recherche Médicale (INSERM) UMR, Le Kremlin-Bicêtre, Paris, France; 4 Assistance Publique – Hôpitaux de Paris, Hôpital Bicêtre, Department of Rheumatology, Le Kremlin Bicêtre, France.; University of Illinois, UNITED STATES OF AMERICA

## Abstract

**Objective:**

Our objective was to define the effect of JAK inhibitor (JAKinib) withdrawal on JAK/STAT biochemical response in the context of systemic rheumatic diseases.

**Methods:**

We tested Type I (bind kinase active conformation) and Type II (bind kinase inactive conformation) JAKinibs in vitro using mesenchymal stromal cells (MSCs) and human umbilical vein endothelial cells (HUVECs). We translated our findings in vivo studying NK cells from rheumatoid arthritis (RA) patients treated with Type I JAKinibs or methotrexate.

**Results:**

Type I JAKinibs (ruxolitinib and baricitinib) increased phosphoJAK1 (pJAK1) and pJAK2 of IFNγ-stimulated MSCs and HUVECs in a time- and dose- dependent manner, with effect peaking after 24 hours. As expected, pSTAT1 was completely suppressed by JAKinibs. We found a marked and rapid *increase* of pSTATs upon discontinuation of Type I JAKinibs, that occurred to a lesser extent after Type II JAKinib withdrawal. Type I JAKinib withdrawal increased interferon and urokinase expression when compared to Type II JAKinib withdrawal. We found NK cells from RA patients taking Type I JAKinibs had a pro-inflammatory profile after JAKinib withdrawal compared to patients on methotrexate.

**Conclusions:**

Type I JAKinibs paradoxically accumulate functionally defective pJAK. Upon withdrawal, the primed pJAKs are de-repressed and initiate a pSTAT signaling cascade, resulting in high interferon and urokinase. Type II JAKinibs do not cause pJAK accumulation, pSTAT cascade, and subsequent pro-inflammatory transcripts. The resultant cytokines and proteins produced from this cascade might explain adverse cardiac outcomes. Thus, JAKinib withdrawal is a possible mechanism contributing to the major adverse cardiac events described with JAKinib therapy.

## Introduction

Janus kinase inhibitors (JAKinibs) are increasingly used to treat autoimmune diseases due to their efficacy in conditions such as rheumatoid arthritis (RA), systemic lupus erythematosus (SLE), and psoriatic arthritis, among others. Alongside this increased use of JAKinibs, there has been a parallel rise in concerns about the adverse effects associated with this class of medications. Phase 2 trials initially identified possible risks of JAKinibs compared to placebo that included major adverse cardiac events (MACE), malignancy, venous thromboembolism, infections, and worsened lipid profile [1,2].

One of these concerns, MACE, includes death from cardiovascular causes, nonfatal myocardial infarction, or nonfatal stroke. A patient’s risk of MACE may increase based on the presence of atherosclerosis, a condition where plaque builds up in the walls of the arteries. Patients with autoimmune diseases are already at an increased risk for atherosclerosis due to factors such as advanced age, obesity, diabetes, smoking history, and aberrant lipid profile [3,4]. Therefore, patients with the greatest risk of MACE among JAKinib users are ≥ 65 years old, with atherosclerosis risk factors [5,6]. One study found that patients who used JAKinibs had a 33% increased risk of MACE, which resulted in 3.4% of patients experiencing a MACE compared to 2.5% who used tumor necrosis factor (TNF) inhibitors [7]. In contrast, another study found that patients who used JAKinibs to treat RA and psoriatic arthritis had similar rates of mortality to those who received either TNF inhibitors or methotrexate [8]. They found that MACE occurred at s rate of <0.5 events per 100 patient years, across all comparisons including JAKinib, TNF inhibitor, and methotrexate treated groups [8]. Despite this contrasting evidence in MACE-related adverse effects, the Food and Drug Administration (FDA) issued a black box warning for serious cardiac events including heart attack, stroke, blood clots, and death with JAKinibs used to treat inflammatory diseases [9] and the European Medicines Agency (EMA) safety committee recommended restricting JAKinib use [10]. Several hypotheses exist for why MACE might occur with JAKinib use.

Researchers hypothesize that JAKinibs worsen dyslipidemia, thereby increasing the risk of MACE [11]. While others hypothesize that specific JAKinib targets block individual signaling pathways that counterintuitively alter pro- and anti-thrombotic signaling cascades [12]. For example, the inhibition of JAK2 can drive thrombocytosis [13], which might contribute to the risk of MACE. More mechanistic insight is needed to understand how JAKinib use influences the risk of MACE to help prevent occurrences in at-risk patients.

**Fig 1:**
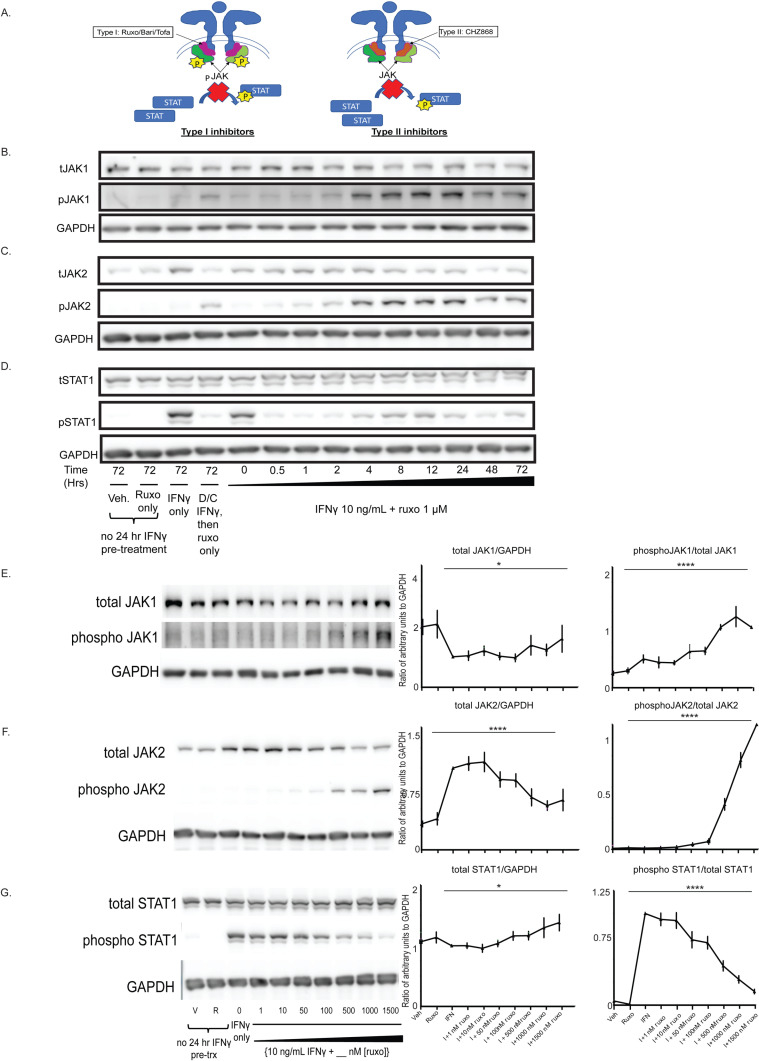
The Type I JAKinib, ruxolitinib, promotes pJAK1 and pJAK2 phosphorylation in MSCs from salivary glands. (A) Representative figure showing differences between Type I and Type II JAKinibs. Type I JAKinibs are ATP-competitive inhibitors that stabilize a kinase-active conformation of JAK. Because the JAK is stabilized in the kinase active conformation, it accumulates phosphylation. Type II inhibitors are also ATP-competitive binders but stabilize the inactive kinase conformation of JAK and do not accumulate phosphorylation on JAK. Both Type I and II JAKinibs block kinase function and inhibit downstream STAT phosphorylation. B-G were performed using MSCs from a control subject. We treated MSCs with vehicle, ruxolitinib (1 µM), IFNγ only (10 ng/mL), IFNγ then switching to ruxolitinib only, or IFNγ 10 ng/mL in combination with ruxolitnib 1 uM for varying periods of time or for 48 hours at varying doses of ruxolitinib (0-1500nM). At each time or dose of ruxolitinib, we harvested cells for protein and performed western blot for the indicated target. **(B)** Total JAK1 was stable regardless of treatment time with ruxolitinib, but the proportion of pJAK1 increased starting at 4 hours and peaked after 24 hours of treatment; (C) the proportion of pJAK2 increased with ruxolitinib treatment, starting at 4 hours and peaked after 24 hours; (D) pSTAT1 was suppressed almost immediately after introduction of ruxolitinib. (E-G) were performed with replicates (n = 6; 3 SjD and 3 control) of our treatment conditions on the right with a representative western on the left; (E) pJAK1 proportion increased in a dose-dependent manner with increasing ruxolitnib; (F) pJAK2 proportion increased as ruxolitinib dose increased; (G) pSTAT1 decreased in a dose response relationship with increasing ruxolitinib. Error bars represent standard deviation; *p = 0.05; p = **** < 0.0001 represent the ANOVA-derived significance for the difference between all values. I = IFN.

JAKinibs bind the adenosine triphosphate (ATP)-binding site of JAK enzymes, which prevents the JAKs from carrying out their enzymatic activity, transferring phosphate from ATP to downstream Signal Transducer and Activator of Transcription (STAT) proteins [14]. Generally grouped into two main classifications (Type I or Type II inhibitors), Type I inhibitors interact with and stabilize the active conformation of JAK, leading to the abrogation of signaling through the JAK-STAT pathway (Fig 1A). However, while Type I inhibitors prevent JAK enzymes from signaling through JAK-STAT pathways, the ubiquitination and degradation of JAK are blocked. This leads to the paradoxical accumulation of phosphorylation of the JAK [15]. The accumulation of phosphorylation on the JAK results in a rapid STAT activation upon Type I JAKinib withdrawal leading to a cytokine-rebound syndrome (hereafter, cytokine rebound). In contrast, Type II inhibitors stabilize the inactive kinase conformation of JAK, are not associated with phosphorylation when bound [16], and do not cause JAK hyperphosphorylation or downstream withdrawal signaling [15]. However, no Type II JAKinibs are currently available for clinical use due to their pharmacokinetic properties [17].

Cytokine rebounds occur with Type I JAKinibs in other disease systems. Researchers found that Type I JAKinibs cause cytokine rebounds in cell lines that model myelofibrosis [15,18–20], resulting in pathogenic signaling upon drug withdrawal, particularly in JAK2 V617F myelofibrosis patients [15] who have constitutively active JAK-STAT signaling and resultant increased inflammatory cytokines [21]. Although there is evidence of this withdrawal phenomenon, the relevance of these findings to JAKinibs in the context of rheumatic disease is unclear. There are instances of patients with rheumatic diseases experiencing withdrawal symptoms after they have abruptly stopped use of Type I JAKinibs (Type I inhibitors used for treatment; Table 1 [22–26]). Even short interruptions of JAKinib use (e.g., baricitinib or tofacitinib), worsen RA symptoms in trials [27,28] or autoinflammatory syndromes [29]. However, if patients taper their use of JAKinibs [30,31], flare rates are reduced [28]. Additionally, there was one case of a life-threatening cytokine rebound reported in an RA patient after JAKinib withdrawal [32]. No head-to-head studies have compared withdrawal phenomenon with different kinds of JAKinibs in rheumatic disease. The role of cytokine rebound upon JAKinib withdrawal in MACE and rheumatologic diseases is unknown.

**Table 1:** JAK inhibitor types, their targets, and their IC_50._

		JAK1	JAK2	JAK3	TYK2
Type I	Abrocitinib	29	803	>10,000	1253
	Baricitinib	5.9	5.7	560	53
	Fedratinib	15	3	3	48
	Delgocitinib	2.8	2.6	13	58
	Filgotinib	10	28	810	116
	Peficitinib	3.9	5	0.7	4.8
	Ruxolitinib	3.3	2.8	428	19
	Tofacitinib	3.2	4.1	1	34
	Upadacitinib	47	120	2300	4700
Other	Deucravacitinib^¶^	>10000	>10000	>10000	0.20
	Ritlecitinib^*^	>10000	>10000	33.1	>10000

Table 1 shows JAK1/2/3/Tyk2 inhibitors by mechanism (Type I or other), anticipated target (JAK1/2/3/Tyk2) denoted by shading of the cell, and the IC50 (given numerically);

¶ Allosteric inhibitor;

* irreversible inhibitor. From Szilveszter et al. [23] and Medchemexpress.

In this study, our objective was to understand the downstream effects of JAKinib withdrawal in rheumatic diseases and identify mechanisms that might explain the increased risk of MACE reported with JAKinib use. We used mesenchymal stromal cells (MSCs) and human umbilical vein endothelial cells (HUVECs) to show that cells implicated in vascular health demonstrate similar JAK-STAT activation upon Type I JAKinib withdrawal akin to what was seen in past myelofibrosis cell models. This led us to ask what downstream effects the resulting STAT signaling cascade had on the transcriptome. Next, we translated our findings *in vivo* to determine the real-life effects of JAKinib withdrawal on inflammatory cells, especially natural killer (NK) cells in patients with RA treated with JAKinibs. Our study aims to enhance understanding of a potential novel mechanism associated with the withdrawal of certain JAKinibs, which may contribute to MACE.

## Methods

### Patients

All the work described was performed and approved under the University of Wisconsin Health Sciences IRB 2018−0815 and Comité d’éthique de la recherche, CER Paris-Saclay 2020-A00509-30. Written consent was obtained from all participants. Samples were collected from 01/2021–08/2021. Mesenchymal stromal cells (MSCs, fibroblasts) were generated from salivary gland tissue and express CD90 with a transcriptional fingerprint comprising *Pdgfra* and *Col15a1* [33]. These cells are adherent to plastic and display the expected morphology *in vitro* [34,35]. Glands were selected as an MSC/fibroblast source because they are harvested through a low-risk outpatient procedure and MSC isolation from this tissue is a well-established protocol in our hands [34]. Glands were derived from Sjögren disease (met 2016 ACR/EULAR criteria [36]) (n = 3) as an autoimmune disease with high cardiovascular disease prevalence compared to healthy controls [37]. Control participants had dry symptoms but no diagnosed autoimmune disease (n = 6). All MSCs were derived from female participants (n = 9) with an average overall age of 41 years; an average age of 40 years for Sjögren disease subjects and 45 years for the control subjects. Blood samples were collected from RA patients referred to the Department of Rheumatology of Bicêtre Hospital (Université Paris-Saclay) during their standard of care and treated with methotrexate (MTX) or JAKinibs. All patients fulfilled the ACR/EULAR 2010 criteria. The MTX treated group (n = 9) were 89% female and the JAKinib group (n = 16) was 58% female. The average age in the MTX group was 65 years old and in the JAKinib group the average age was 58 years old. Demographics are shown in S1 and S2 Tables.

### *In vitro* studies

We isolated, expanded, and froze MSCs from whole labial salivary glands in standard culture media [34,35]. We cultured primary pooled HUVECs (ATCC, Manassas, VA) at a starting seeding density of 3,000–5,000 cells/cm^2^ in accordance with the manufacturer’s recommendations. We determined optimal concentrations of IFNγ and JAKinibs in dose-finding experiments. MSCs and HUVECs (n = 1–6, depending on the experiment) were treated with the following reagents in the described experiments: IFNγ (10 ng/mL), ruxolitinib (1 µM unless otherwise specified), baricitinib (1 µM unless otherwise specified), CHZ868 (1 µM unless otherwise specified). Ruxolitinib and the other JAKinibs used for this study are Type I inhibitors that interact with the active JAK conformation. CHZ868 is a Type II JAK inhibitor that interacts with JAK2 in the inactive conformation. After drug exposure, we washed the cells with PBS and treated them with standard growth media with 0.01% DMSO, 10 ng/mL final concentration IFNγ + 0.01% DMSO, or 10 ng/mL IFNγ + JAKinib for 48 hours. For withdrawal conditions, we washed the cells twice and then treated them with standard growth media with either 0.01% DMSO or with 10 ng/mL IFNγ + 0.01% DMSO for a variety of time periods before harvest. In all cases, we harvested the cells by collecting the conditioned media, washing once with PBS on ice, removing PBS, and conducting on-plate lysis by scraping the cells from each 10 cm plate into 600 μL of 1x lysis buffer (Cell Signaling Technology, Danvers, MA) supplemented with 1 mM EDTA, 1 mM PMSF, 1 mM NaF and 1 mM Na_3_VO_4_.

### Western blot

After running the gel and transferring to a membrane, we blocked the membranes with 5% non-fat dry milk in TBST (for phospho and total TYK2, 3% BSA in TBST was used per manufacturer instructions) at room temperature for ~ 1 hr. We diluted primary antibodies to 1:1,000 per manufacturer’s recommendations and incubated them overnight at 4° C. After washing and incubation with HRP-conjugated secondary, we took images on an Amersham ImageQuant 800 (Cytiva, Marlborough, MA). We performed densitometric analysis using ImageStudio Lite software (Li-Cor, Lincoln, NE). Primary antibodies are listed in Supplemental Methods. All raw western blots are included in S1 Fig.

### RNA seq

We grew cells (n = 3 biological replicates in each condition) as previously described and treated them as indicated with IFNγ/JAKinibs. After trypsinization, the cells were frozen and shipped to MedGenome, Inc. (Foster City, CA) for RNA isolation, reverse transcription, library construction, sequencing, and bioinformatics. The library was prepared using Illumina TruSeq stranded mRNA kit and sequenced with NovaSeq. Alignment was performed with STAR (v2.7.3a). Ribosomal and mitochondrial genome reads were removed. Raw read counts were established with HTSeq (v0.11.2) and read counts were normalized with DESeq2. The aligned reads were used to estimate gene expression with cufflinks (v.2.2.1). Read distribution, strand specificity, and gene body coverage were calculated. Splicing annotation was performed at the splice event level and splice junction level and aligned reads were used to estimate gene expression with cufflinks v2.2.1. and reported in fragments per kilobase per million for each gene. Differential expression analysis was performed with DESeq2 (R Bioconductor) and marked as significant differential expression if they achieved p value <0.05 and fold change ≥2 or ≤ −2. Bioinformatics included read quality assessment (base quality score distribution, sequence quality score distribution, average base content per read, GC distribution, PCR amplification, check for over-represented sequences, and adapter trimming) with FastQC (v0.11.8) and fastq-mcf program (v1.05) and cutadapt (v2.5). Over Representation Analysis (ORA), was performed for significantly differentially expressed genes using DAVID (v6.8) with R Bioconductor package clusterProfiler (v3.14.3). Gene ORA was done with enrichGO from cluster Profiler using the organism level genome wide annotation package from Bioconductor and provides enrichment GO categories filtered on results by p value <0.05. Enrichplot (v1.8.1) was used to generate the heatmap. The data/analyses presented in the current publication have been deposited in and are available from the dbGaP database under dbGaP accession phs003915.v1.

### Real time quantitative PCR

After harvesting the MSCs (n = 3 biological replicates) in each treatment condition in TRIzol^®^, we used the Direct-zol RNA Miniprep columns for RNA isolation (Zymo Research, Irvine, CA). We generated cDNA using SuperScript IV reverse transcriptase (Invitrogen, Waltham, MA) per the manufacturer’s recommendations. We performed qPCR using the QuantiNova SYBR Green kit (*PLAU, PLAT*) (Qiagen, Germantown, MD) or the qPCR 2x Green Master Mix (*IFIT1*, *MX1*, *MX2* and *TNFSF15*) (Reagents and Chemicals). Primers sequences are shown in S3 Table. ΔΔC_t_ values (ΔΔC_t_ = ΔC_t,IFNγ maintained_-ΔC_t,__) are plotted in the figure, and ΔC_t_ values were used for determining statistical significance.

### ELISA

We performed enzyme-linked immunosorbent assay (ELISA) on conditioned media collected above the adherent cells. We then performed the uPA ELISA kits per the manufacturer’s instructions (for the MSC experiment, Innovative Research, Novi, MI; for the HUVEC experiment, CUSABIO, Houston, TX).

### Crosslinking assays

PBMCs from RA patients (n = 25) were collected the day of sampling and cultured overnight in RPMI with the addition of 10% FBS, 1% sodium pyruvate, 1% Hepes, 1% MEM, 1% penicillin/streptomycin and 200 UI/mL of IL-2.The next day, PBMCs were stimulated by crosslinking [38]. PBMCs were seeded on 96-well plates (Maxisorb, Thermofisher Scientific) previously coated with anti-CD16 (BD Bioscience) or isotype control and incubated at 37°C 5% CO2 for five hours with IL-2 (10 UI/mL). Golgistop was added after 1h. We then washed PBMCs and stained them for FACS analysis. We assessed intracellular IFNγ and TNFα after permeabilization.

### FACS analysis

We performed flow cytometry on FACS Canto II (Beckman Coulter) and conducted analysis using FlowJo software. NK cells were gated as CD56 + CD3- among living cells. Results were expressed as a percentage of positive cells for each marker. The antibodies used are indicated in S4 Table.

### Statistical analysis

For statistical testing of total and pSTAT and pJAK dose response studies, ELISA, and cross-linking studies, we performed an ANOVA for parametric and a Kruskal-Wallis for nonparametric multiple-comparisons. We used Bonferroni’s correction for multiple testing. For qPCR, we computed ΔΔC_t_ statistical significance using two-sample unpaired Student’s t-test on ΔC_t_ values. Statistical analysis was carried out by GraphPad Prism software (Graphad, Software, La Jolla, CA).

## Results

### The type I JAKinib, ruxolitinib, promotes pJAK1 and pJAK2 phosphorylation

We focused first on MSCs for our in vitro studies because these stromal cells are present in the adventitia of blood vessels, which is directly associated with infiltrating immune cells [39]. Ruxolitinib increased IFNγ-induced phosphoJAK1 (pJAK1) and pJAK2 relative to total JAK1 and JAK2. Peak phosphorylation of JAK1 and JAK2 occurred around 24 hours and pSTAT suppression occurred immediately (Fig 1B-D). Increased doses of ruxolitinib decreased total JAK2, not total JAK1 (Fig 1E, F). Phosphorylation of JAK1 and JAK2 relative to total JAK increased in a dose-dependent fashion starting at around 50nM of ruxolitinib (Fig 1E, F). Phosphorylation of STAT1 was appropriately suppressed relative to total STAT1 (Fig 1G). We found no differences in JAK/STAT phosphorylation patterns between SjD and control MSCs (S2 Fig).

pSTAT2–5 and pTYK2 were suppressed with the addition of ruxolitinib to IFNγ, but we did find a subtle increase in C-jun and cyclin D1 1–2 hours after the addition of ruxolitinib (S3 Fig). Ruxolitinib alone, without the presence of IFNγ, did not increase the expression of any of our tested pathways. Similarly, IFNγ treatment alone did not substantially increase pJAK1 and pJAK2 compared to ruxolitinib withdrawal conditions; however, pJAK1 and pJAK2 were greater in IFNγ alone compared to the vehicle or ruxolitinib treatment (S4 Fig). These results show that the addition of ruxolitinib to IFNγ increased pJAK1 and pJAK2 phosphorylation, confirming ruxolitinib is a Type I JAKinib capable of promoting phosphorylation of JAK1 and JAK2.

### While type I JAKinibs promote JAK phosphorylation, type II JAKinibs do not

**Fig 2:**
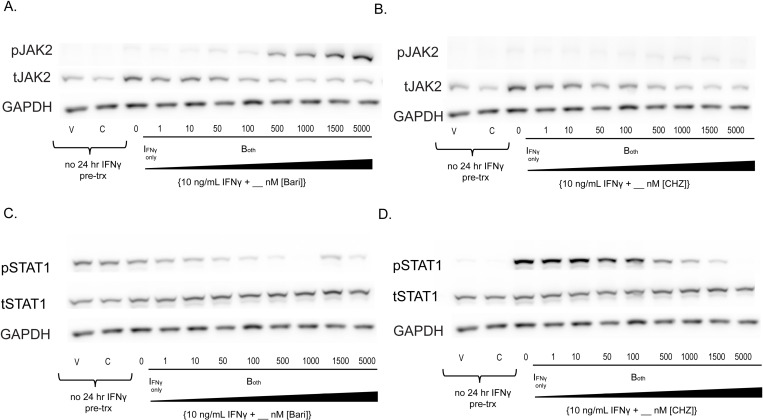
Type I JAKinibs promote JAK phosphorylation while Type II JAKinibs do not. We treated SG-MSCs with vehicle, JAKinib alone (C = CHZ868; B = baricitinib), or varying doses of baricitinib or CHZ868 with IFNγ (10 ng/mL). After 48 hours, the cells were harvested, and protein was isolated for western blot to the shown targets. Each treatment condition shown used MSCs from a control subject. **(A)** There was a slight decrease in total JAK2 with increasing barictinib, but a more pronounced increase in pJAK2 relative to total JAK2; (B) total JAK2 decreased with increasing CHZ868, but there is no pJAK2 phosphorylation relative to total JAK; (C) increased concentrations of baricitinib did not affect total STAT1 but drove a dose-related reduction in pSTAT1; (D) increased concentrations of CHZ868 did not affect total STAT1 but drove a dose-related reduction in pSTAT1.

Using another Type I JAKinib, baricitinib, we found that increased baricitinib dose also increased pJAK2 relative to total JAK2 (Fig 2A), similar to ruxolitinib. We showed that CHZ868 did not increase pJAK2 (Fig 2B). As expected, both baricitinib and CHZ868 decreased pSTAT1 in a dose dependent manner (Fig 2C-D). We selected 1000 nM JAKinib for the remainder of our experiments because it was the lowest concentration associated with clear reduction of pSTAT1.

### Type I JAKinib withdrawal causes a transient surge in pSTAT, type II JAKinib withdrawal does not

**Fig 3:**
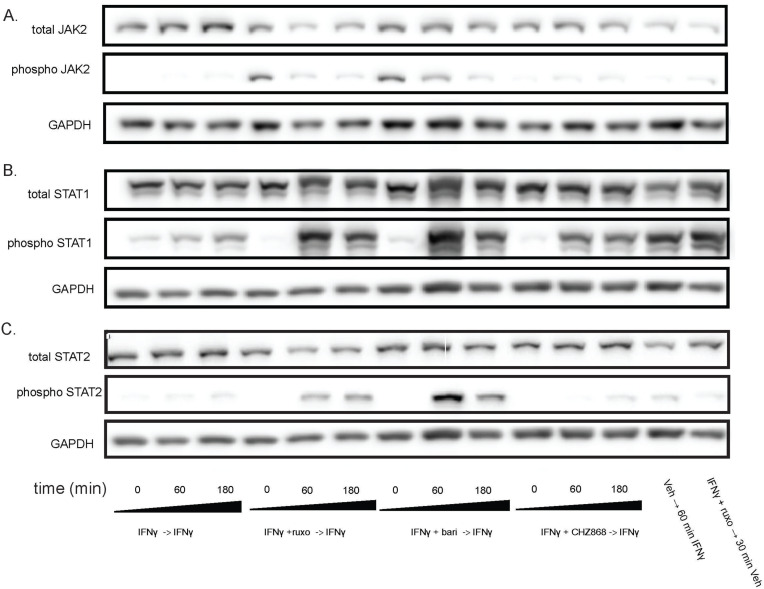
Withdrawal of Type I JAKinibs increased STAT phosphorylation, a phenomenon present but markedly lower in Type II JAKinibs. We treated MSCs with IFNγ 10ng/mL then added IFNγ alone, IFNγ and ruxolitinib (1 µM), IFNγ and barictinib (1 µM), or IFNγ and CHZ868 (1 µM). After 48 hours, we removed the medium, washed the cells, and added back media with only IFNγ. Cells were harvested at the indicated time points and western blot was performed on the resulting protein lysates. GAPDH was used as a control. Each treatment condition shown used MSCs from a control subject. (A) pJAK2 peaked at time zero after exposure to baricitinib and ruxolitinib and then decreases. There was no increase in pJAK2 with CHZ868 treatment; (B) pSTAT1 proportion peaked after 60 minutes and was greater after Type I JAKinib withdrawal than Type II JAKinib withdrawal; **(C)** The pSTAT2 proportion peaked 60 minutes after Type I JAKinib withdrawal but this phenomenon was not seen with Type II JAKinib withdrawal.

After serum starvation, we treated MSCs with IFNγ for 24 hours and treated the cells with IFNγ and a JAKinib (ruxolitinib, baricitinib, or CHZ868) for 48 hours. Then we withdrew the JAKinib from each condition while continuing IFNγ. We found that the withdrawal of ruxolitinib and baricitinib led to decreased pJAK2 over 3 hours (Fig 3A) and a marked increase in pSTAT1 (Fig 3B). In contrast, CHZ868, which lacked increased pJAK2 at baseline, had near undetectable levels of pJAK2 (Fig 3A) and a slight increase in pSTAT1 (Fig 3B). Interestingly, the expression of pSTAT1 appeared greater with ruxolitinib and baricitinib withdrawal than treatment of MSCs with 10 ng/mL IFNγ.

We tested pSTAT2–5 and found that hyperphosphorylation of STAT on Type II JAK inhibitor withdrawal was most pronounced with STAT2 but also occurred in STAT3 and 5 (Fig 3C, S5 Fig). We then tested whether the withdrawal hyperphosphorylation phenomenon affects other related pathways [40] because IFN also can induce JAK to activate the MAPK and PI3K/AKT systems. We found that although both phospho-ERK and phospo-Akt increased after withdrawal, there was no clear association with either a Type I or Type II JAKinib-specific response (S5 Fig).

### JAK hyperphosphorylation and STAT phosphorylation after withdrawal of type I JAKinibs occur in other cell types

**Fig 4:**
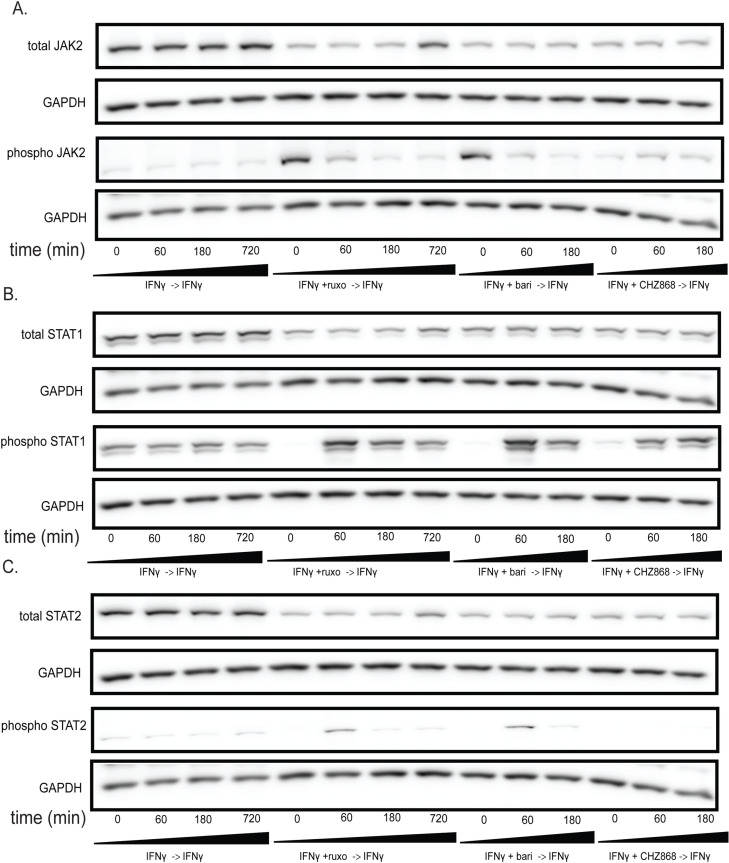
The JAKinib withdrawal phenomenon observed in MSCs can be extended to other cell types. We treated HUVECs with IFNγ for 24 hours then proceeded with the following conditions: IFNγ alone, IFNγ and ruxolitinib (1 µM), IFNγ and barictinib (1 µM) or IFNγ and CHZ868 (1 µM). After 48 hours, we removed the medium, washed the cells, and added back media with only IFNγ. Cells were harvested at the indicated time points and western blot was performed on the resulting protein lysates. GAPDH was used as a control. Each treatment condition used a primary pooled ATCC HUVEC cell line. (A) pJAK2 peaked at time zero after exposure to baricitinib and ruxolitinib and then decreases. There was no increase in pJAK2 with CHZ868 treatment; (B) pSTAT1 proportion peaks after 60 minutes and was greater after Type I JAKinib exposure than Type II JAKinib exposure; **(C)** The pSTAT2 proportion peaked 60 minutes after Type I JAKinib withdrawal but this phenomenon was not seen with Type II JAKinib withdrawal.

We selected HUVECs as representative endothelial cells as they are an important site for vascular remodeling in cardiovascular disease [39]. We found that HUVECs showed increased JAK2 phosphorylation with exposure to Type I JAK inhibitors but not Type II JAK inhibitors (Fig 4A). On JAKinib withdrawal, pSTAT1 increased more in Type I than Type II JAKinibs (Fig 4B). Finally, JAKinib withdrawal led to marked increases in pSTAT2 after Type I withdrawal than after Type II withdrawal (Fig 4C).

### IFN and PLAU (urokinase) increases with type I JAKinib withdrawal

**Fig 5:**
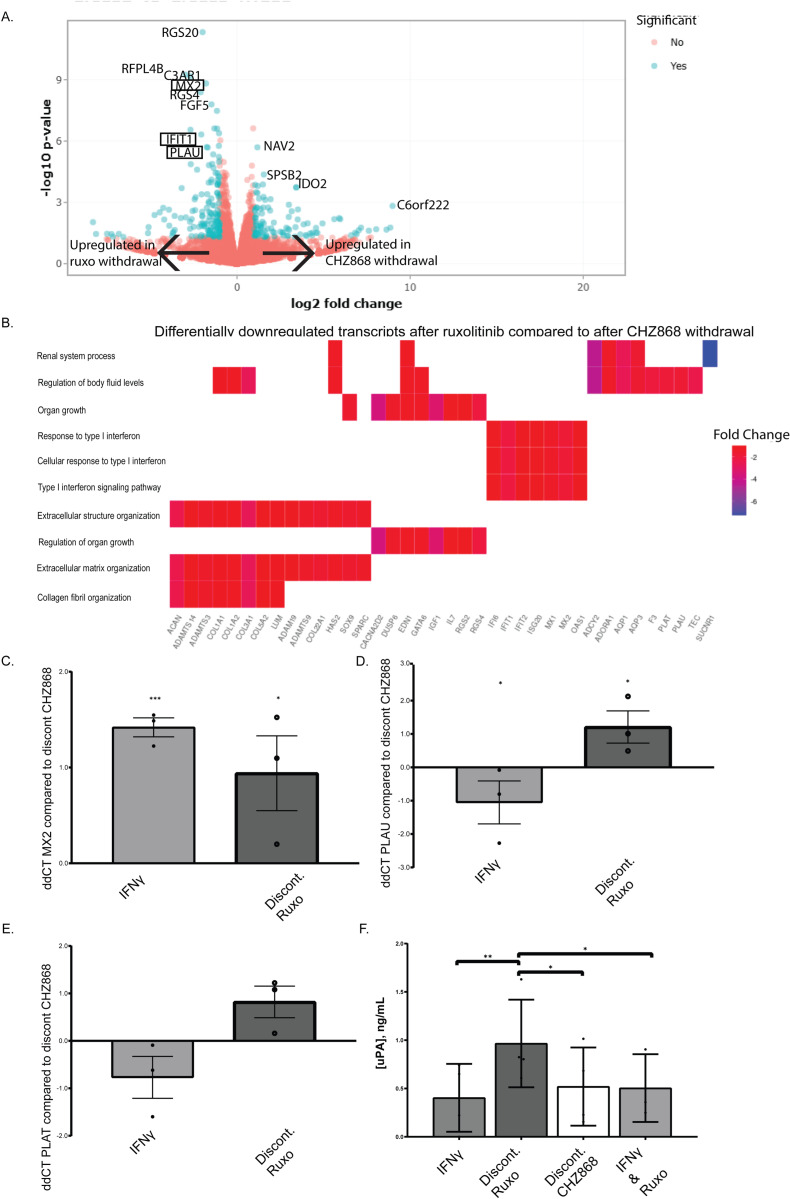
IFN and PLAU (urokinase) are increased after ruxolitinib compared to CHZ868 withdrawal. **(A-B)** Bulk RNA seq was performed on MSCs that were exposed to the following treatments: IFNγ 10 ng/mL and ruxolitinb (1000 nM), cells were washed, then continue treatment with IFNγ only (ruxolitinib withdrawal) or IFNγ and CHZ868 (1000 nM) treatment. Cells were washed again and treatment was continued with only IFNγ (CHZ868 withdrawal). MSCs from control subjects were used for each condition (n = 3 each condition). The volcano plot shows significantly upregulated and downregulated transcripts. The heatmap shows differentially downregulated pathways in ruxolitinib compared to CHZ868 withdrawal as Gene Ontology Over Representation Analysis (ORA); **(C)** RT-qPCR of RNA isolated from MSCs (n = 3 each condition) after treatment in the above mentioned conditions and harvested 3 hours later showed MX2 transcripts were significant higher after ruxolitinib withdrawal than after CHZ868 discontinuation; **(D)** RT-qPCR of MSCs (n = 3 each condition) after treatment in the above conditions and harvested 3 hours later showed PLAU transcripts were significantly higher after ruxolitinib withdrawal than after CHZ868 discontinuation; (**E)** RT-qPCR of MSCs (n = 3 each condition) after treatment in the above conditions and harvested at 3 hours post-withdrawal showed that PLAT transcripts trended toward being higher after withdrawal of ruxolitinib than after CHZ868 discontinuation; (**F)** ELISA of conditioned medium aspirated from MSCs (n = 3 each condition) after 3 hours of above treatment conditions showed that PLAU is significantly increased in the culture media of MSCs after ruxolitinib withdrawal compared to other conditions; statistical significance was determined via an ANOVA parametric model with multiple-comparison testing using Bonferroni’s correction. *p < 0.05; ** p<=0.01; ***p < 0.001; ΔΔC_t_ values (ΔΔC_t_ = ΔC_t,DC CHZ_-ΔC_t,__) are plotted in the figure and statistical significance was computed by two-sample unpaired Student’s t-test on ΔC_t_ values compared to CHZ868 withdrawal conditions.

Our findings thus far suggest that withdrawal of JAKinib leads to increased pSTAT, but the consequences of an increased active STAT cascade remained unknown. Given the fact that MACE is a concerning adverse effect associated with JAKinibs, we sought to determine what downstream pathways from pSTAT might contribute to this risk. To obtain a global view of the differential transcriptional pathways between Type I and II JAKinib withdrawal, we performed RNA sequencing comparing 3 hour withdrawal of ruxolitinib to 3 hour withdrawal of CHZ868. We found a total of 254 significantly differentially expressed genes between these conditions (n = 102 were upregulated and n = 152 were downregulated in ruxolitinib compared to CHZ868 withdrawal) (Fig 5A; S5 Table). We found that Type I IFN signaling pathway transcripts were downregulated in CHZ868 withdrawal relative to ruxolitinib withdrawal (Fig 5B). We confirmed that IFN-related MX2 transcripts were significantly higher after ruxolitinib withdrawal than CHZ868 withdrawal (Fig 5C). In addition, MX1 transcripts were upregulated with ruxolitinib withdrawal compared to CHZ868 discontinuation, but this did not achieve statistical significance (S6 Fig A-C).

We found that tissue plasminogen activator [PLAT; tPA] and urokinase-type plasminogen activator [PLAU; uPA] transcripts were downregulated in CHZ868 withdrawal relative to ruxolitinib withdrawal (Fig 5A; S5 Table) in our RNA sequencing results. Because PLAT and PLAU are also associated with atherosclerosis/thrombotic pathways (Fig 5B) and JAKinib therapy was associated with MACE, we focused on these transcripts for further analyses. We confirmed PLAU transcripts increased more with ruxolitinib withdrawal than CHZ868 withdrawal by qPCR and saw a diminished but similar trend with PLAT (Fig 5D-E). We found that after ruxolitinib withdrawal, MSCs made significantly more uPA than after CHZ868 withdrawal (Fig 5F). Conditioned media assessment of uPA from HUVECs was below the level of detection, indicating that the STAT signaling cascade produced by JAKinib withdrawal might have cell-specific consequences.

### IFNγ and TNFα related inflammatory transcripts are increased after type I JAKinib withdrawal

We found that Type I JAKinib withdrawal *in vitro* caused a pSTAT signaling cascade that ultimately resulted in increased interferons and urokinase. We next needed to determine the effect of Type I JAKinib withdrawal in humans. To address this gap, we studied NK cells derived from patients treated with Type I JAKinibs. We focused on NK cells because they might contribute to autoimmunity through exposure of autoantigens, promoting maturation of dendritic cells and differentiation of monocytes [41,42]. Furthermore, NK cells are a major producer of IFNγ and are activated by Type I IFNs, linking their relevance in autoimmunity to our observed findings of increased IFN after JAKinib withdrawal [43,44]. The JAK/STAT pathway is highly involved in these cells, making them a good model to study the effect of Type I JAKinib withdrawal in patients.

**Fig 6:**
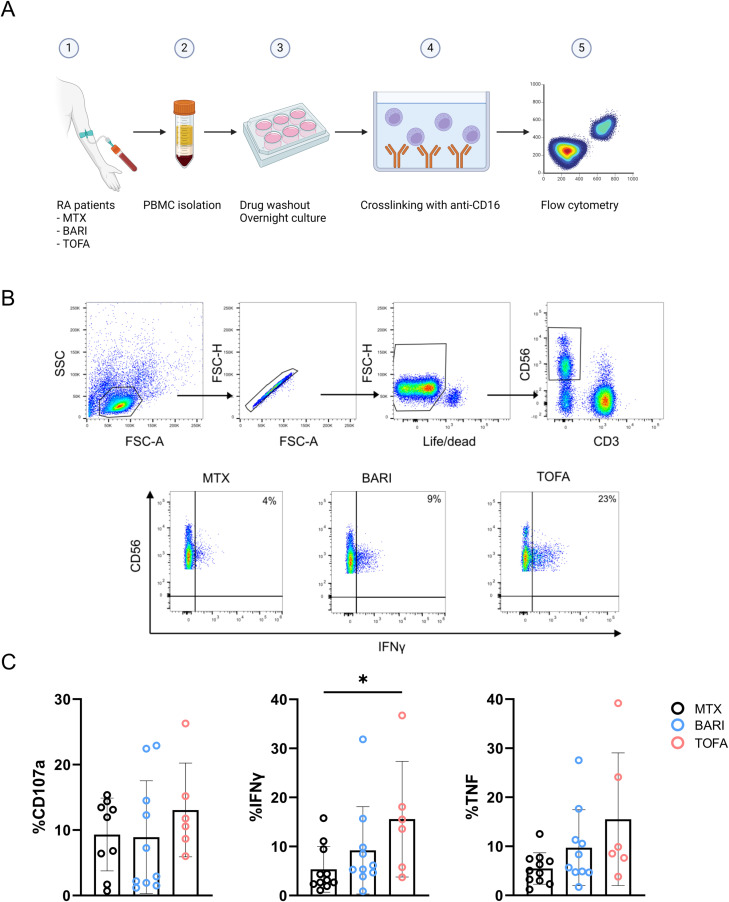
JAKinib withdrawal results in patient-derived NK cell activation. PBMCs from RA patients treated with JAKinibs (tofacitinib [TOFA] or baricitinib [BARI]) or methotrexate [MTX] were stimulated upon CD16 by crosslinking for 5 hours after overnight withdrawal of the drug. CD107a and intracellular IFNγ and TNFα were assessed by flow cytometry. **(A)** Schematic for patient PBMC crosslinking; (B) flow cytometry gating strategy. NK cells were defined as CD3-CD56 + ; (**C)** CD107a, IFNγ and TNFα expression by flow cytometry. NK cells from patients treated with JAKinibs expressed more IFNγ and those treated with JAKinibs trended to greater NK cell activation overall. Kruskal-Wallis test was used. *p < 0.05 was considered significant.

We analyzed the functionality of NK cells from RA patients treated with methotrexate, baricitinib, or tofacitinib after overnight drug withdrawal (Fig 6A). We simulated JAKinib withdrawal by allowing at least 12 hours of time to elapse since the last exposure to drug *in vivo* to testing of the collected NK cells. To test the effect of this withdrawal on NK cells, we stimulated PBMCs by crosslinking with anti-CD16 and assessed the degranulation marker CD107a and intracellular production of IFNγ and TNFα (Fig 6B). In addition, we found that NK cells from patients taking the Type I JAKinib tofacitinib expressed significantly more IFNγ than those taking methotrexate and there was a trend toward higher CD107a and TNFα expression (Fig 6C). These findings support that upon withdrawal from JAKinibs, NK cells demonstrate a pro-inflammatory profile.

## Discussion

Our results show that upon *in vitro* IFNγ exposure, Type I JAKinibs increase phosphorylation of JAKs compared to Type II JAKinibs. However, both similarly suppress downstream signaling pathways, suggesting similar efficacy in their known role as JAK-STAT signaling pathway inhibitors. Conversely, we revealed a divergent response to JAKinib withdrawal, where Type I JAKinib withdrawal resulted in a downstream STAT phosphorylation surge relative to Type II JAKinib withdrawal. This means that Type I JAKinibs initiate a STAT signaling cascade that is notably different than the signaling cascade seen after Type II withdrawal.

We found differential Type I and Type II JAKinib withdrawal signaling cascades, which resulted in greater IFN- and coagulation cascade-related transcription and protein production in the case of Type I inhibitors. Multiple studies have linked IFN treatment to adverse cardiac events and indicated that the most likely risk factor for cardiotoxicity with IFN exposure was previous cardiac disease [45–48]. This effect is theorized to be caused by increased oxygen demand from the flu like symptoms generated by IFN exposure leading to infarction or arrhythmia [45–48]. IFN might also potentiate atherosclerosis through several mechanisms including supporting foam cells, endothelial dysfunction, local immune cell activation, and NET formation, among other pathways [49,50]. IFNs are also particularly prothrombotic, reducing metalloproteinase-9 (MMP-9) and vascular endothelial growth factor [51]; however, mice treated with IFN-gamma blockade had higher levels of MMP-9 and VEGF, which resulted in faster thrombus resolution [52]. Thus, our finding of increased IFN production after JAKinib withdrawal, might explain the potentiated risk of MACE seen with JAKinib use among patients with atherosclerotic disease.

Additionally, we found Type I JAKinibs hyperphosphorylated JAKs and Type I JAKinib withdrawal resulted in a pSTAT cascade which also stimulated uPA production from stromal cells. This is problematic because uPA is associated with atherosclerosis in human coronary artery plaque [53], and research suggests uPA can promote plaque rupture as its receptor (the urokinase-type plasminogen activator receptor) is highly expressed in macrophages in ruptured plaques [54]. One study found uPA overexpression in mice caused intraplaque hemorrhage and fibrous cap disruption, potentially through overexpression of MMPs [55]. Another study found that transgenic mice overexpressing macrophage urokinase exhibited aortic tissue with inflammatory signaling molecules and proteins involved in cell adhesion, cytoskeleton, and apoptosis, leading to the loss of basement membrane proteins and plaque rupture [56]. Recent studies on humans with plaque rupture were analyzed by high throughput sequencing and revealed five major genes increased in PBMCs from patients with plaque rupture, two of these five genes were PLAU and PLAUR [57]. The authors of this study posit that hub genes such as PLAU and PLAUR serve as biomarkers for prospective prediction of atherosclerotic plaque rupture [57]. Finally, PLAU is increased in carotid plaques and associated with unstable plaque [58]. Thus, uPA elevation might be relevant to plaque rupture and thrombosis.

In addition to our *in vitro* findings of increased IFN and uPA after JAKinib withdrawal, we found that Type I JAKinib withdrawal promoted pro-inflammatory NK cells *ex vivo*, which suggest an increase in functionality and activation *in vivo*. These results differed from previous research that exposed *in vitro* NK cells to JAKinib without withdrawal, which led to their reduced functionality and activation [38]. We found that upon JAKinib withdrawal, NK cells are a source for Type II IFNs that are known to contribute to vascular disease.

**Fig 7:**
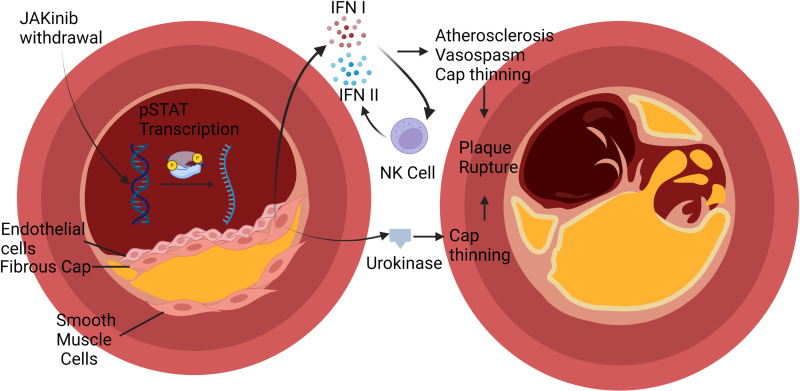
Graphical Abstract. In rheumatic disease patients, JAKinib withdrawal could drive a pro-inflammatory cascade that might contribute to major cardiac adverse events. Type I JAKinib exposure drives JAK hyperphosphorylation that is de-repressed upon JAKinib withdrawal. The subsequent spike in pSTAT signaling results in Type I IFN and urokinase production. Type I IFN activates NK cells toward a pro-inflammatory phenotype. Ultimately, JAKinib withdrawal might contribute to atherosclerosis and plaque rupture through IFN-mediated atherosclerosis, vasospasm, and fibrous cap thinning.

We established that, in vitro, Type I JAKinibs hyperphosphorylated JAKs and that on JAKinib withdrawal, a resulting pSTAT cascade increases IFN and uPA-related transcripts. Further, we showed that human NK cells cultured *ex vivo* after JAKinib withdrawal have increased activity and potentiate IFN production. We suspect that the combination of high Type I IFN, overexpressed urokinase, and increased pro-inflammatory NK cells might potentiate atherosclerosis and plaque rupture (Fig 7).

Type I JAKinibs are commonly used in patients with rheumatic disease and promote phosphate accumulation on the JAK whilst bound. Multiple varieties of Type I inhibitors might produce adverse MACE effects. For instance, baricitinib and tofacitinib cause increased thromboembolic risk in patients with thromboembolic risk factors (i.e., who use prothrombotic medications or have a history of a thromboembolic event) and advanced age comorbidities [59]. In addition, tofacitinib is associated with a 33% increased risk of MACE among patients greater than 50 years of age, with at least one cardiovascular risk factor [7]. In general, patients with rheumatic disease are already at increased risk for cardiovascular events due to established cardiovascular risk factors [3,4] and elevated pro-inflammatory cytokines that promote endothelial leukocyte accumulation resulting in increased platelet accumulation [51], and JAKinibs possibly potentiate this risk. JAKinibs might potentiate this risk by worsening traditional cardiovascular risk factors [11] or by downregulating specific pathways yet leaving other cytokines pathways unchecked. For example, though baricitinib reduced IL6 and IL12, it did not reduce IFN in SLE patients [60]. Thus, JAKinibs that target different pathways might have varying effects on cardiovascular disease; however, at least two studies have not found differences by JAKinib type on cardiovascular outcomes [61,62].

When patients begin withdrawing from Type I JAKinibs, their risk of MACE might increase due to the downstream effects of de-repression of phosphorylated JAK signaling, a resulting downstream STAT signaling burst, and the ultimate increase in pro-thrombotic cytokines, proteins, and inflammatory cells. Withdrawals can occur in patients for multiple reasons such as JAKinibs half-life, patients missing a dose, or discontinuation of use. For instance, a patient using immediate release tofacitinib with a half-life of three hours may have a serum level that decreases below the efficient doses for JAK inhibition, which could stimulate withdrawal and transiently stimulate IFN secretion. This could potentially increase the risk of MACEs. Possible ways to mitigate withdrawal effects could include the use of deuterated JAKinibs, which prolong their half-life, or use irreversible JAKinibs, which might avoid JAKinib withdrawal. Though not currently available for clinical use, Type II JAKinibs are currently being developed that might be available for clinical trials in the future [17]. Type II JAKinibs do not cause the downstream STAT signaling common to Type I JAKinibs, so also might avoid JAKinib withdrawal. Patients taking Type I JAKinibs should be advised on the importance of taking their JAKinibs regularly and tapering off these drugs rather than discontinued rapidly. This could reduce the frequency of withdrawal downstream STAT signaling cascades, ultimately reducing patients’ risk of MACE.

We acknowledge limitations to our study. We studied ruxolitinib and baricitinib *in vitro,* but it is possible that other JAKinibs that target other pathways, such as tofacitinib, would have behaved differently. Additionally, though the FDA and EMA consider JAKinibs a class that might cause MACE, studies confirming increased MACE with JAKinib use are contradictory and the exact risk of MACE with JAKinib use remains unclear. Though we identified higher IFN and urokinase production *in vitro* and increased NK cell activation *ex vivo* on Type I JAKinib withdrawal, we did not directly link these data to clinical thrombosis and cardiovascular disease. In our discussion, we draw possible associations between IFN, uPA and MACE using currently available literature. Studying the phenomenon of IFN and uPA and how this relates to MACE in humans is likely to be difficult given the low frequency of MACE overall. Therefore, additional work will need to be done in the future using mouse models to mechanistically prove that JAKinib withdrawal and the resultant IFN and urokinase elevation drive MACE.

In conclusion, we identify novel potential mechanisms related to Type I JAKinib withdrawal that might explain the MACE reported with JAKinib treatment in rheumatic diseases. These results might affect clinical practice; for example, experts might reconsider how they inform their patients on using these drugs and emphasize correct and timely doses, tapering, and potentially using Type II (should they become clinically available) or modified Type I JAKinibs such as those that are deuterated or irreversible. Future work in animal models is needed to mechanistically prove that JAKinib withdrawal and the resultant elevated IFNs and uPA drive MACE.

## Supporting information

S1 Fig Raw western blot images. (PDF)

S2 Fig Phosphorylation of JAKs and STATs is similar in SjD and control MSCs upon JAKinib application. We treated MSCs with vehicle, ruxolitinib (1 µM), IFNγ only (10 ng/mL), IFNγ then switching to ruxolitinib only, or IFNγ 10 ng/mL in combination with ruxolitnib 1 uM for varying periods of time or for 48 hours at varying doses of ruxolitinib (0-1500nM). At each time or dose of ruxolitinib, we harvested cells for protein and performed western blot for the indicated target. (A) total JAK1 remains stable with increasing concentrations of ruxo whereas phosphor JAK1 increases with total ruxo concentration; (B) total JAK2 peaks at 50 nM ruxo, subsequently decreasing, whereas pJAK2 increases with ruxo concentration; (C) Total STAT1 remains stable with increasing rux, whereas pSTAT1 is inversely correlated with ruxo dose. There is no difference in any condition between SjD (n = 3) and control (n = 3) MSCs. * < 0.05; *** = p < 0.0001.
(PDF)

S3 Fig Type I JAKinibs have appropriate downstream STAT inhibition. We treated MSCs with vehicle, ruxolitinib, IFNγ only, IFNγ then switching to ruxolitinib only, or IFN with ruxolitinib at varying times. Western blots with indicated antibodies are shown. Results demonstrate treatment of MSCs derived from a control subject.
(PDF)

S4 Fig pJAK1 and pJAK2 under longer exposure times show increased pJAK with IFNy only than vehicle or ruxo conditions. Control subject MSCs were treated with IFN gamma with ruxolitinib at stated doses. The results show western blots of total pJAK1 and pJAK2 with longer exposure times than those shown in Fig 2.
(PDF)

S5 Fig Withdrawal of JAKinib increases pSTAT more in Type I JAKinibs and more in PI3-AKT MAPK pathways, independent of JAK inhibitor type. Salivary gland MSCs were treated with IFN gamma for 48 hours. Ruxolitinib, baricitinib, or CHZ868 were added for 24 hours. The cells were washed and replaced with media containing only IFNγ for variable periods of time. GAPDH is the control for each condition. Results demonstrate treatment of MSCs derived from a control subject. Six conditions are shown: 1) IFNγ to IFNγ as a control; 2) ruxolitinib withdrawal; 3) baricitinib withdrawal; 4) CHZ868 withdrawal; 5) vehicle to IFNγ; 6) IFNγ and ruxolitinib to vehicle. The results show western blots of total STAT3–5, pSTAT3–5, total ERK, phosphoERK, total AKT, and phosphoAKT.
(PDF)

S6 Fig.
Ruxolitinib withdrawal increases inflammatory transcripts more than CHZ868. qPCR was performed after MSCs were exposed to the following treatments: IFNγ 10 ng/mL and ruxolitinib (1000 nM), wash cells, then continue treatment only with IFNγ (ruxolitinib withdrawal), or IFNγ and CHZ868 (1000 nM) treatment, wash cells, then continue treatment with only IFNγ (CHZ868 withdrawal). Panels A-C show ddCT of the displayed transcript in the demonstrated condition compared to discontinuation of CHZ868. Unpaired t-testing was performed between delta CT values of the displayed conditions compared to discontinuation of CHZ868. No results achieved significant difference thresholds.
(PDF)

S1 Table Characteristics of the patients from which MSCs were derived. (PDF)

S2 Table Characteristics of the patients from which NK cells were derived. (PDF)

S3 Table Primer sequences and targets. (PDF)

S4 Table Fluorochrome-labelled antibodies used for flow cytometry. (PDF)

S5 Table DEG after ruxolitinib withdrawal vs. CHZ868 withdrawal. (PDF)

Supplemental Methods Supplemental methods for the manuscript. (PDF)
