# Supplementary material for: JAK inhibitor withdrawal causes a transient pro-inflammatory cascade: A potential mechanism for major adverse cardiac events
Source: PLoS One. 2025 Jun 16;20(6):e0311706. doi: 10.1371/journal.pone.0311706 (PMC12169581; doi:10.1371/journal.pone.0311706)

S1 Fig 1B, tJAK1

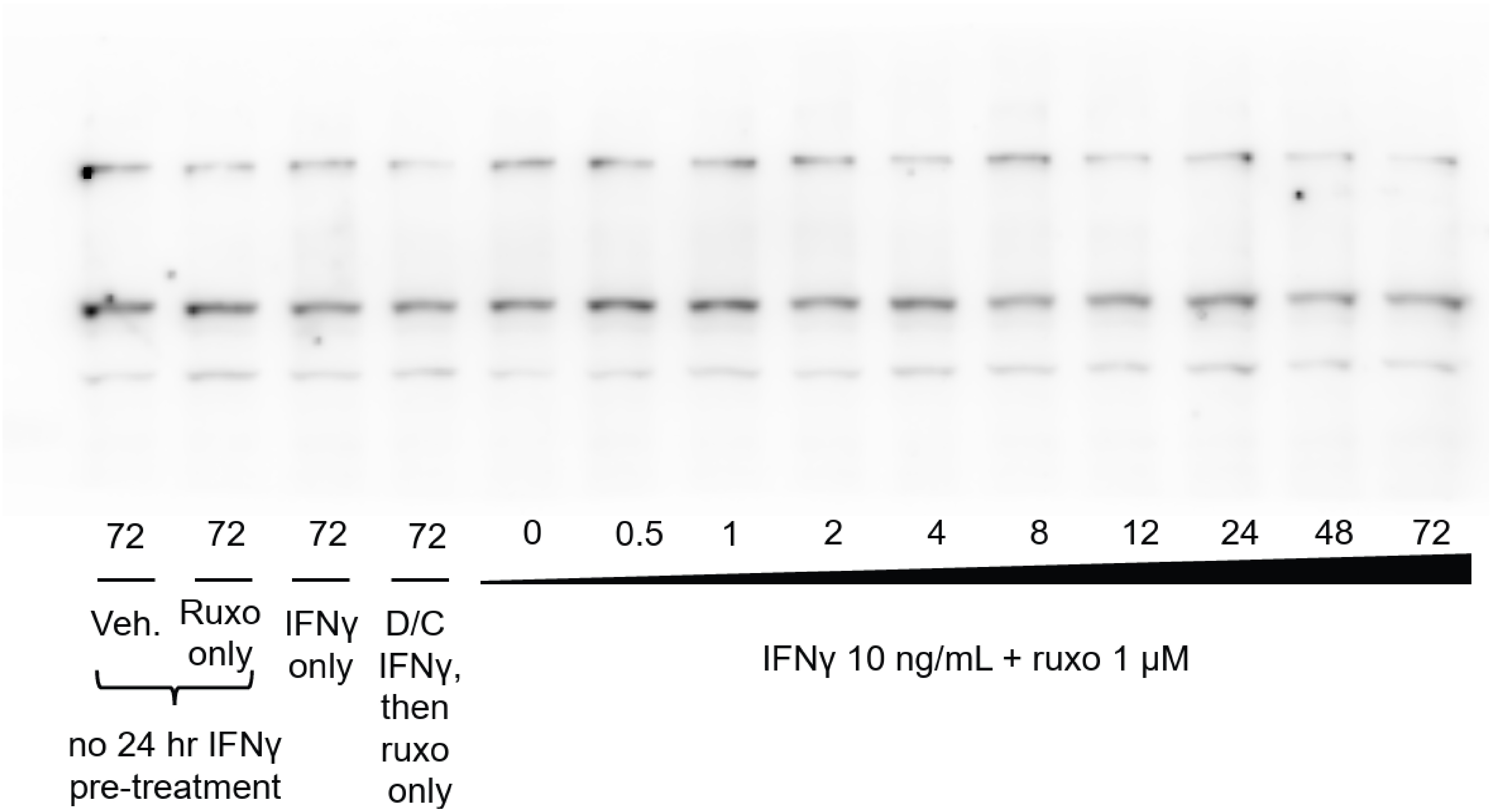

S1 Fig 1B, pJAK1

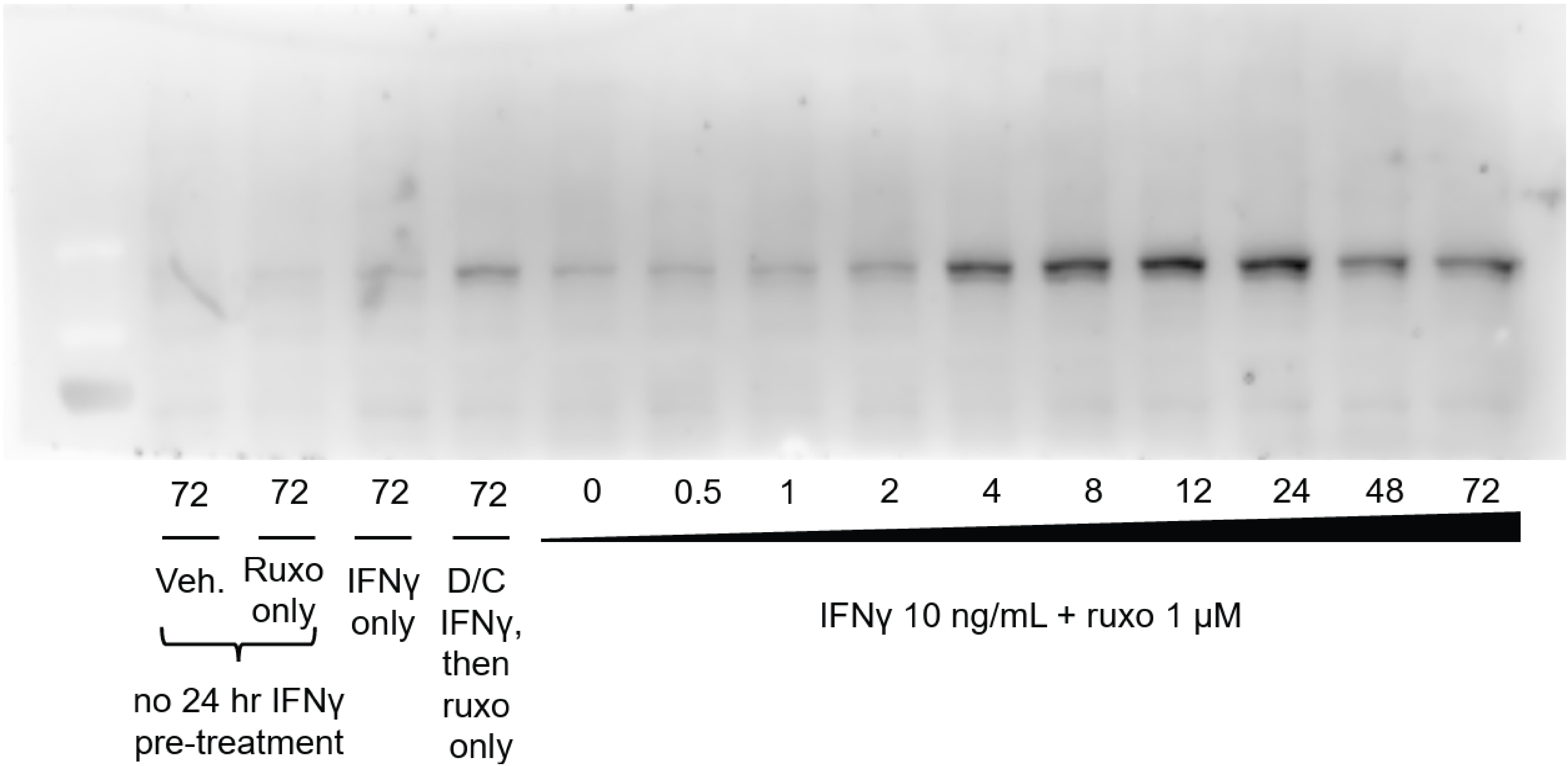

S1 Fig1B, GAPDH

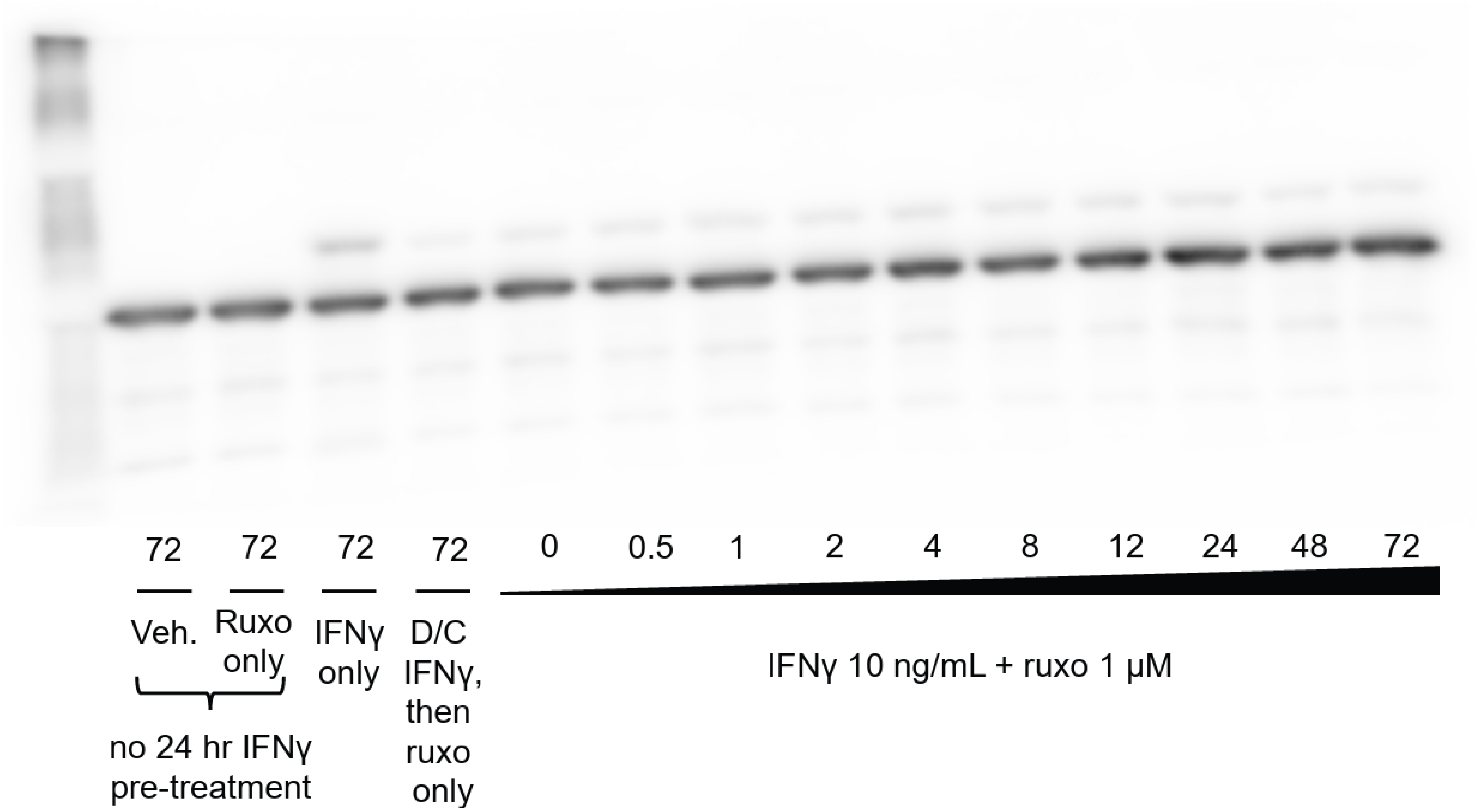

S1 Fig 1C, tJAK2

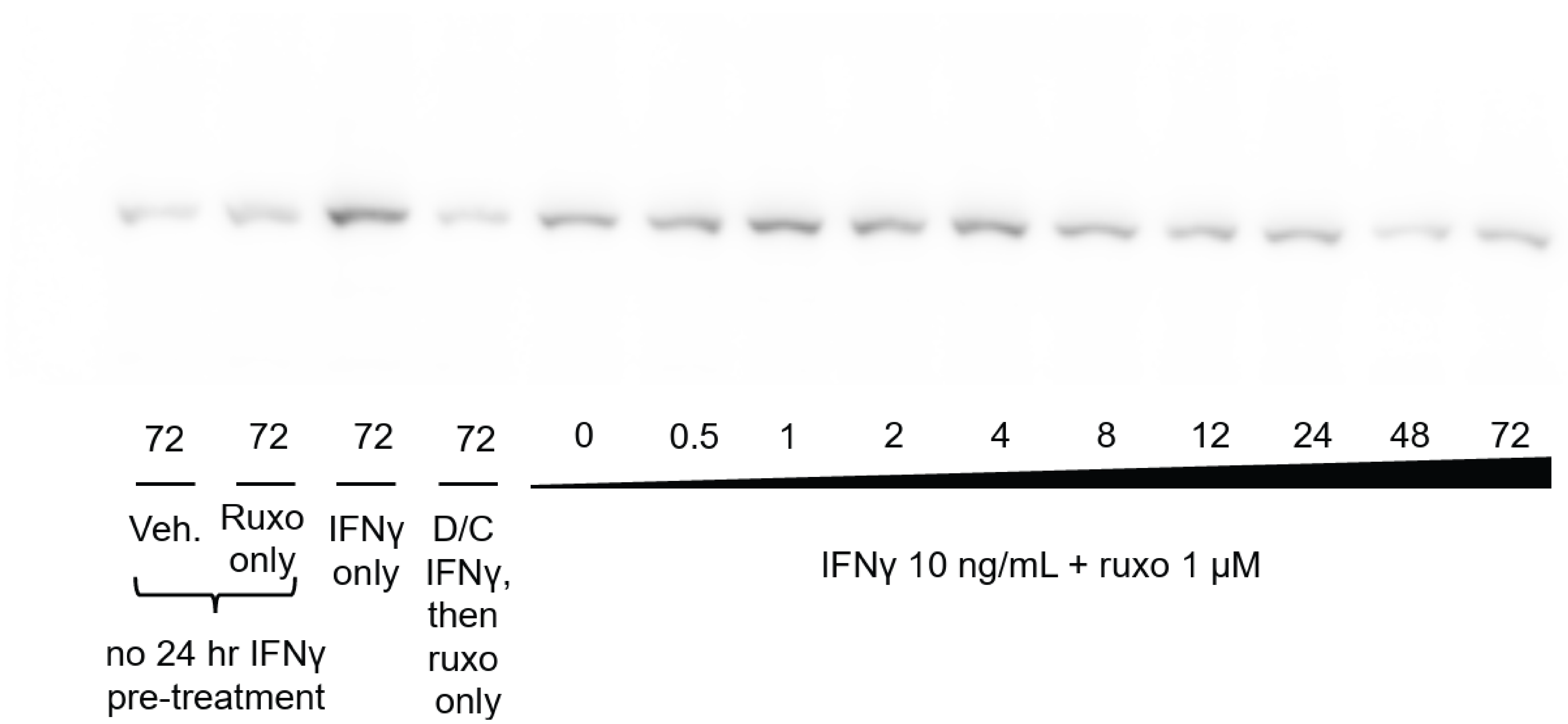

S1 Fig 1C, pJAK2

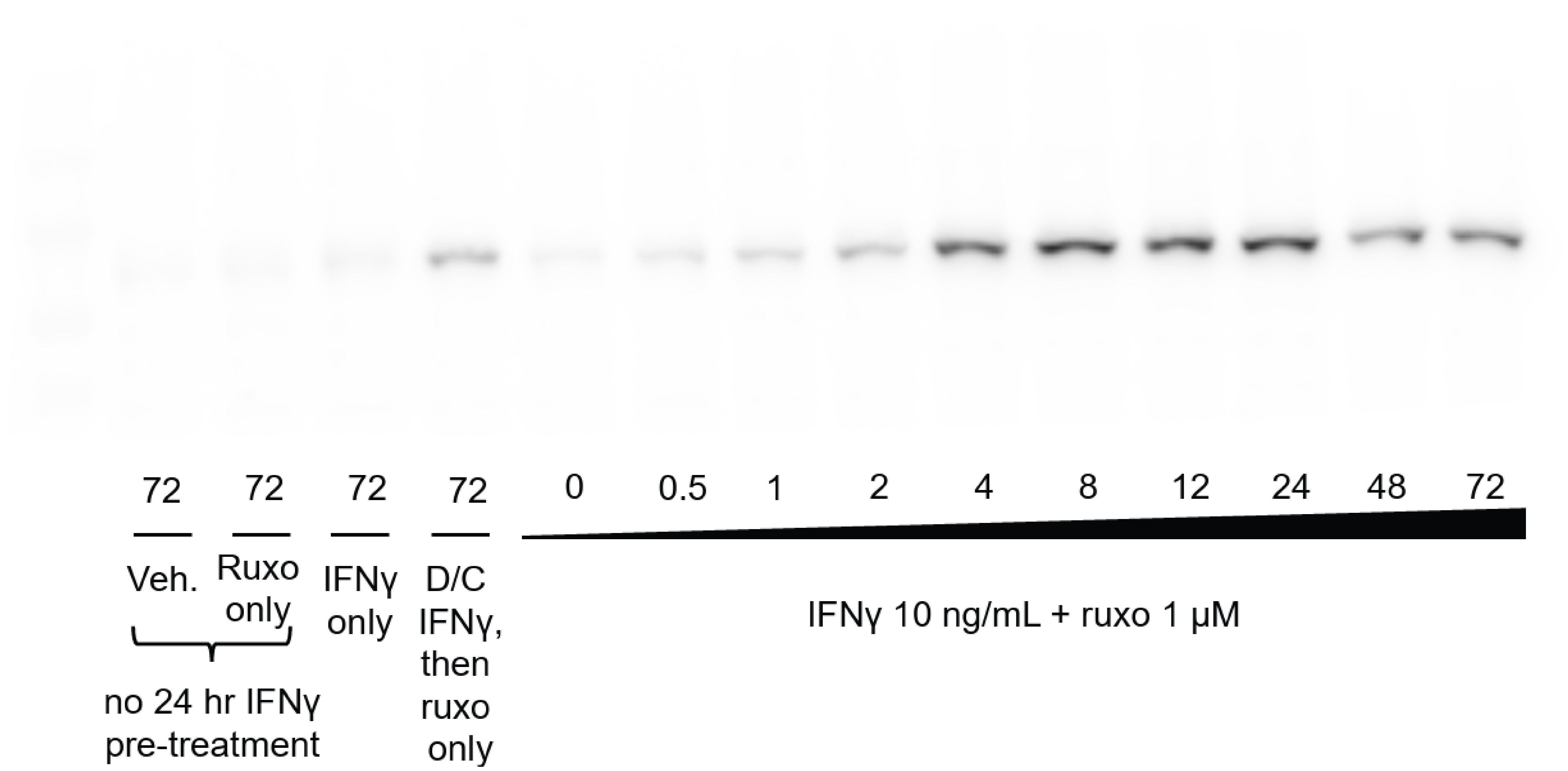

S1 Fig 1C, GAPDH

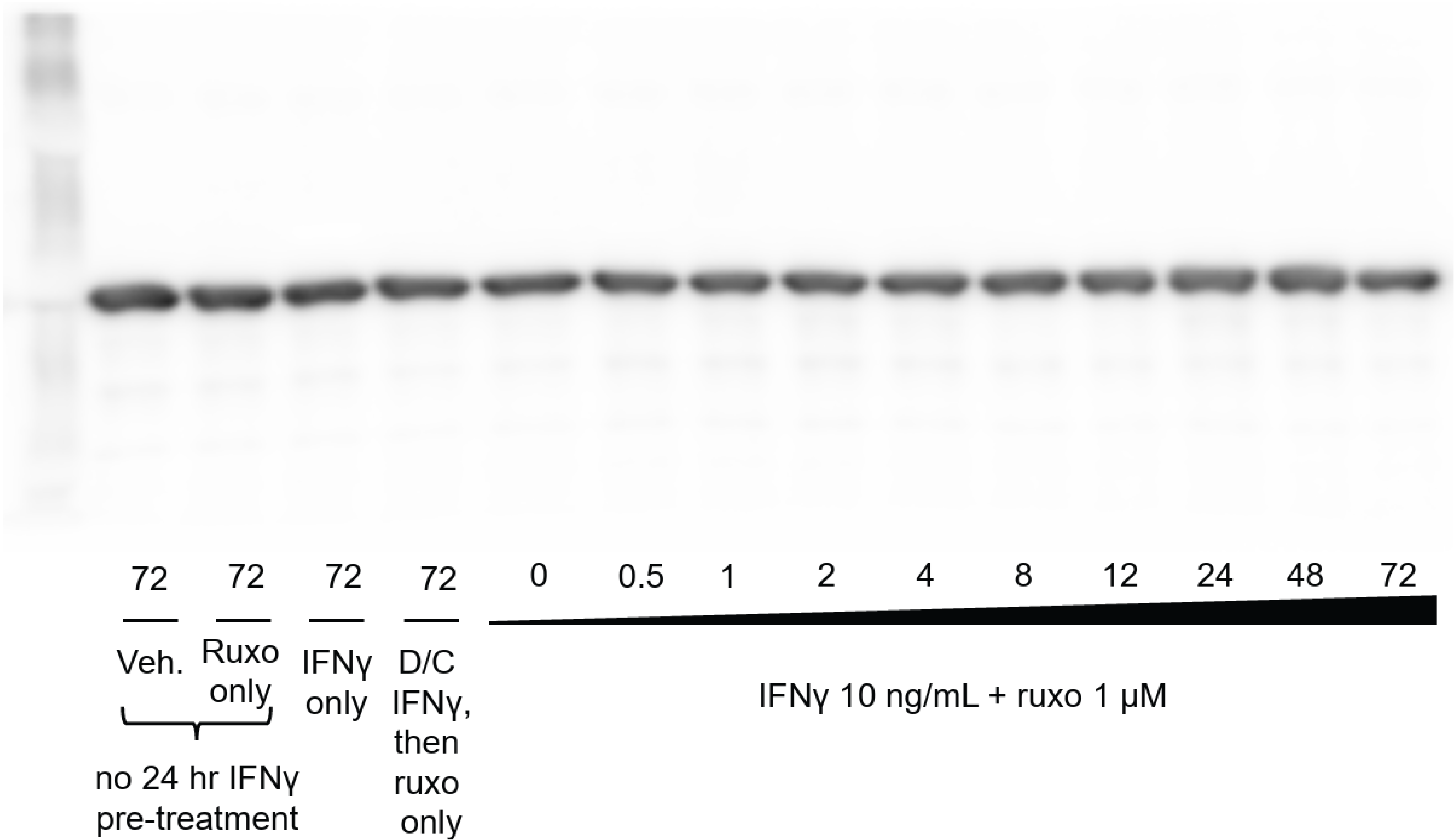

S1 Fig 1D, tSTAT1

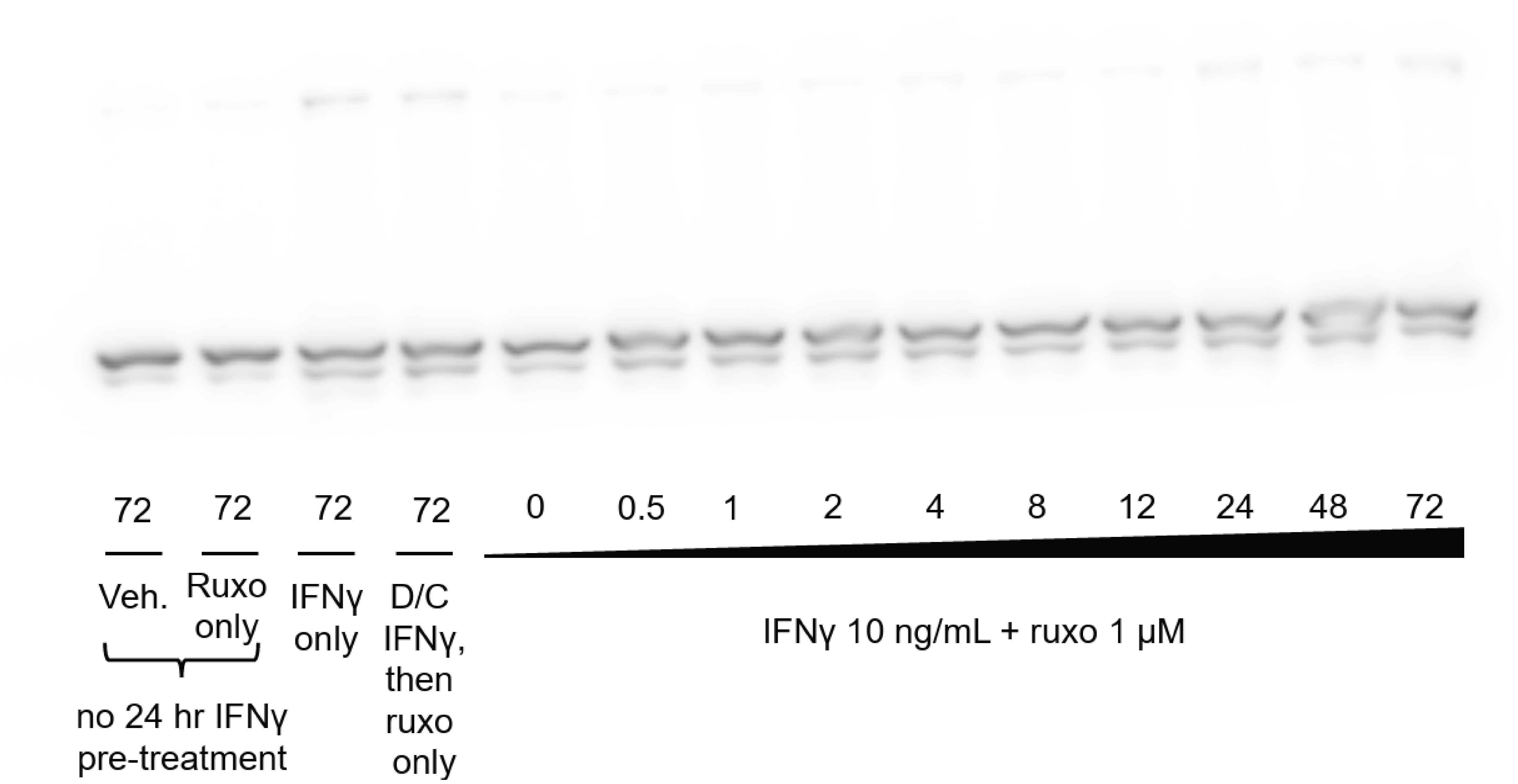

S1 Fig 1D, pSTAT1

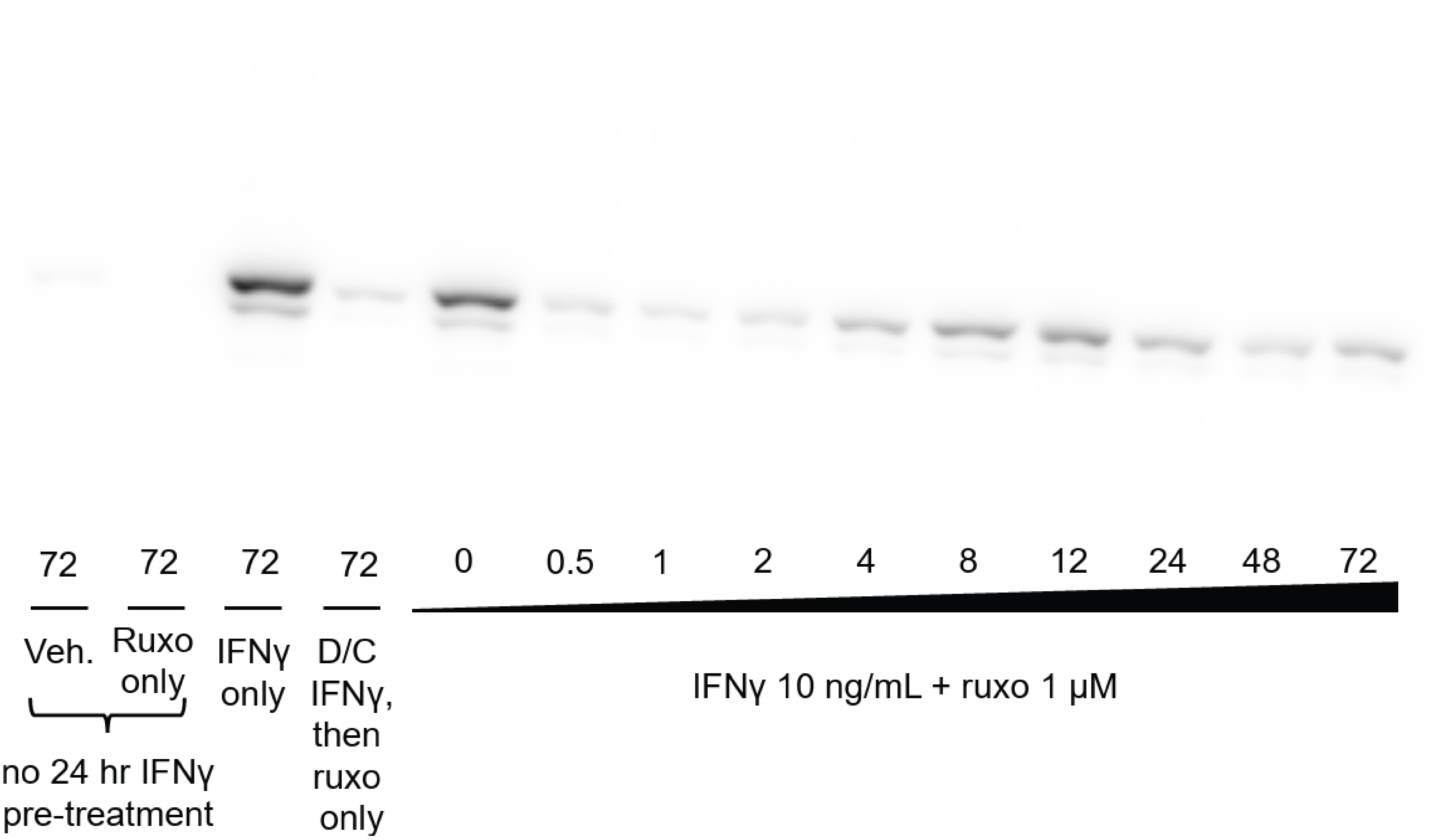

S1 Fig 1D, GAPDH

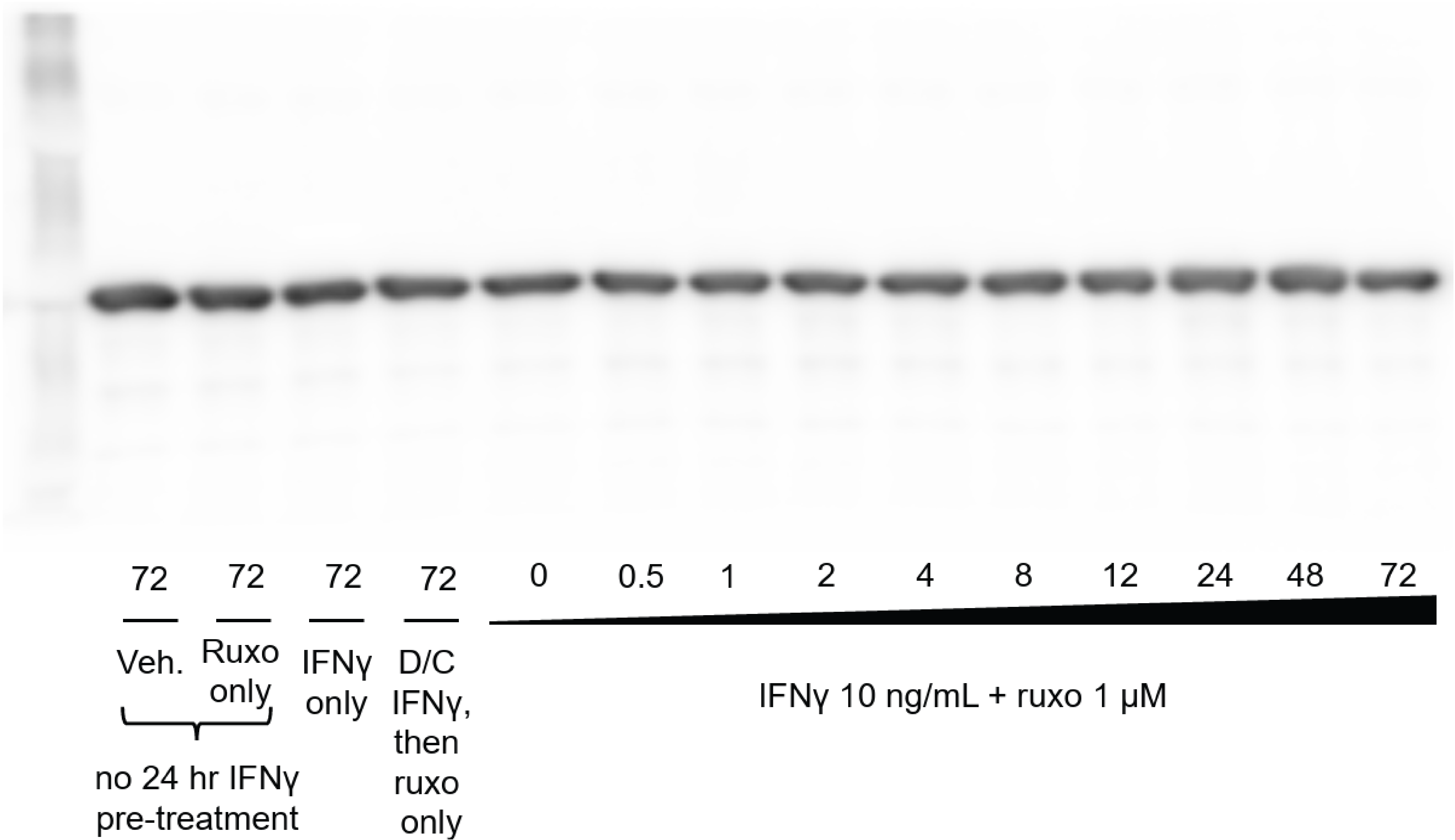

S1 Fig 1E, tJAK1

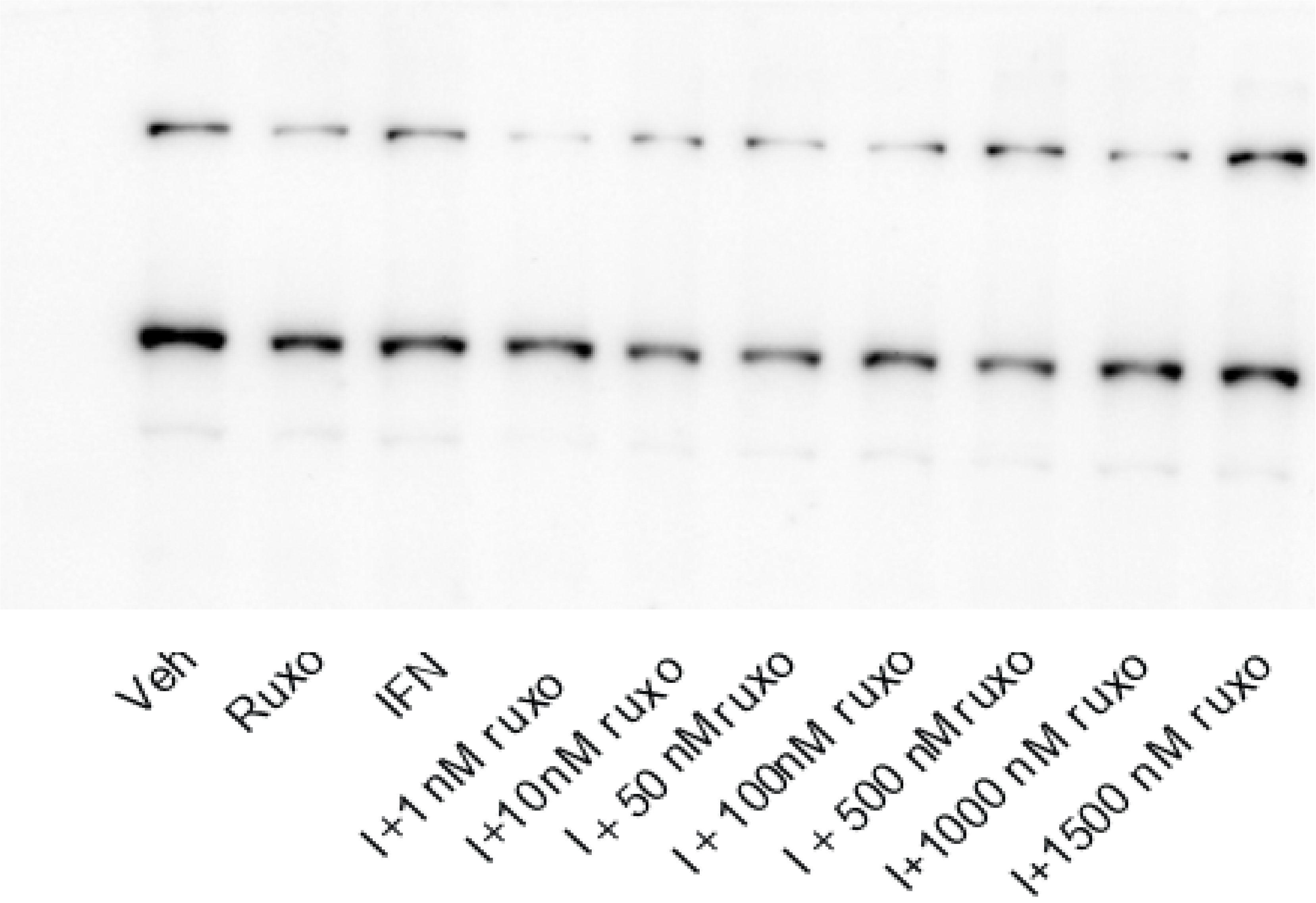

S1 Fig 1E, pJAK1

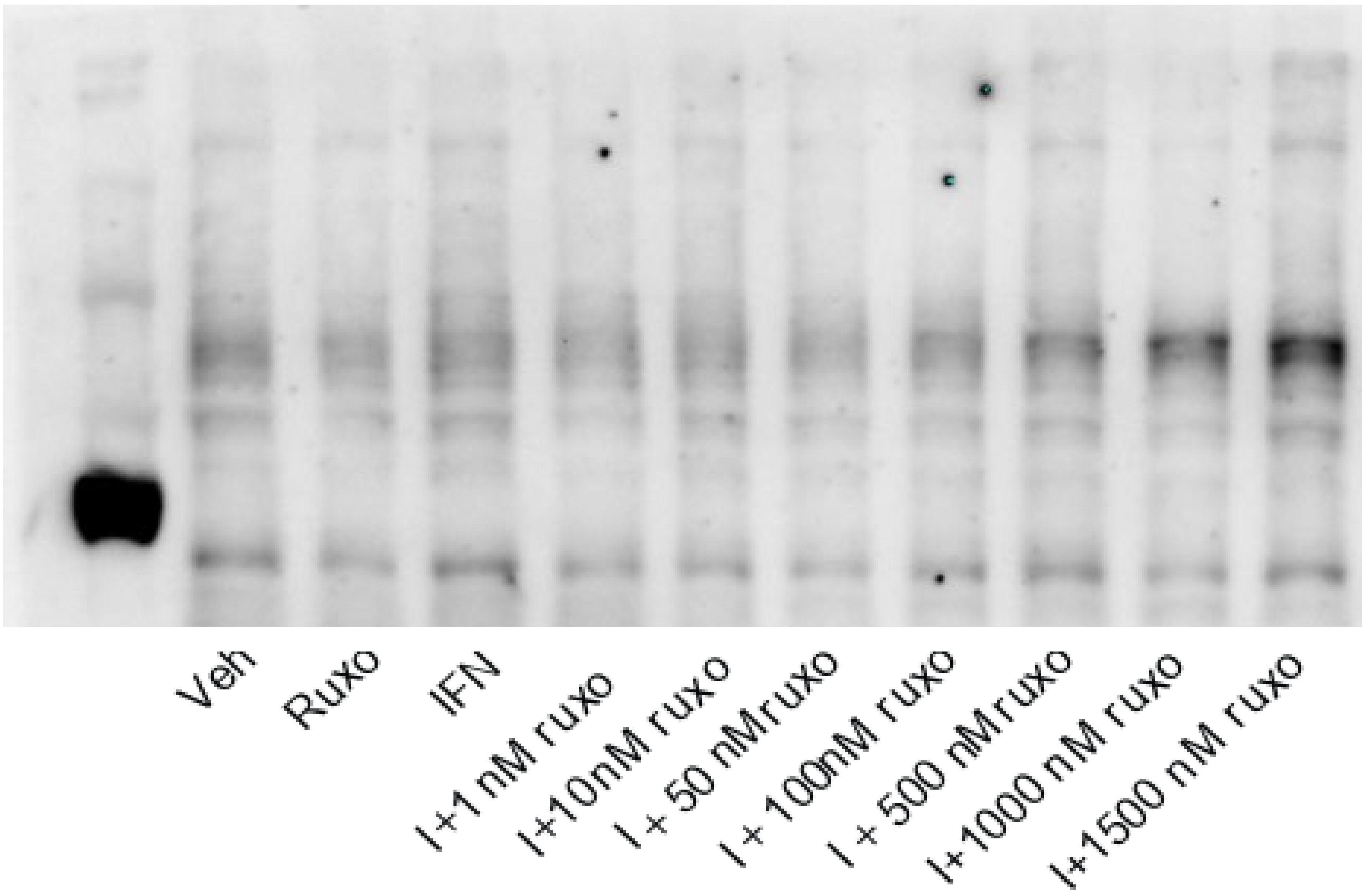

S1 Fig1E, GAPDH

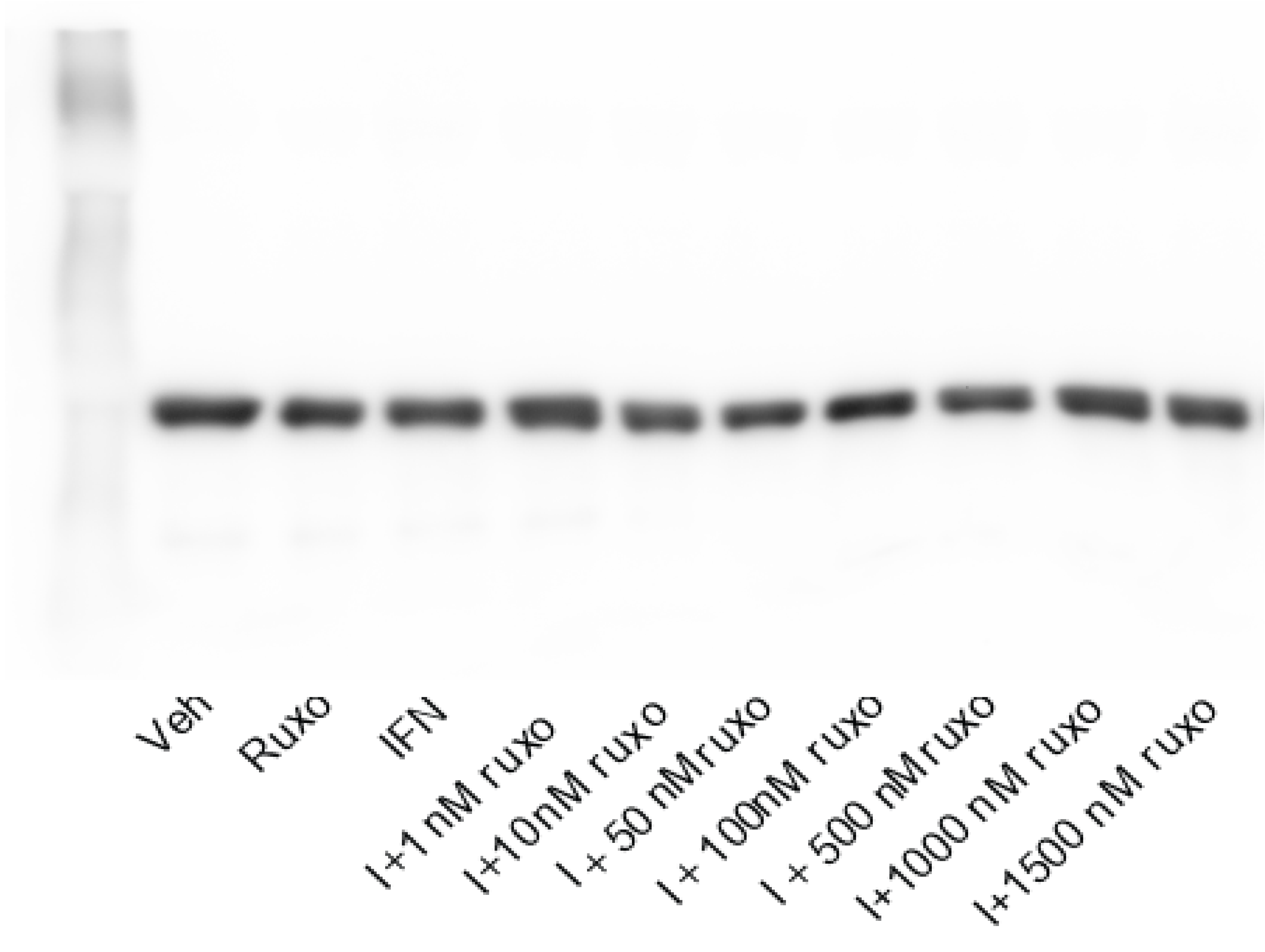

S1 Fig 1F, tJAK2

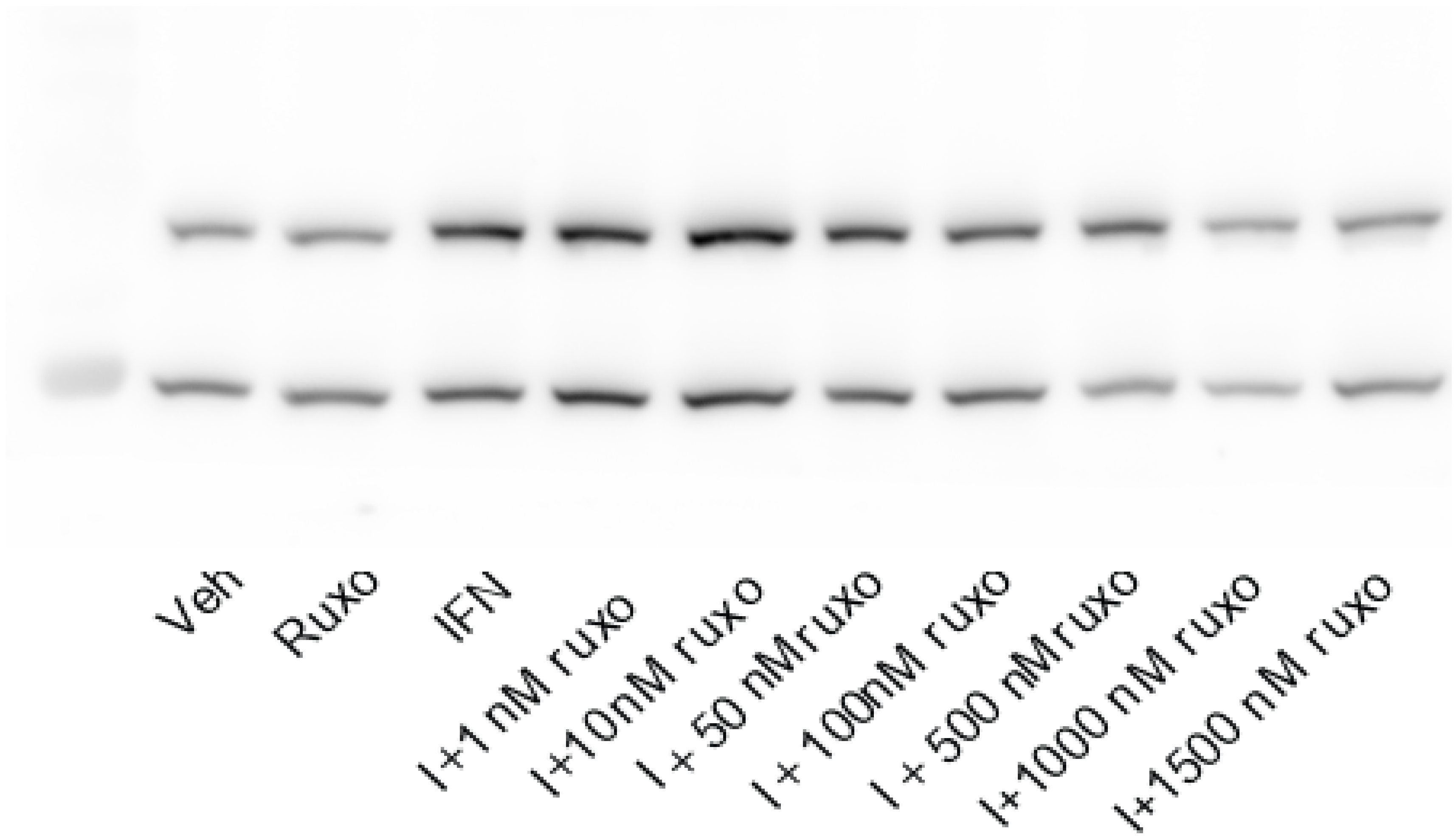

S1 Fig 1F, pJAK2

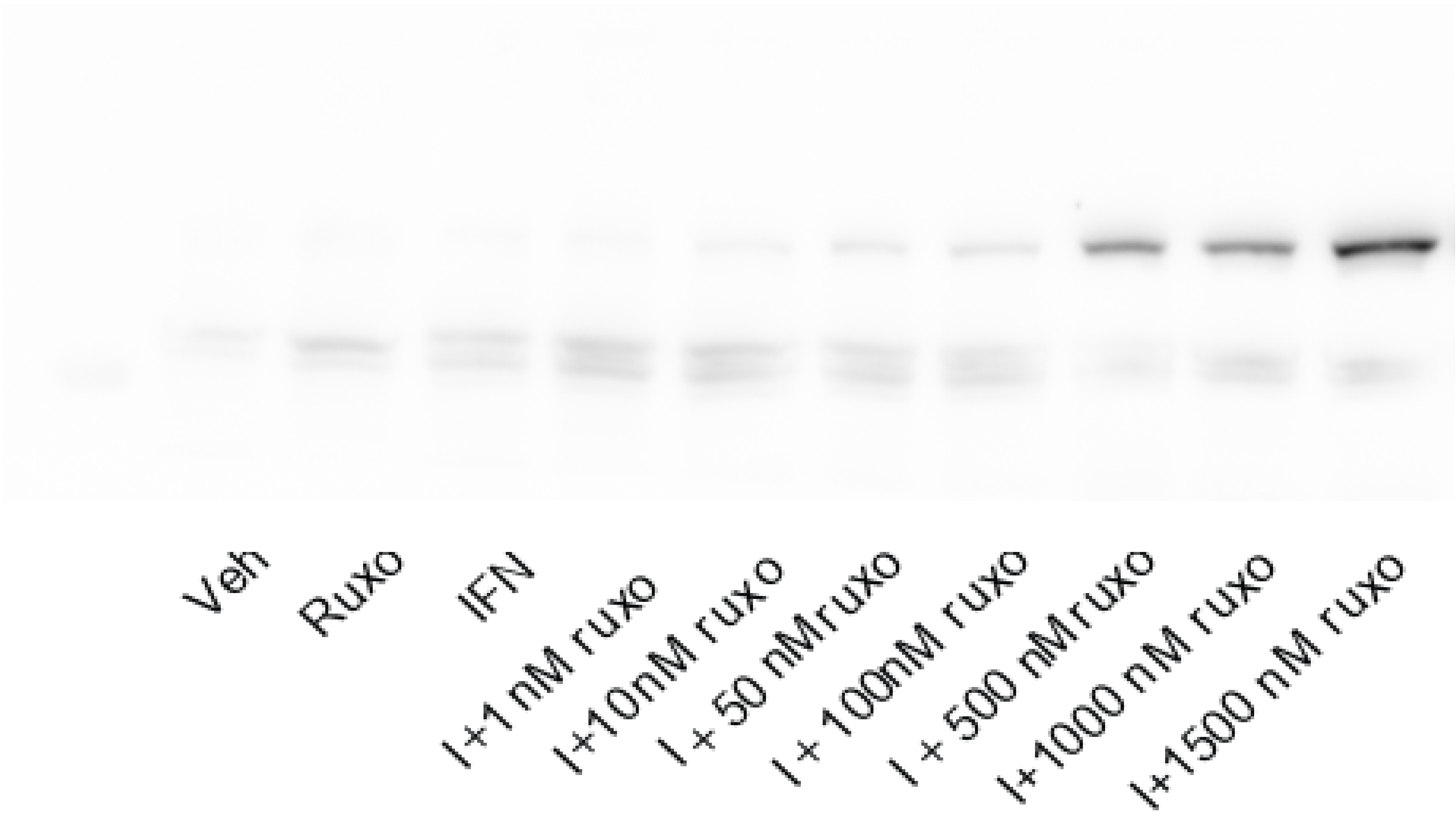

Figure 1F, GAPDH

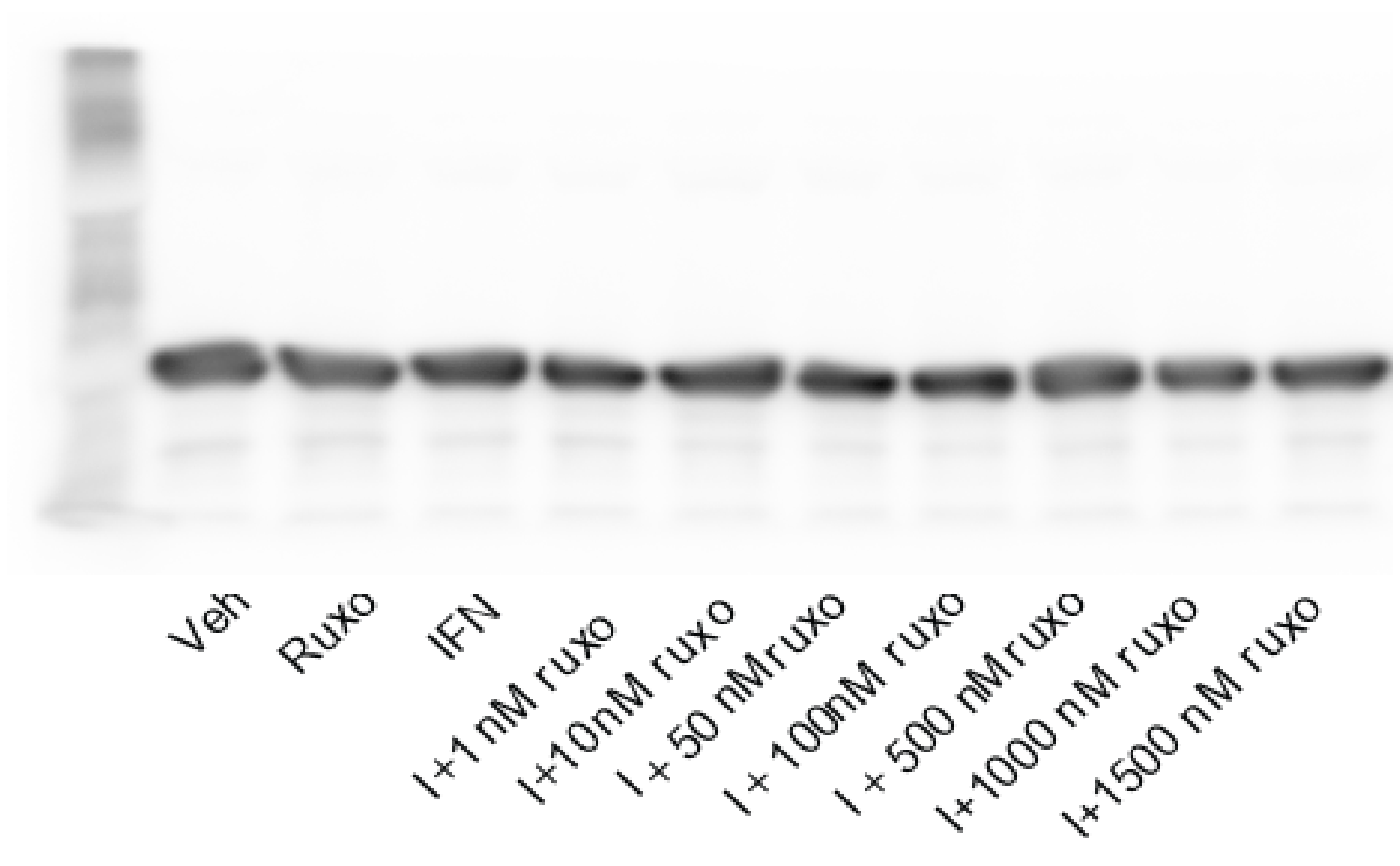

S1 Fig 1G, tSTAT1

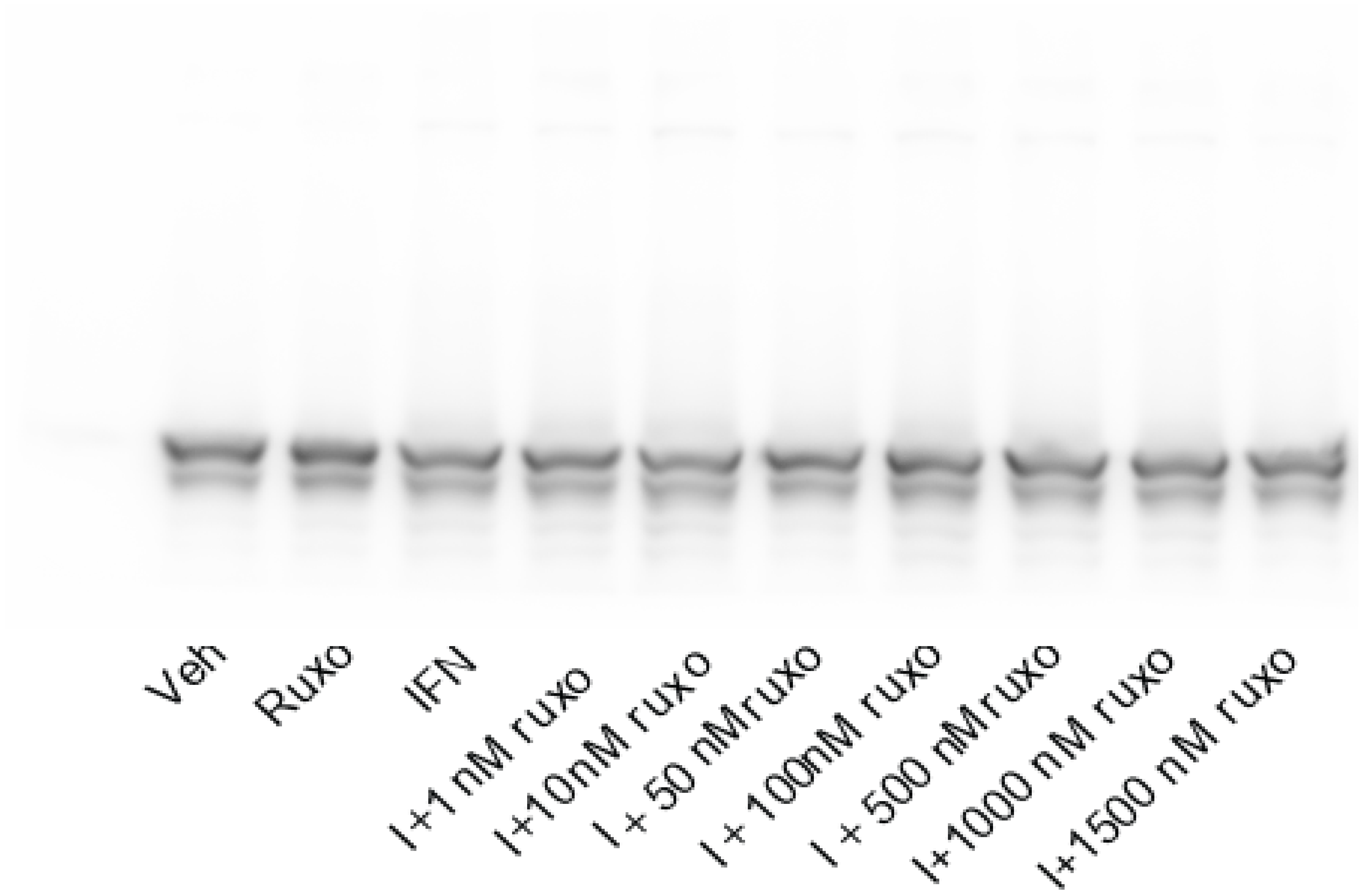

S1 Fig 1G, pSTAT1

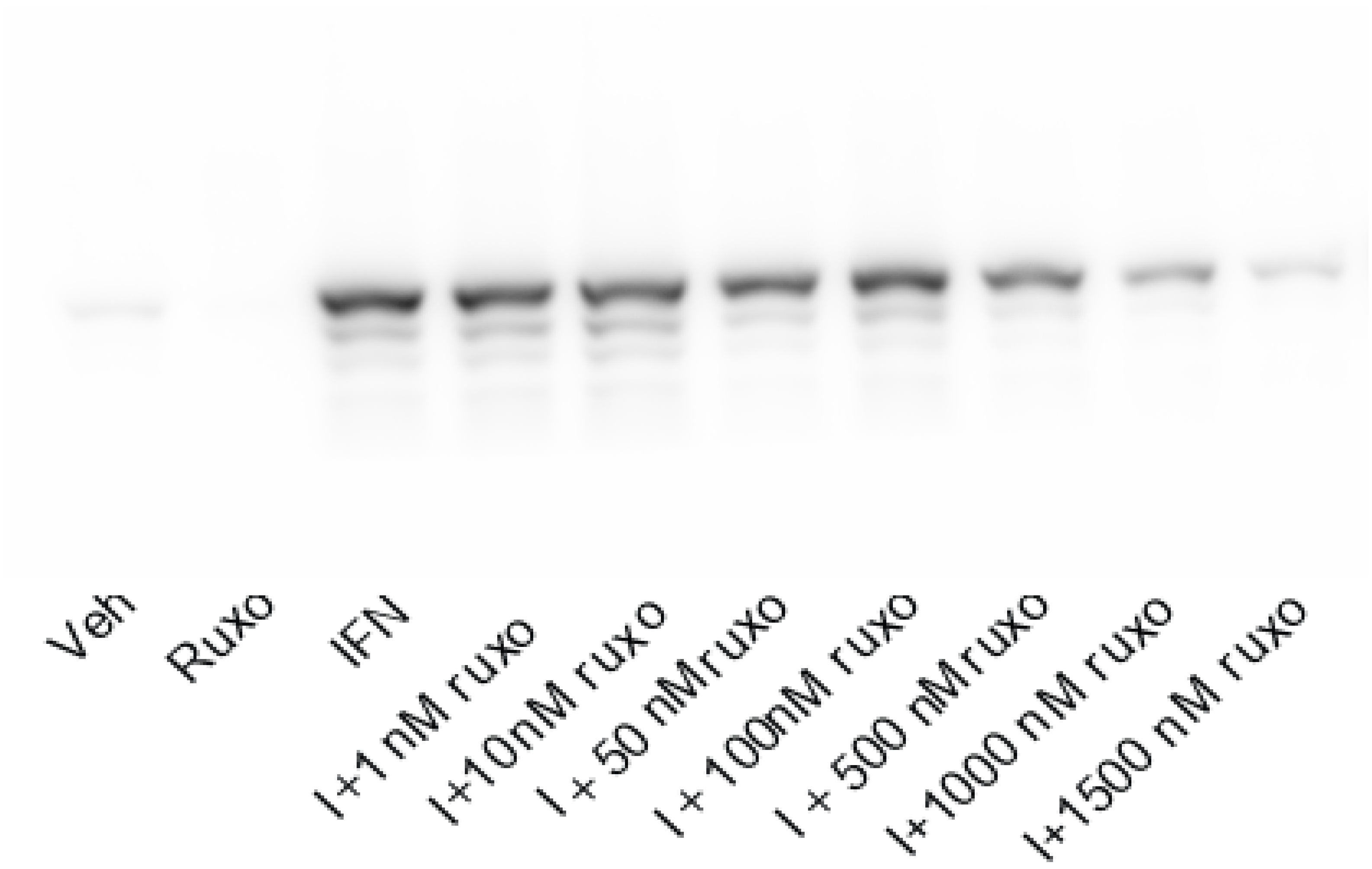

S1 Fig 1G, GAPDH

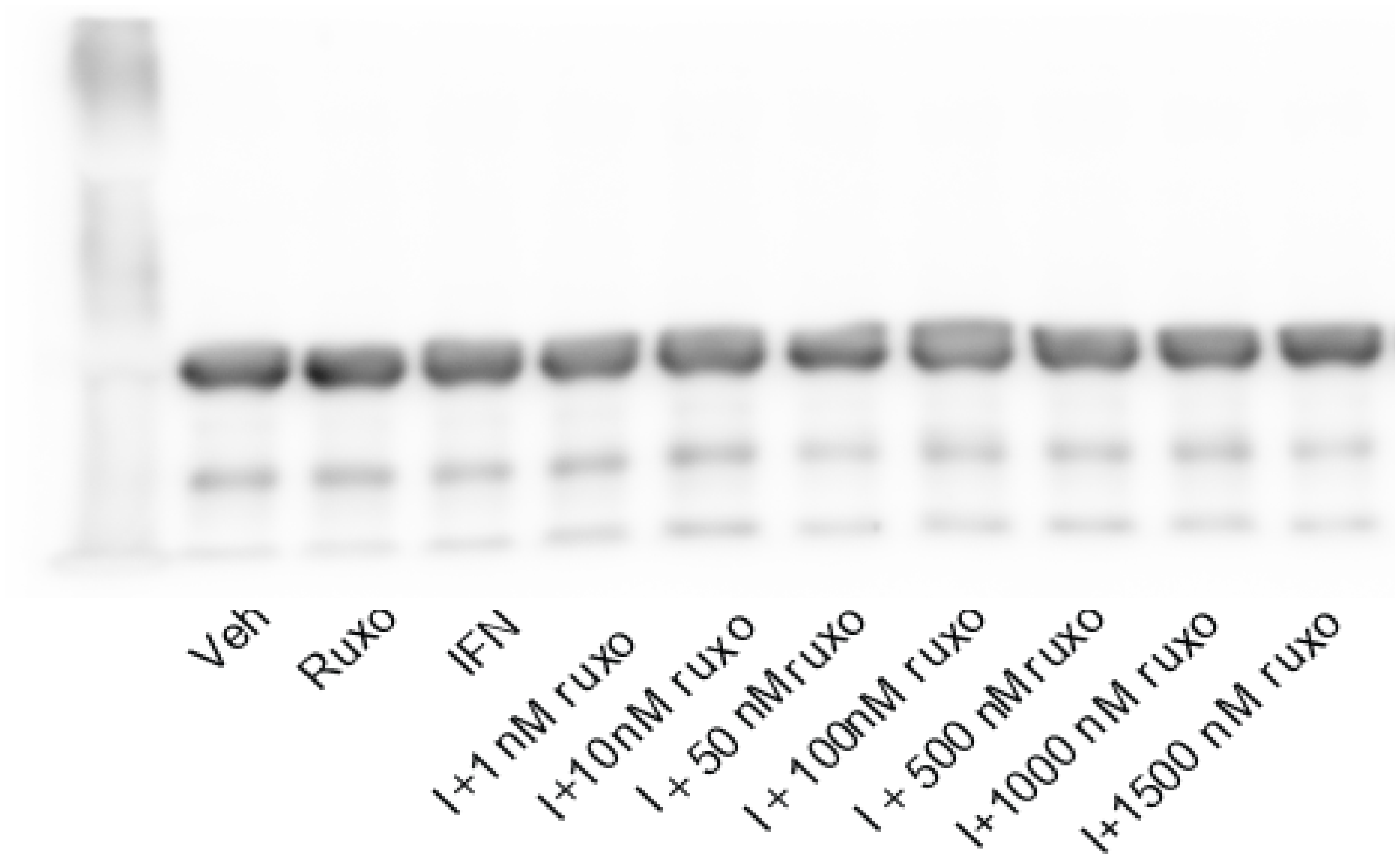

S1 Fig 2A, pJAK2

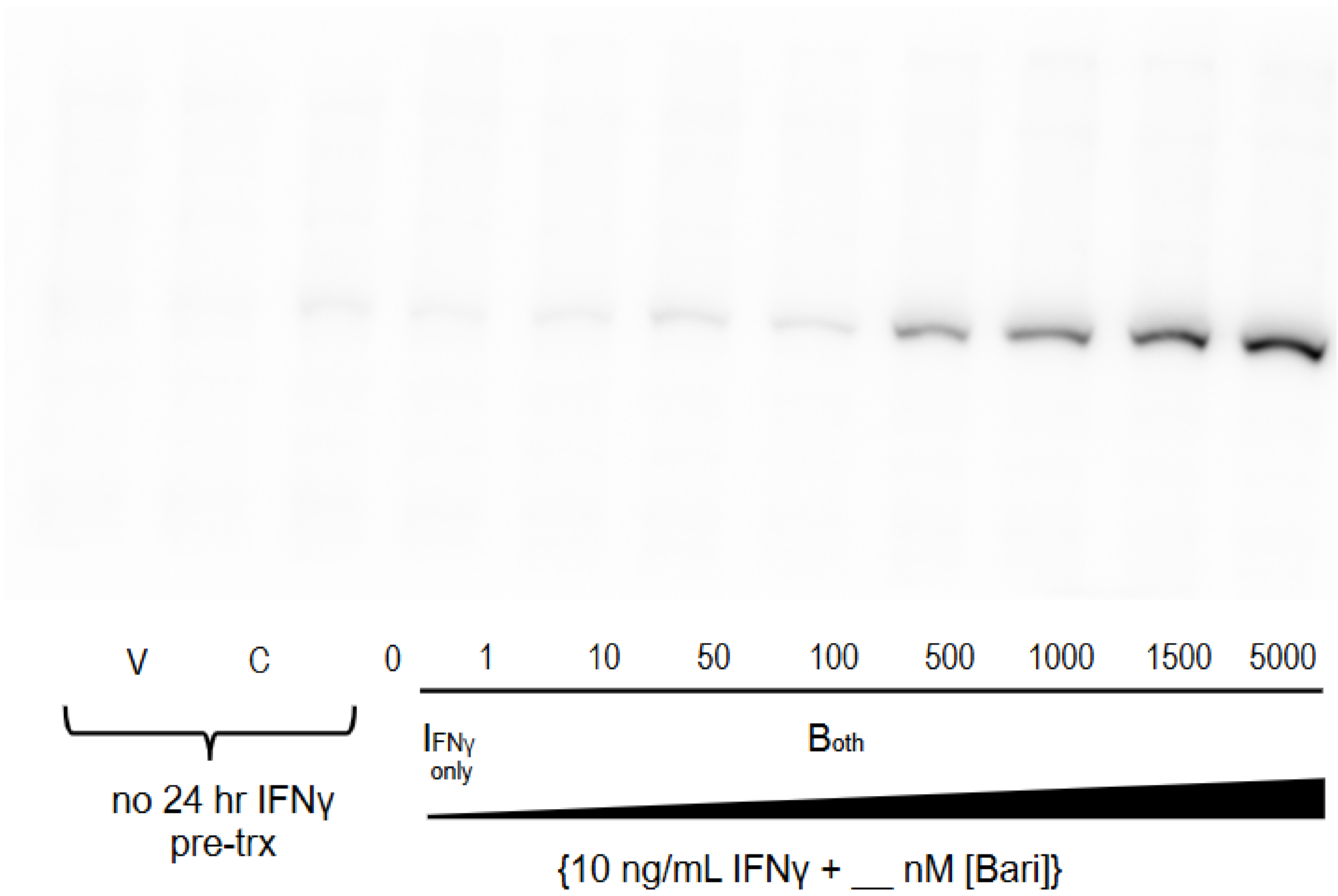

S1 Fig 2 A, tJAK2

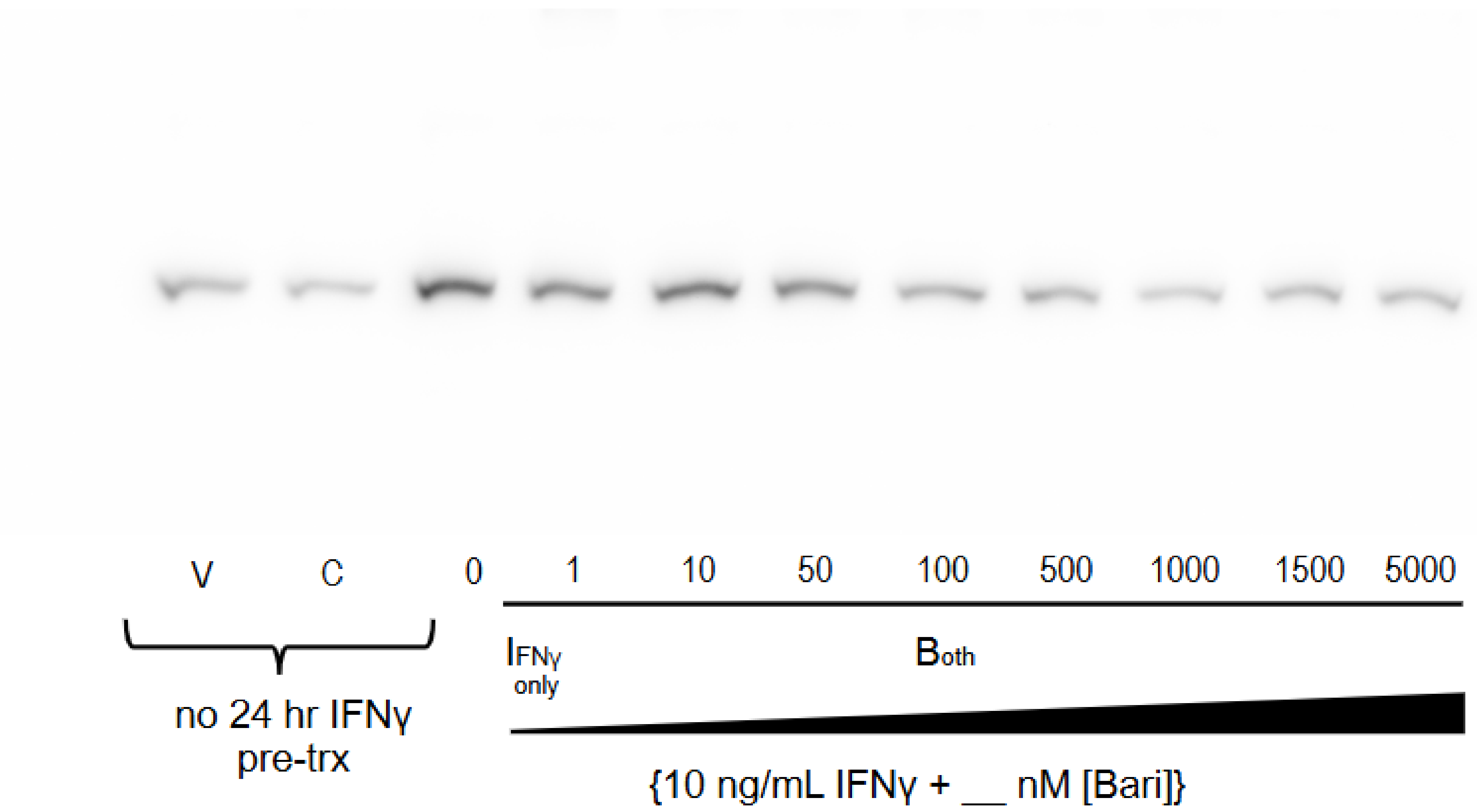

S1 Fig 2 A, GAPDH

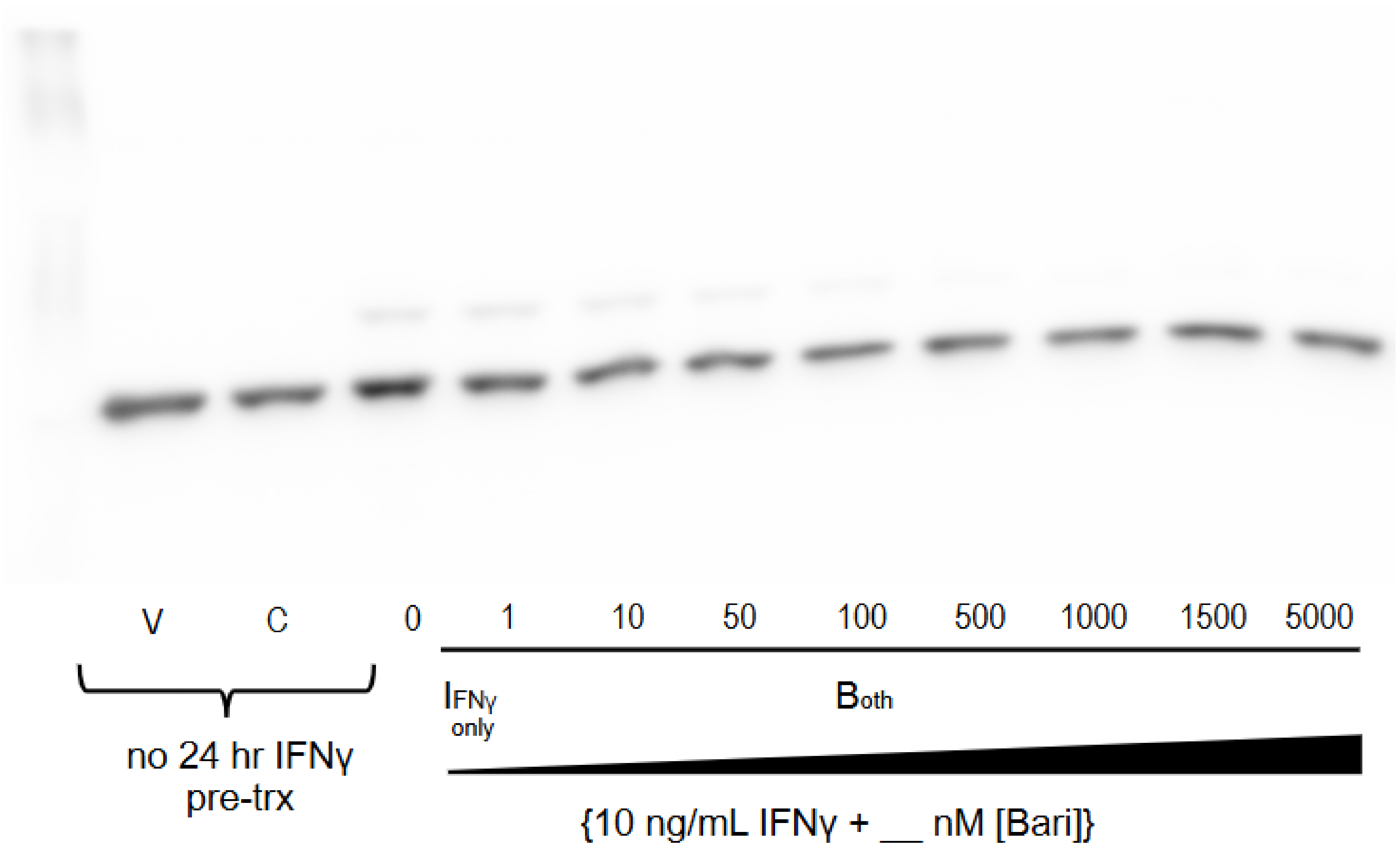

S1 Fig 2 B, pJAK2

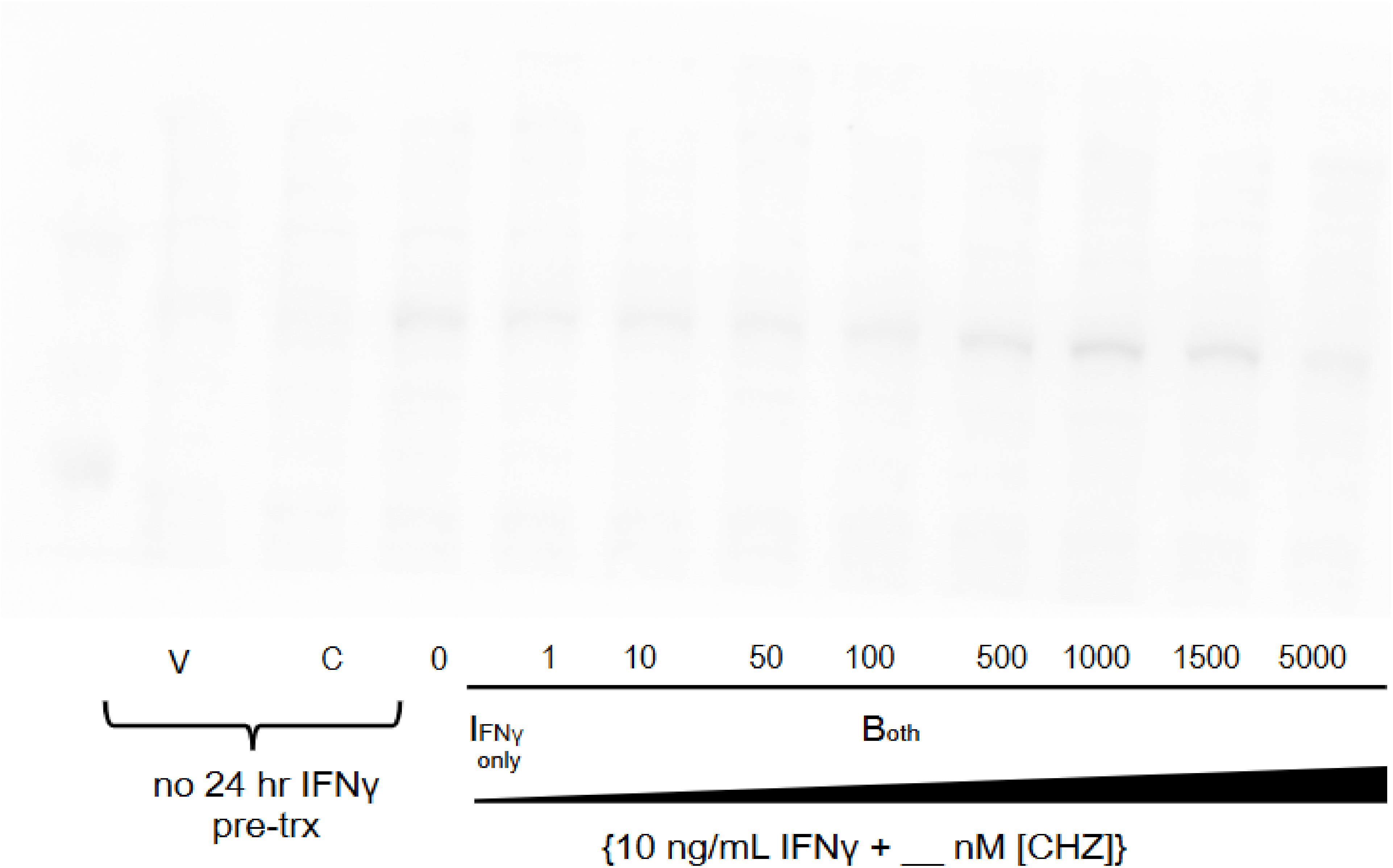

S1 Fig 2 B, tJAK2

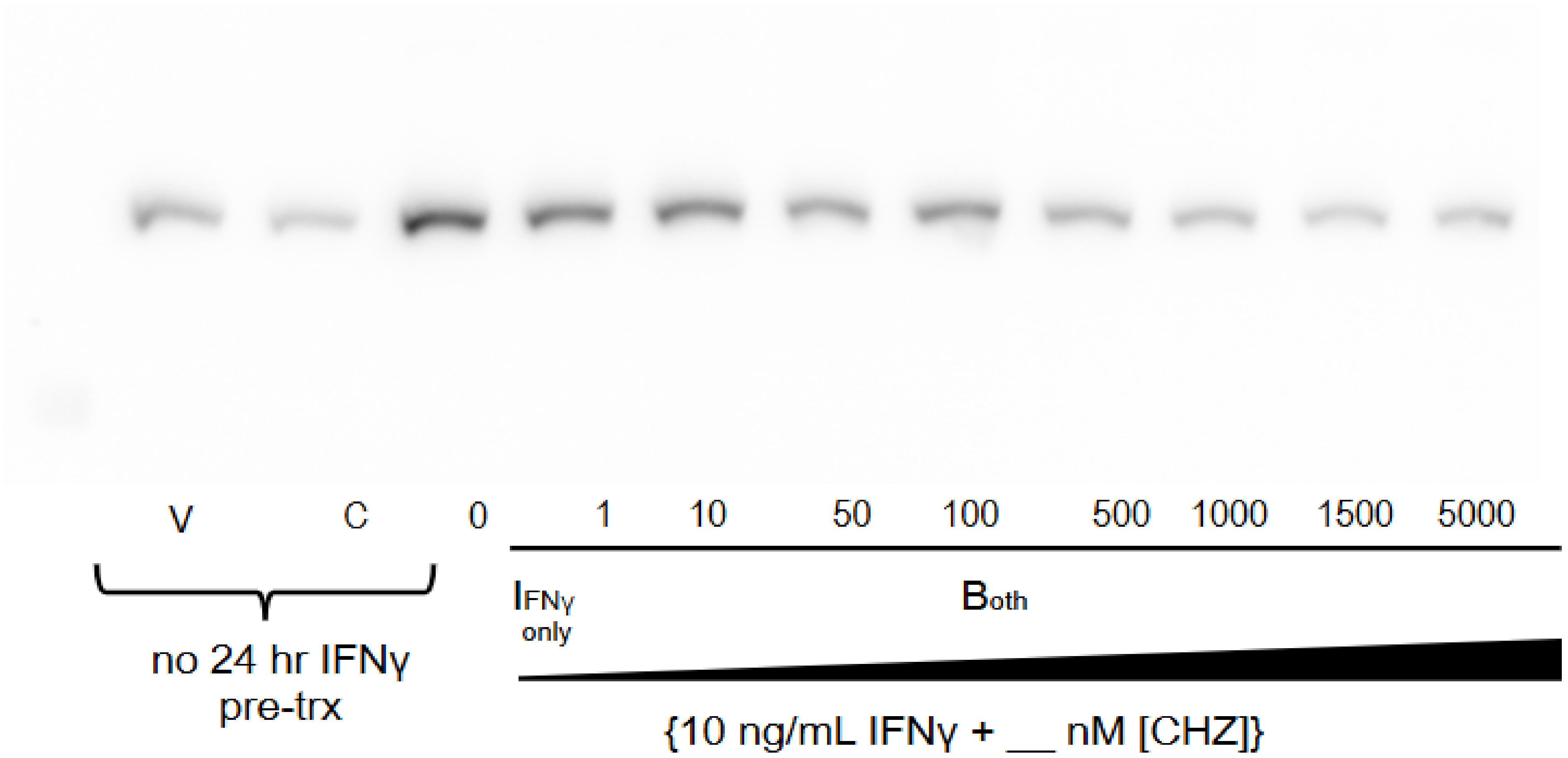

S1 Fig 2 B, GAPDH

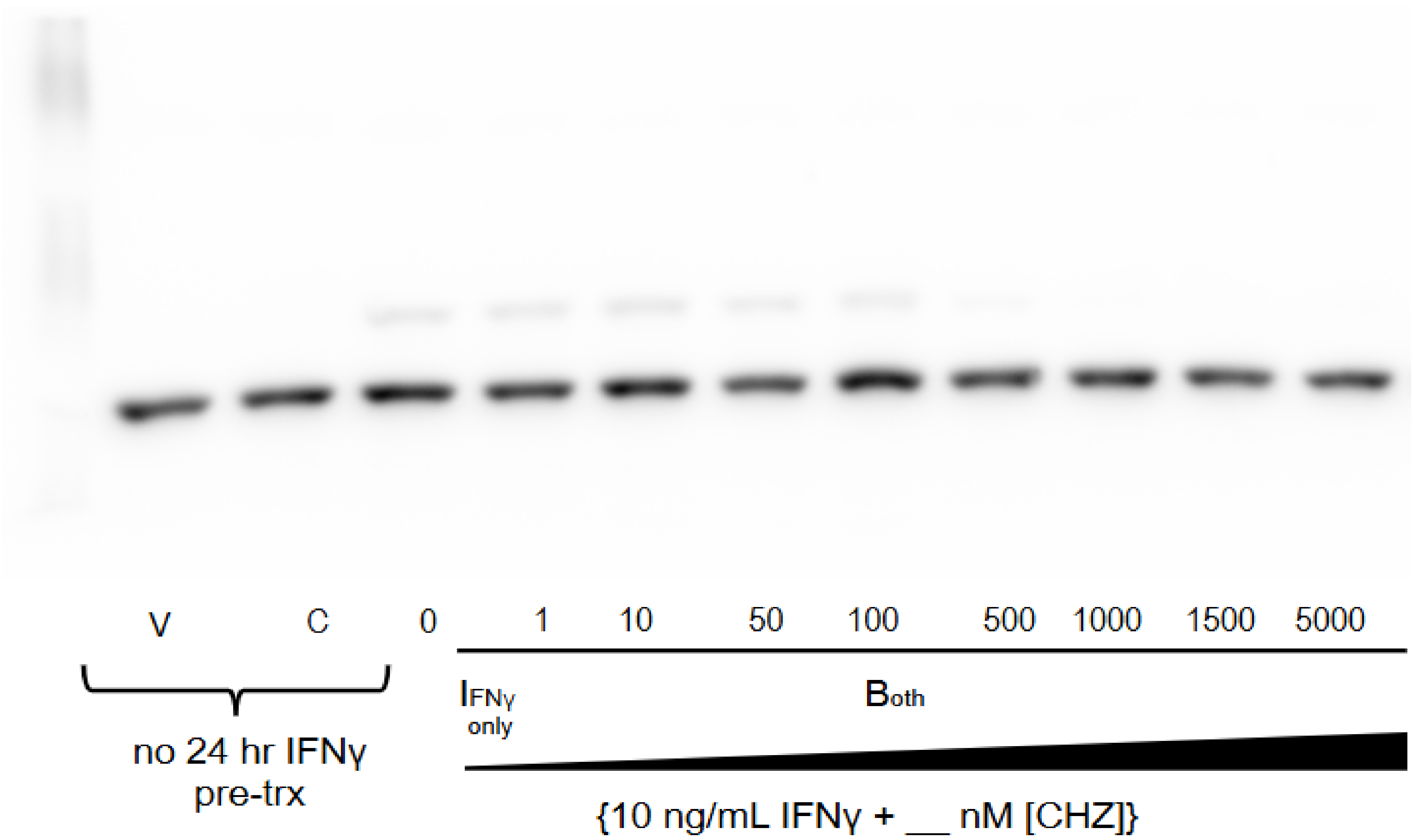

S1 Fig 2 C, pSTAT1

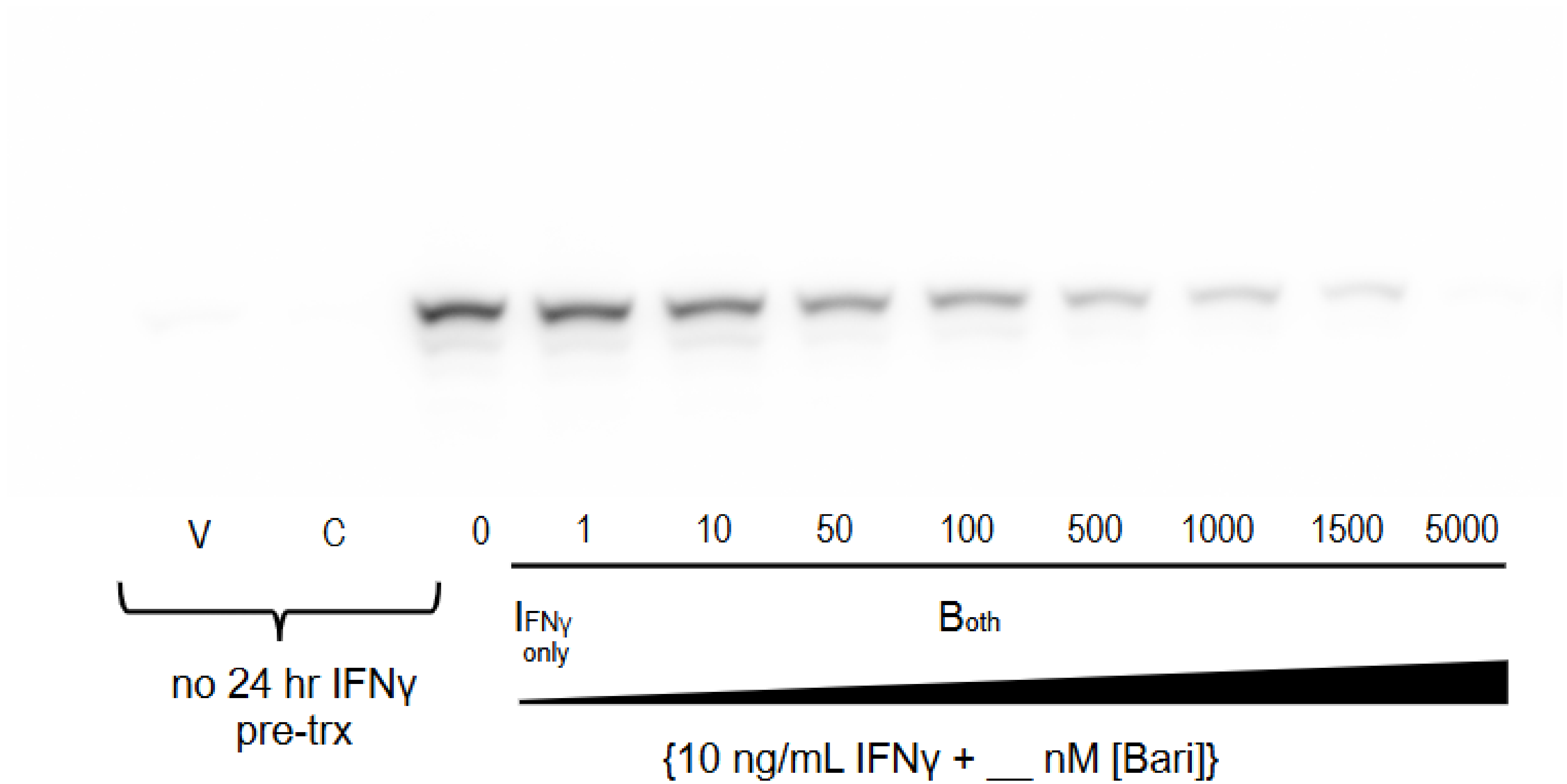

S1 Fig 2 C, tSTAT1

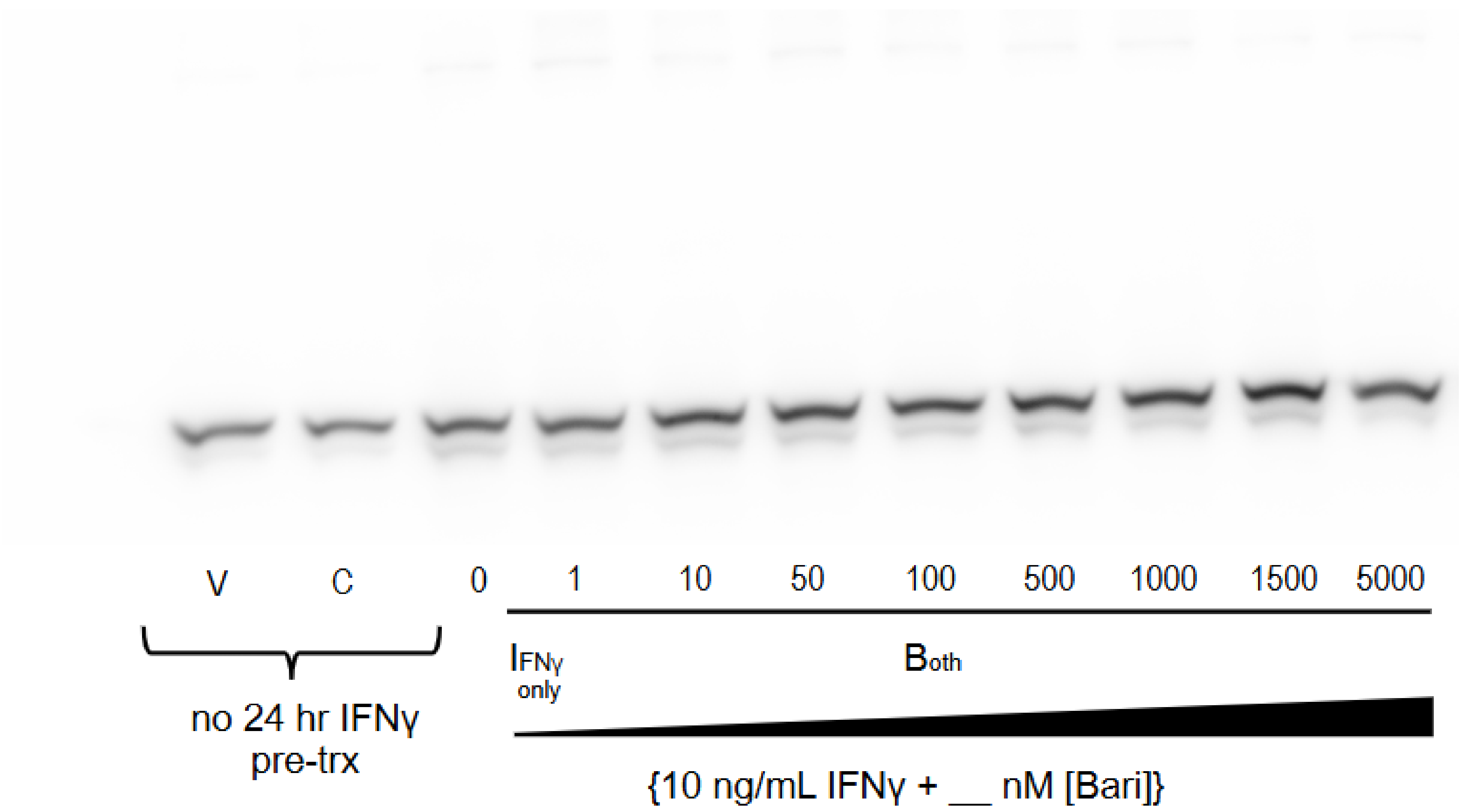

S1 Fig 2 C, GAPDH

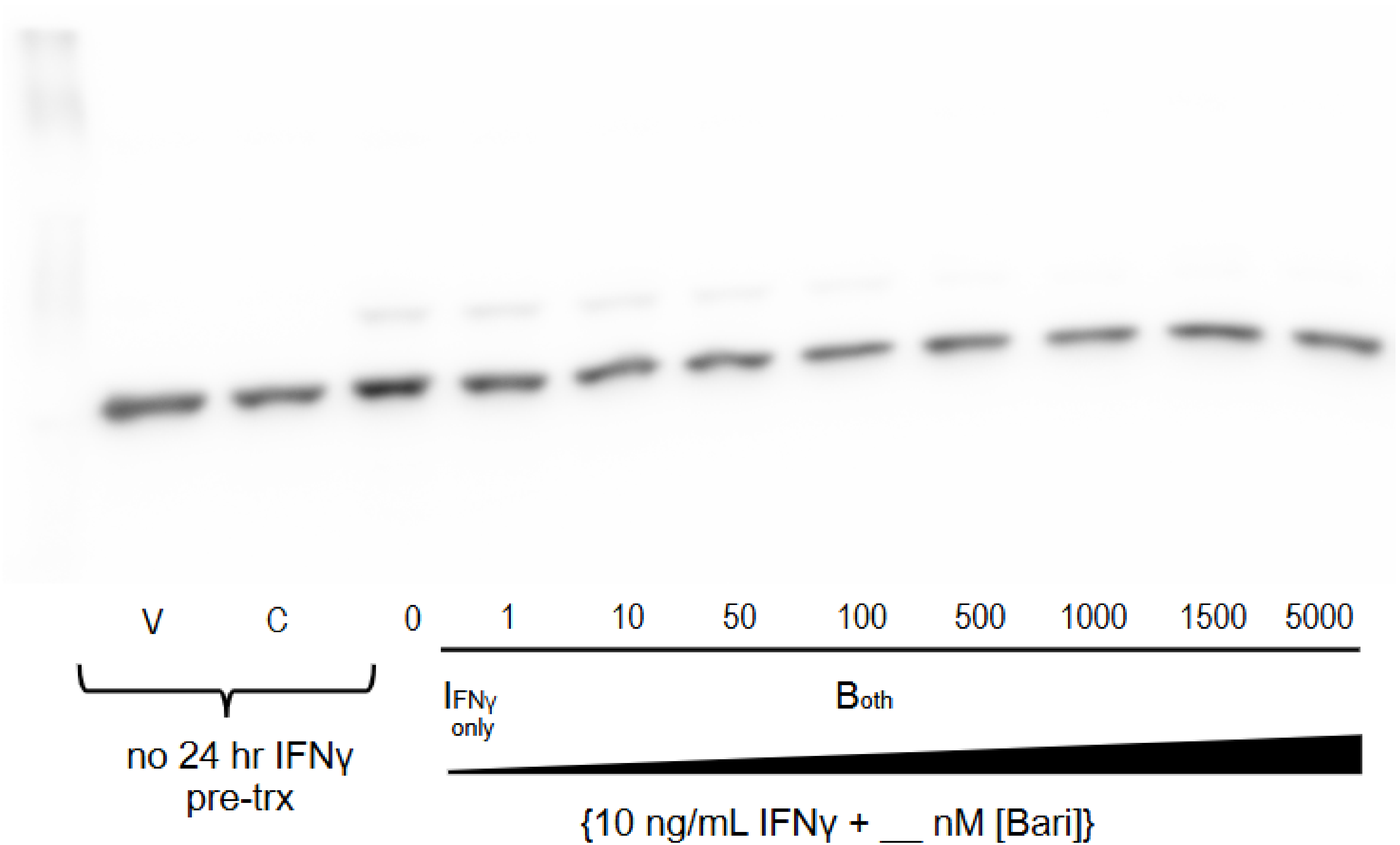

S1 Fig 2 D, pSTAT1

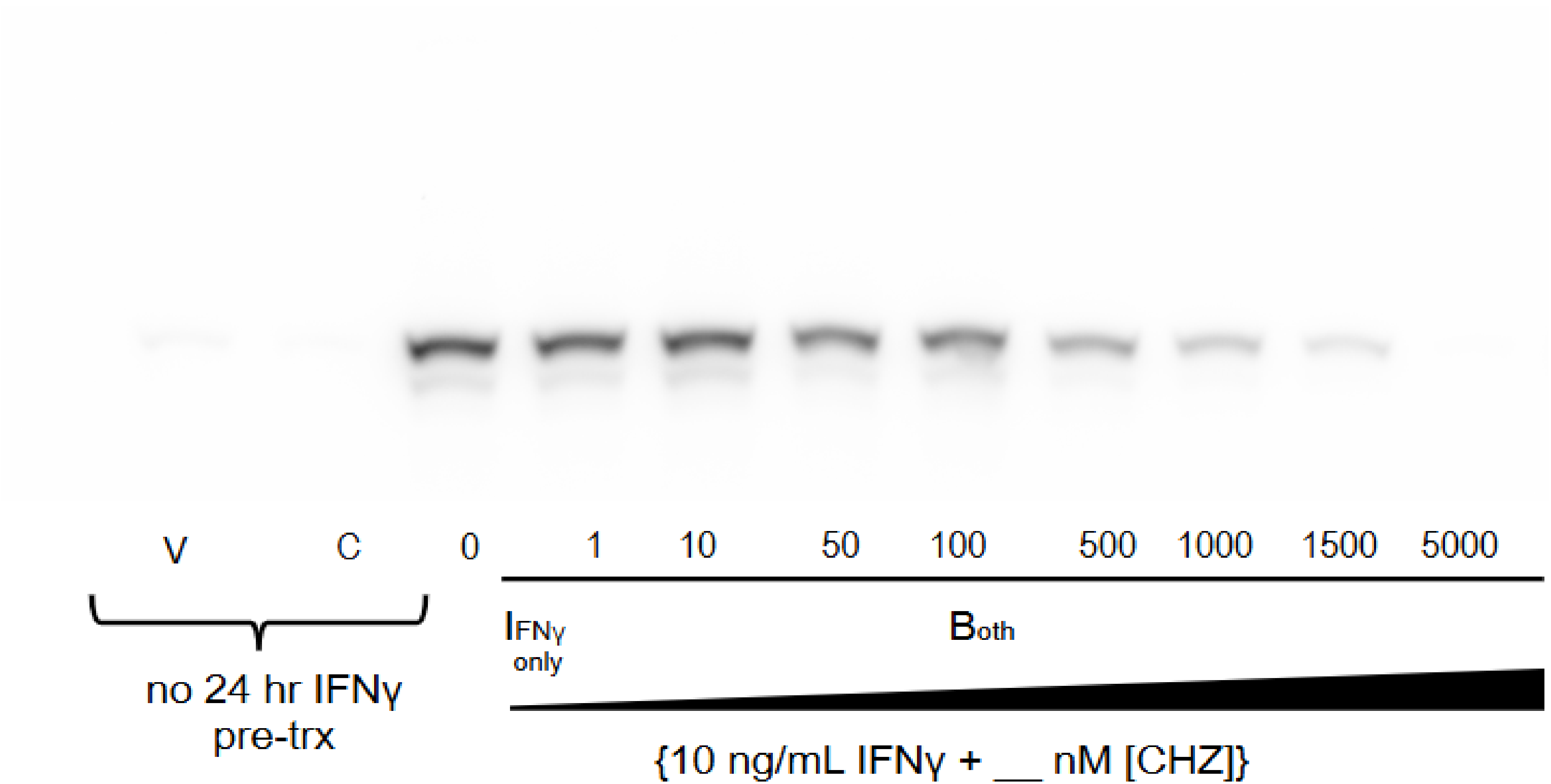

S1 Fig 2 D, tSTAT1

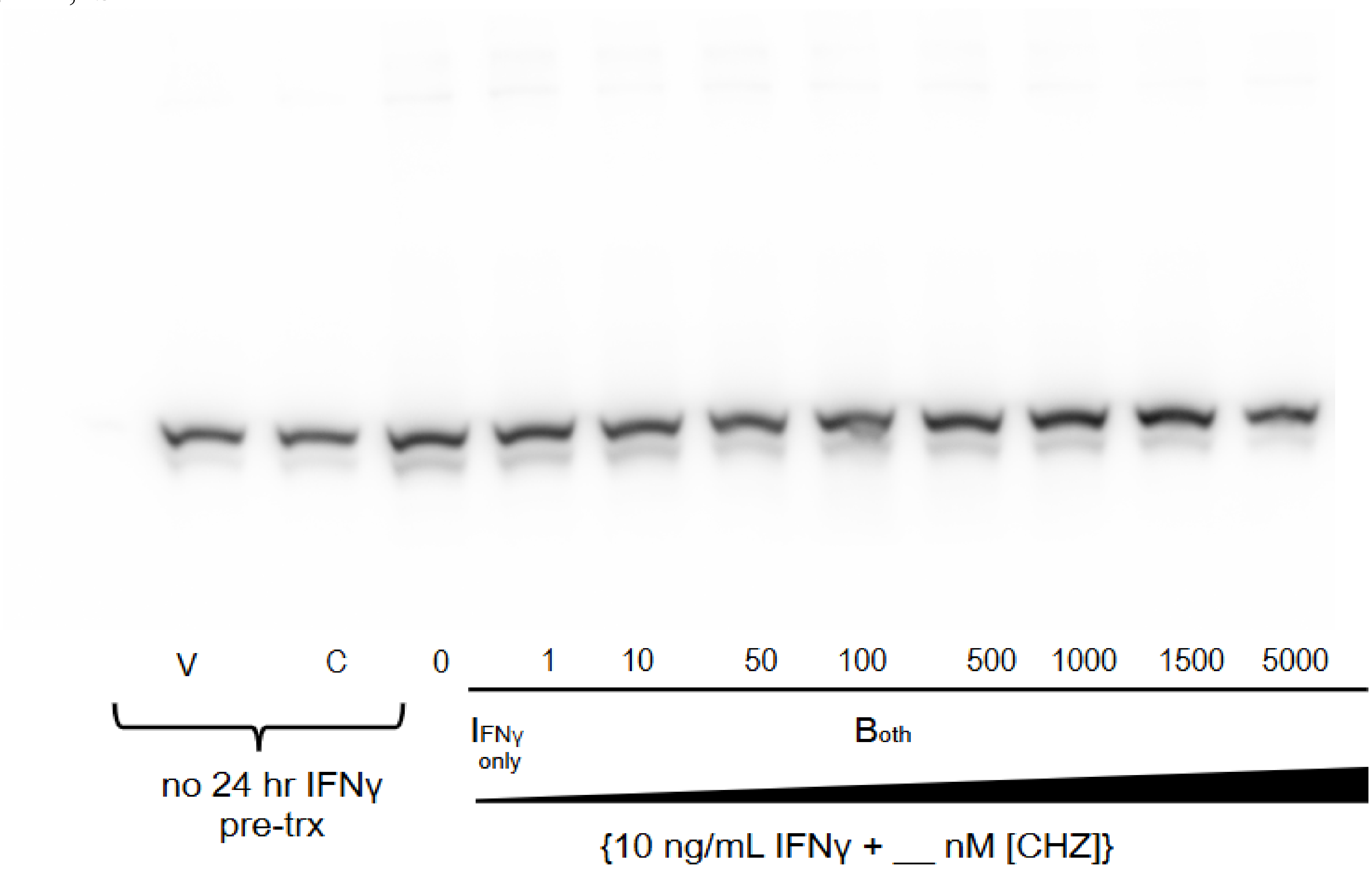

S1 Fig 2 D, GAPDH

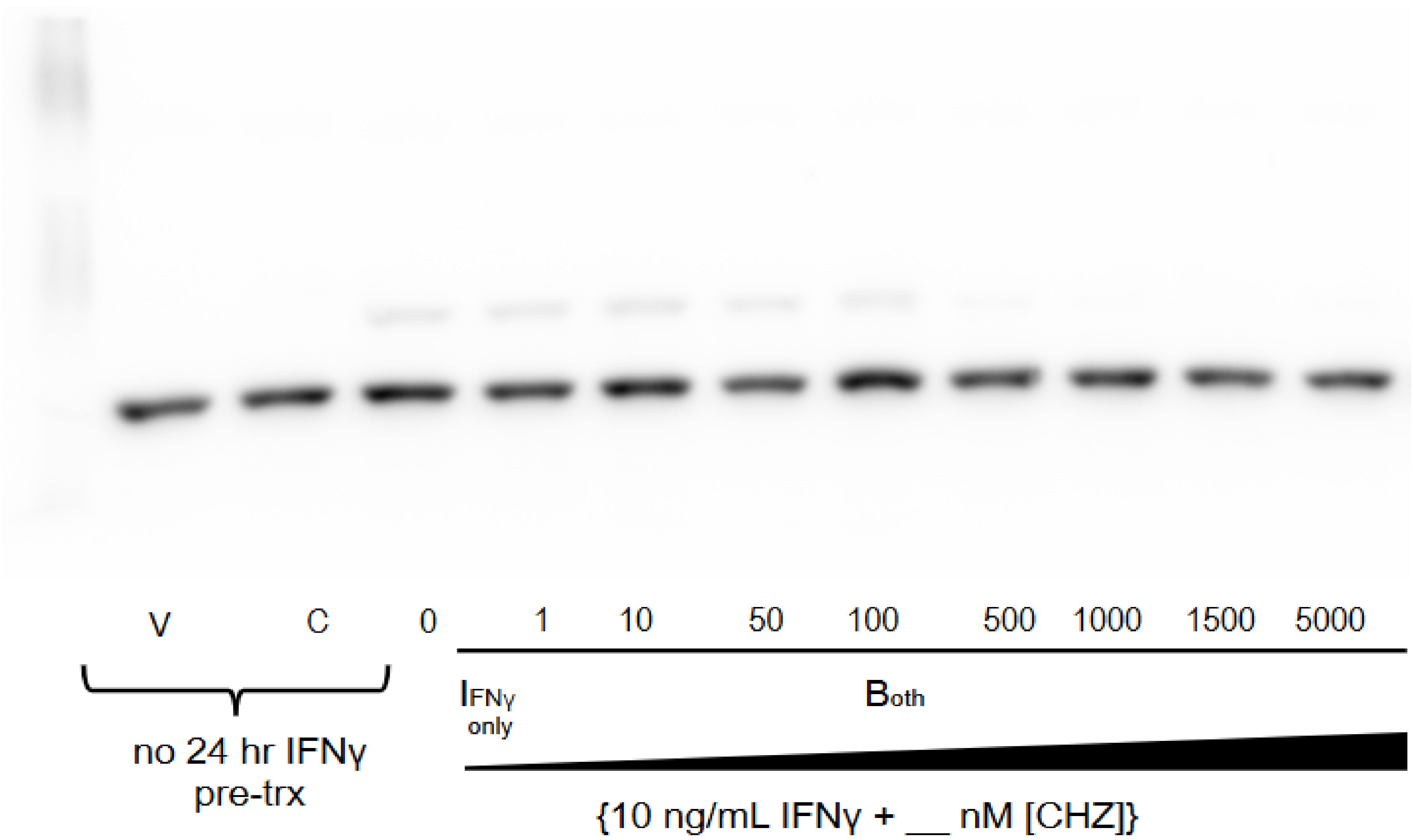

S1 Fig 3 A, tJAK2

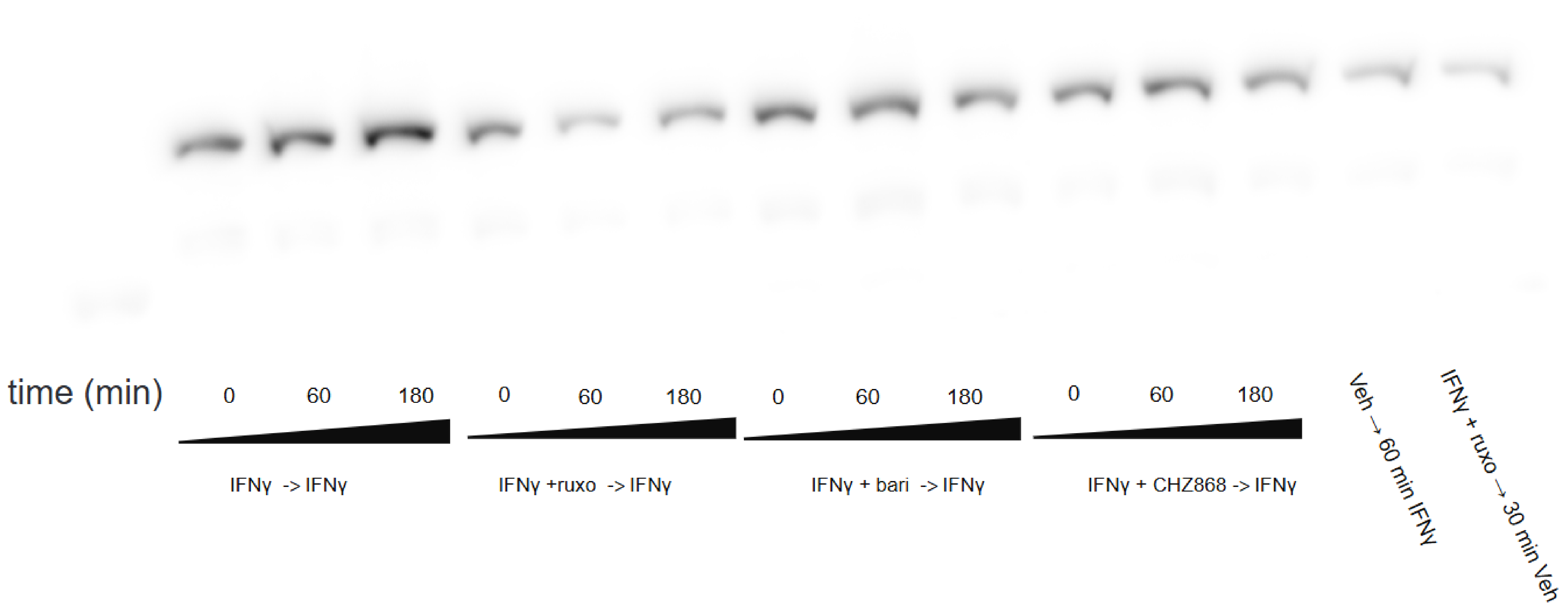

S1 Fig 3 A, pJAK2

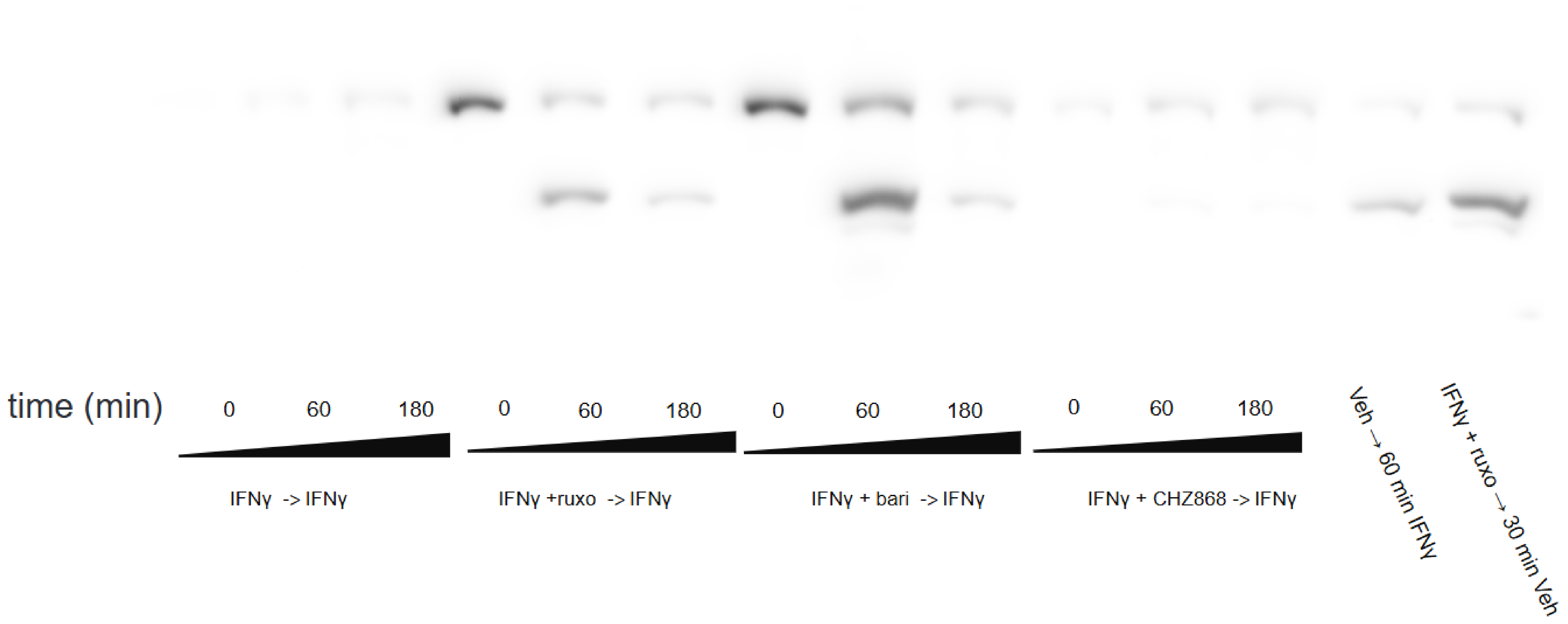

S1 Fig 3 A, GAPDH

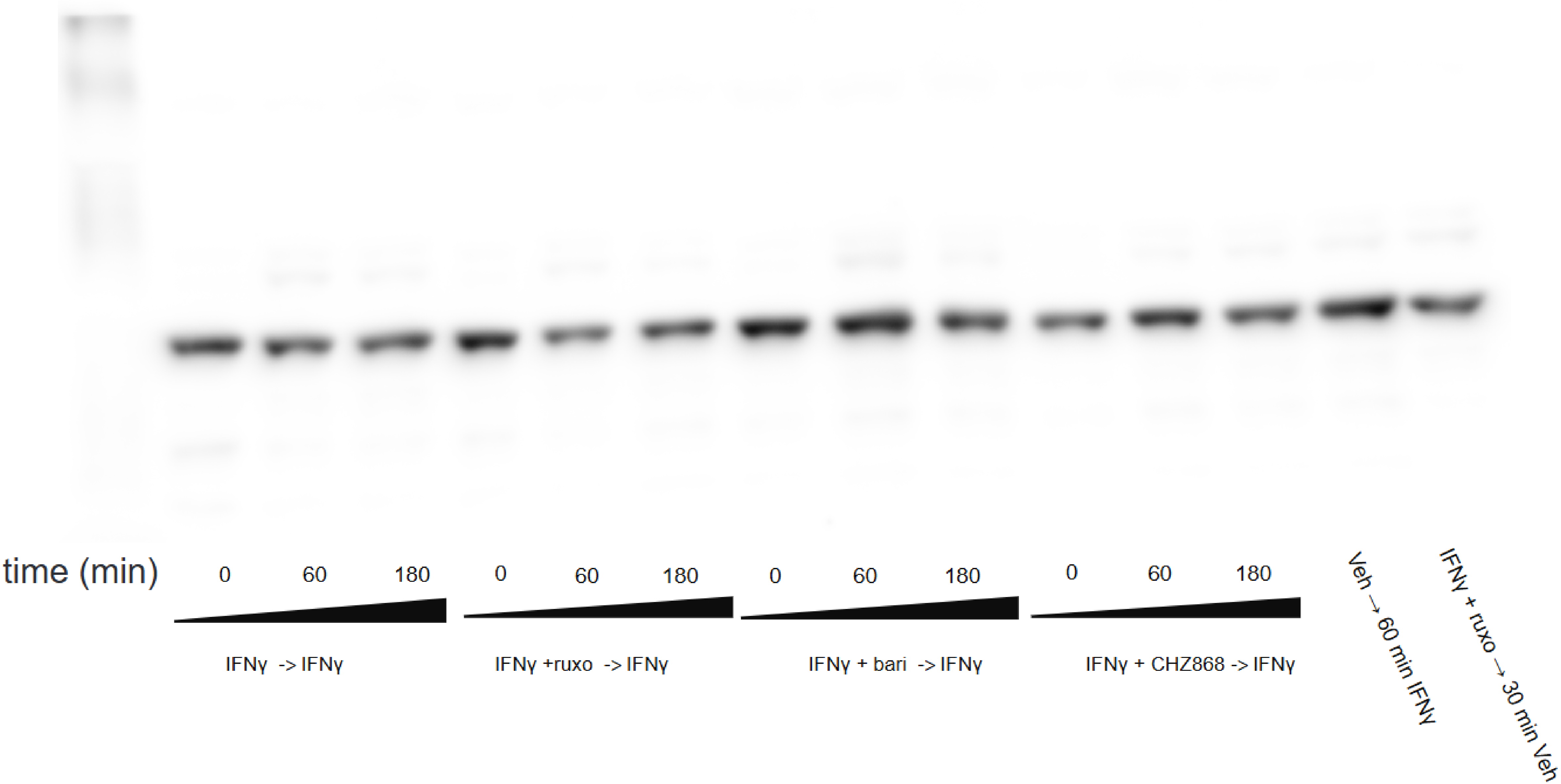

S1 Fig 3 B, tSTAT1

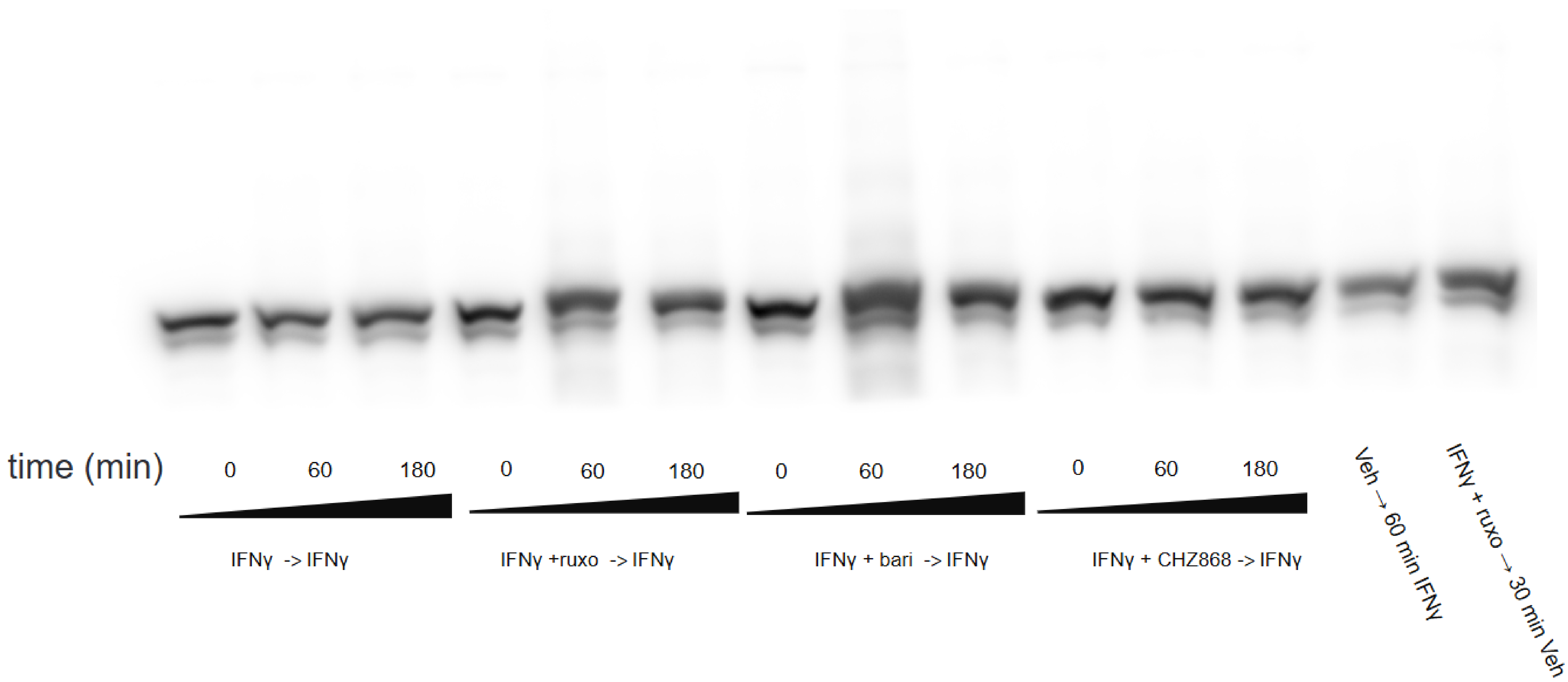

S1 Fig 3 B, pSTAT1

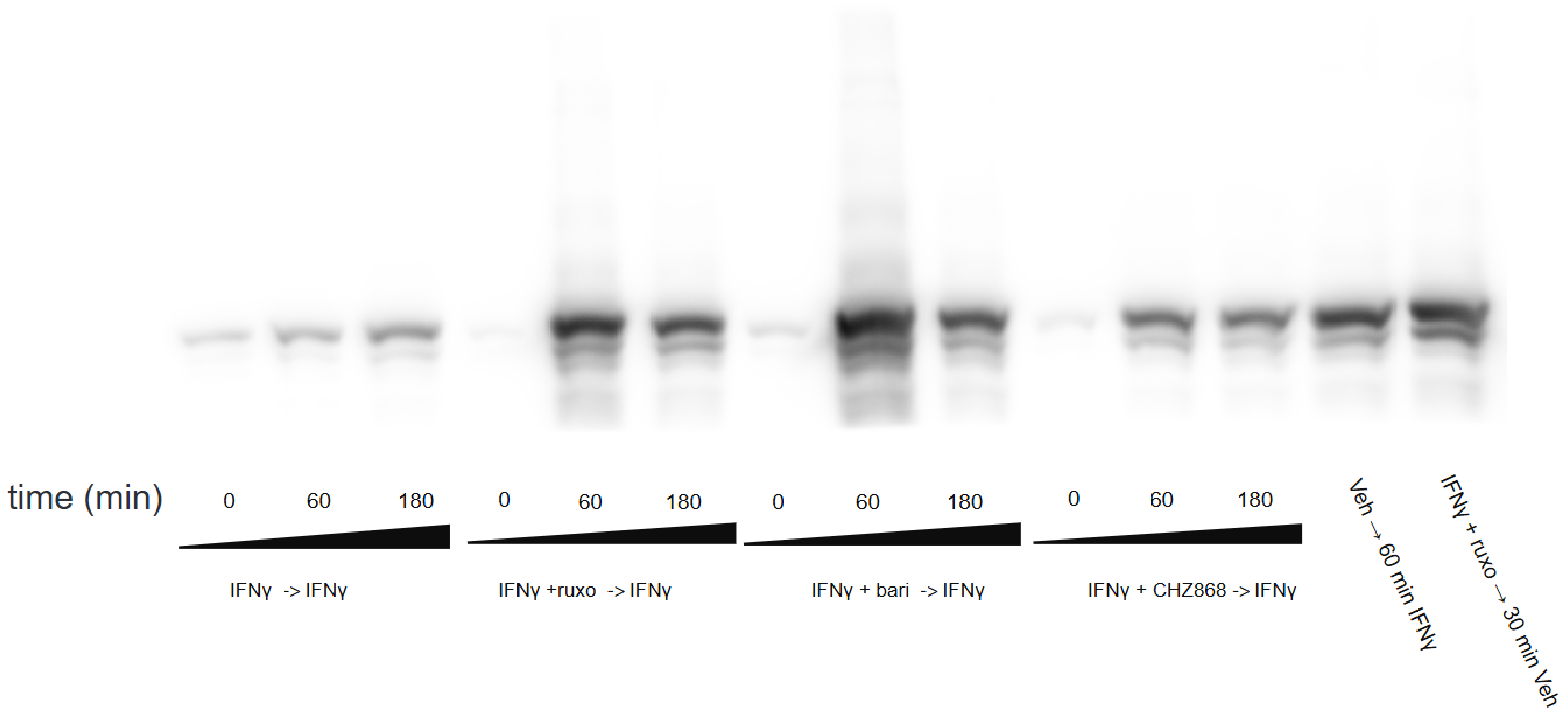

S1 Fig 3 B, GAPDH

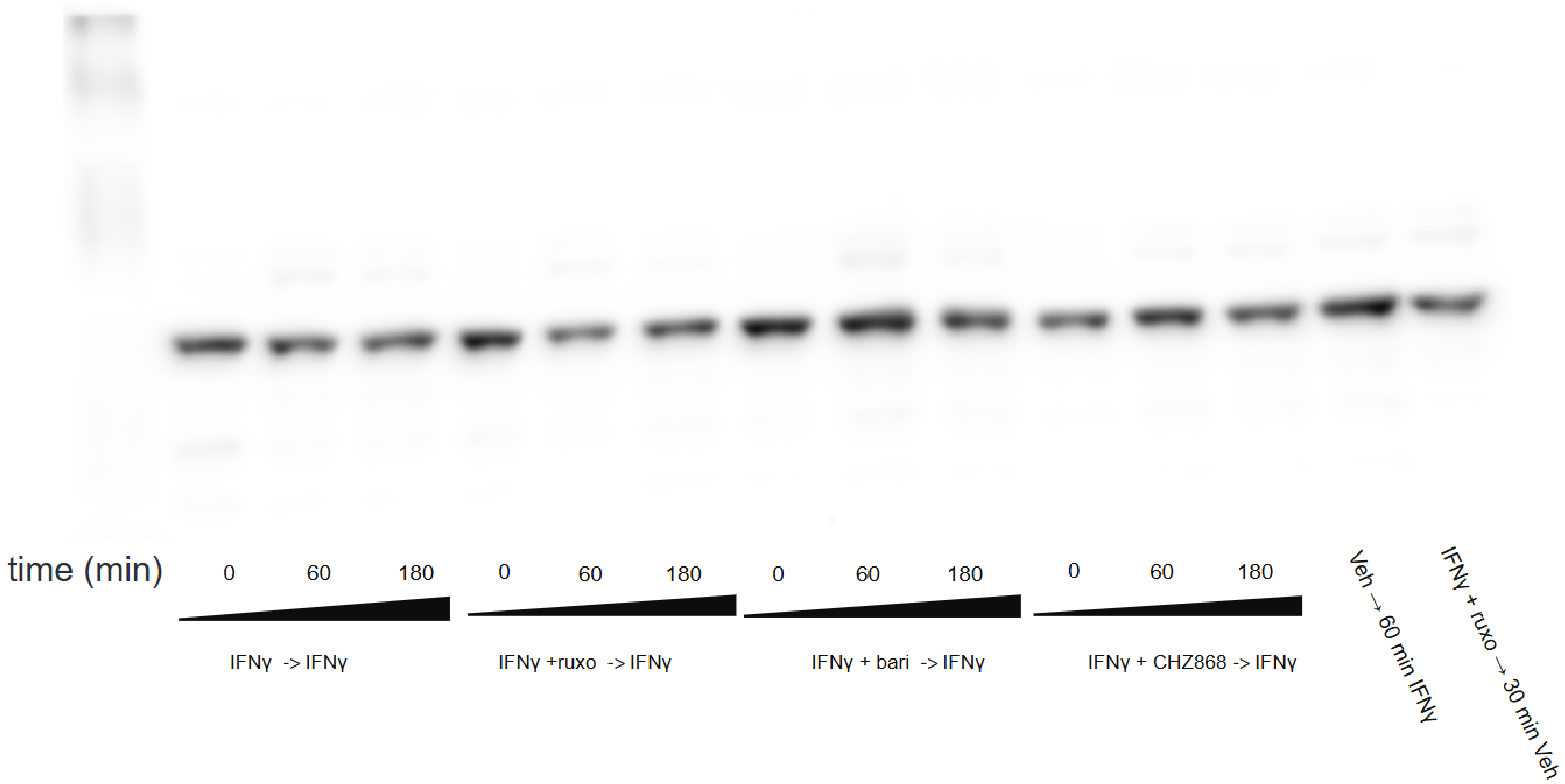

S1 Fig 3 C, tSTAT2

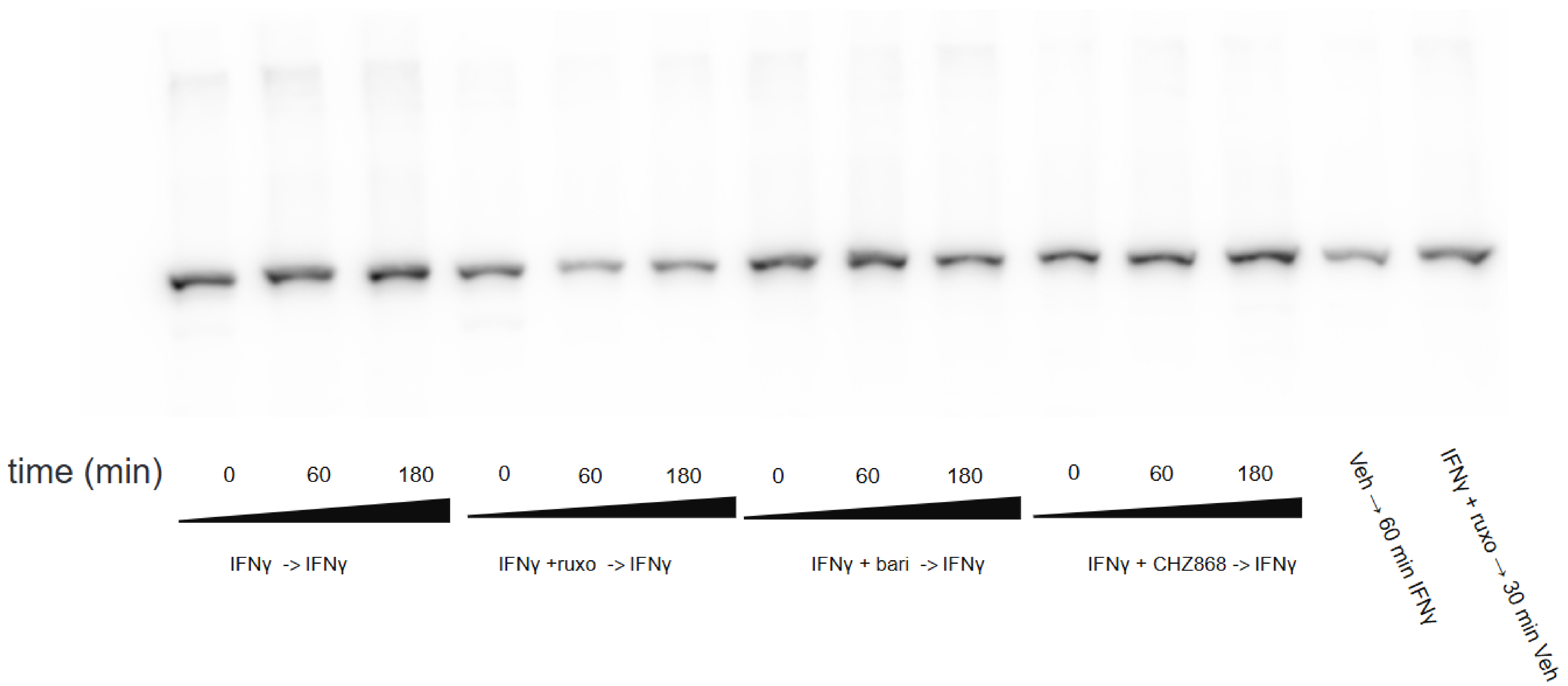

S1 Fig 3 C, GAPDH for tSTAT2

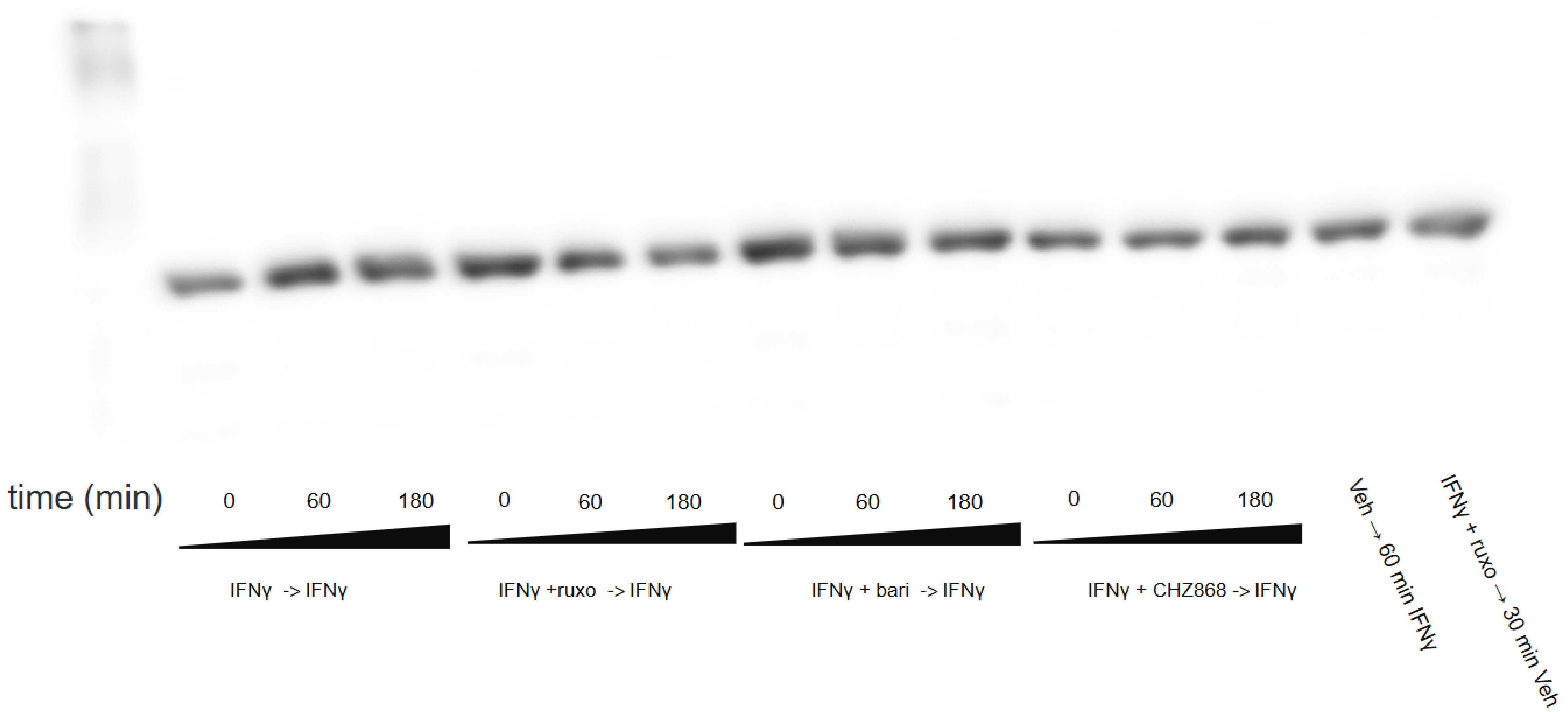

S1 Fig 3 C, pSTAT2

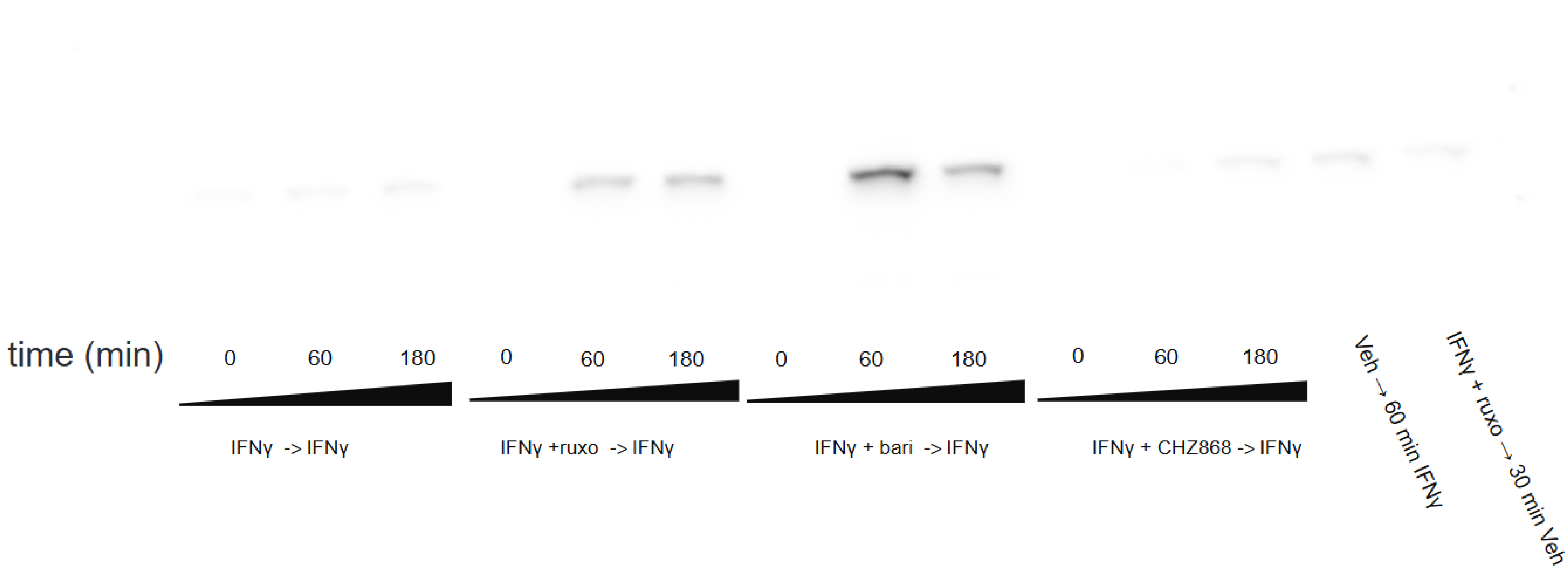

S1 Fig 3 C, GAPDH for pSTAT2

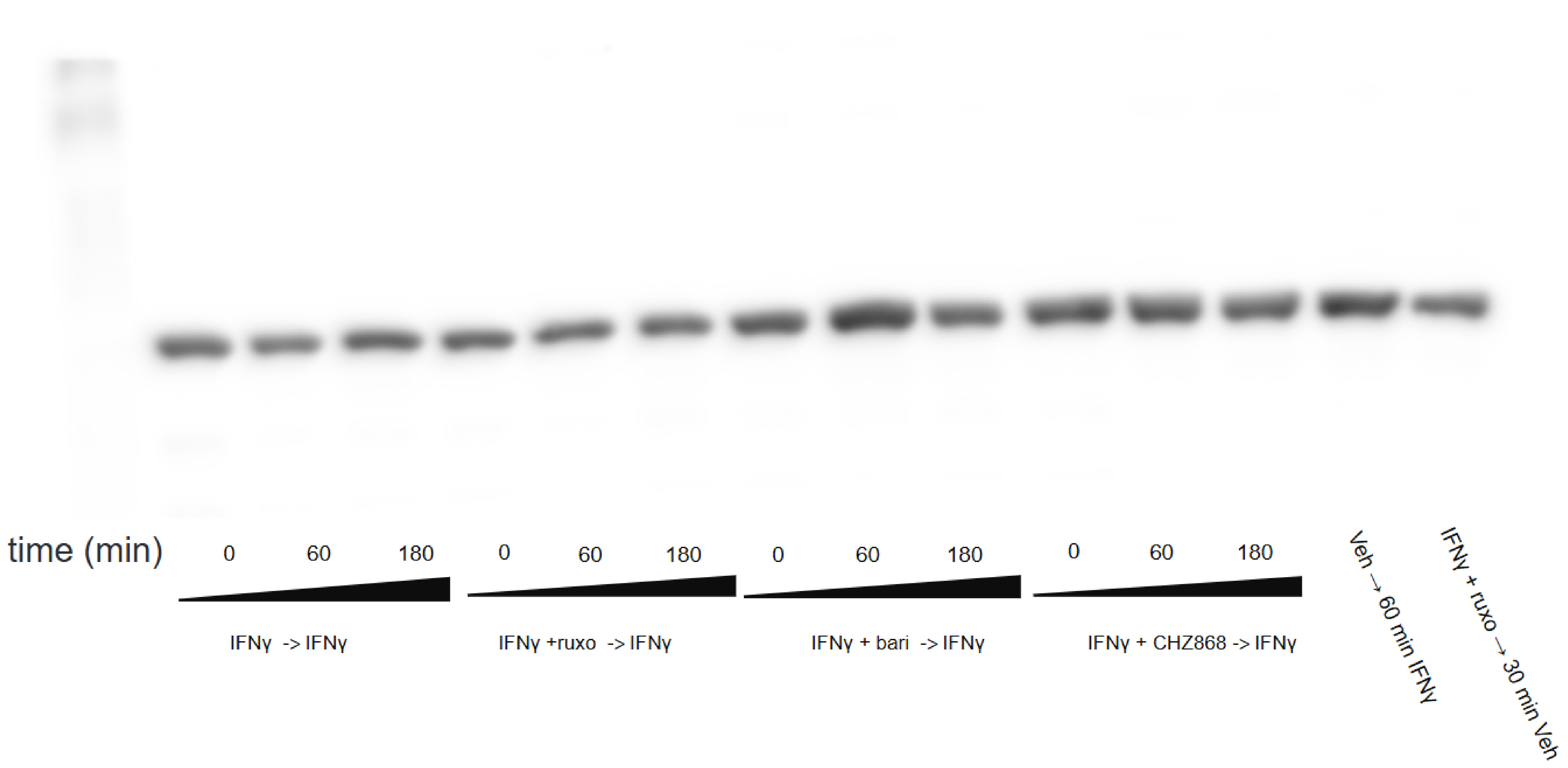

S1 Fig 4 A, tJAK2

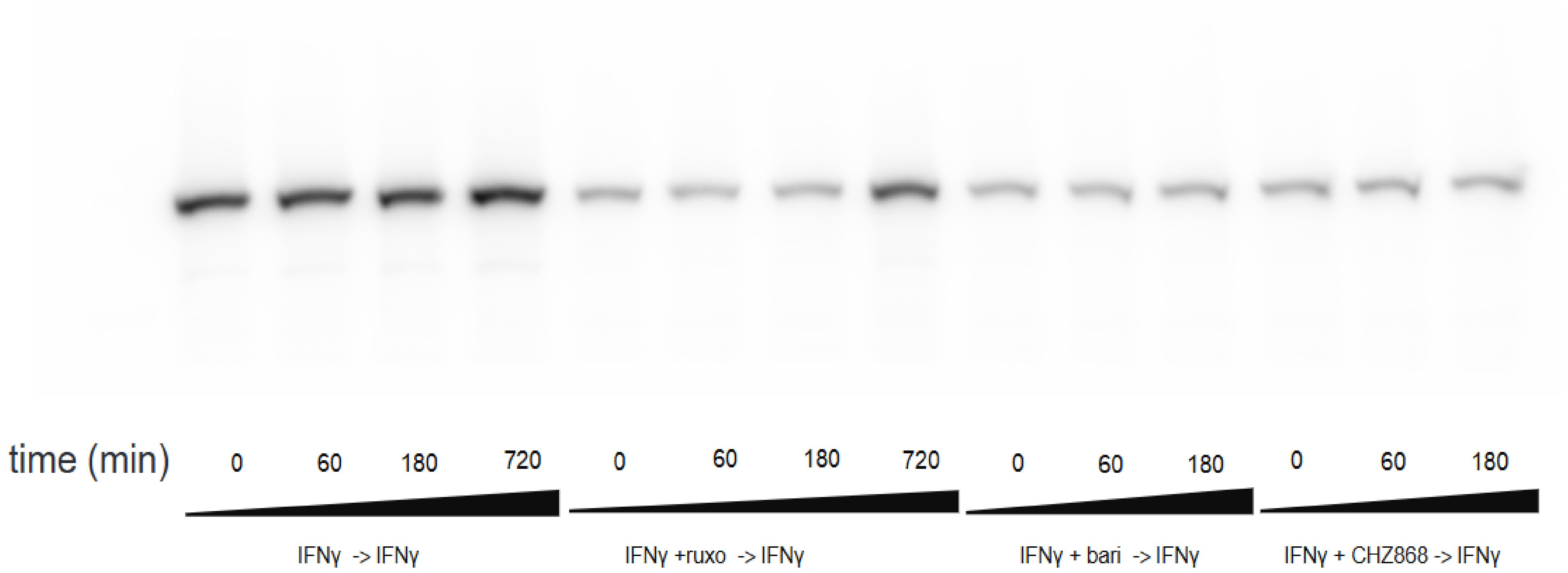

S1 Fig 4 A, GAPDH for tJAK2

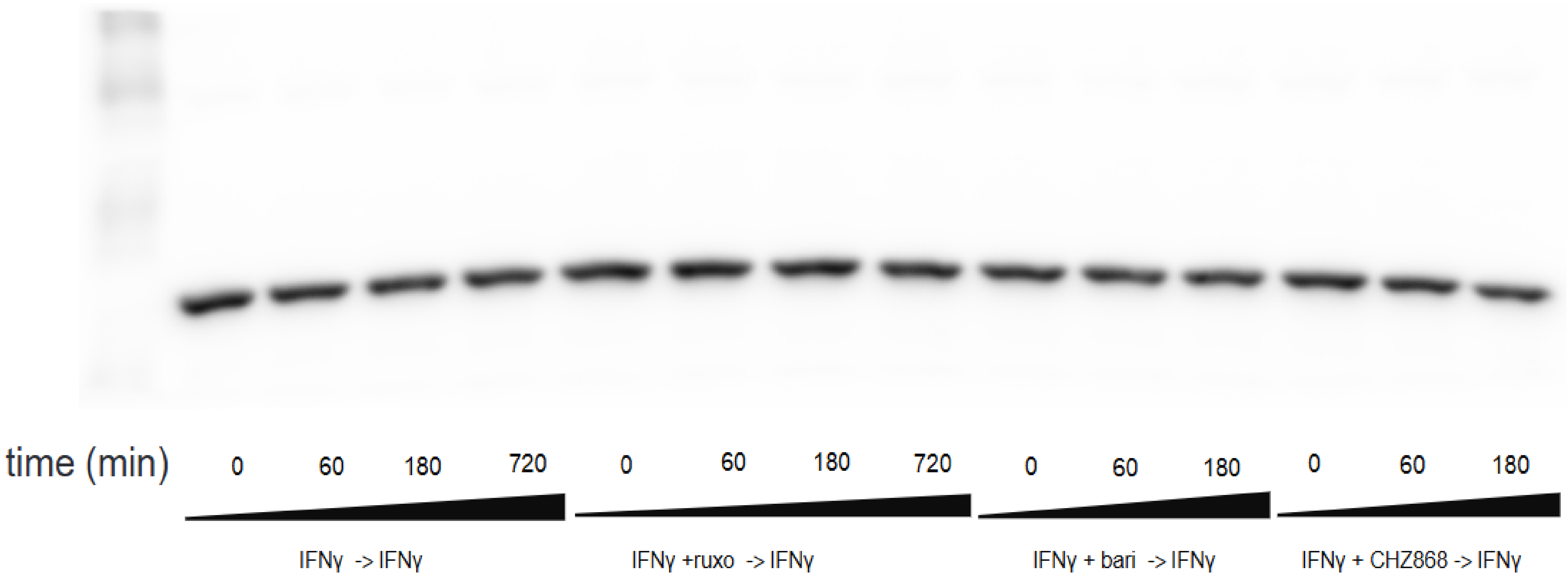

S1 Fig 4 A, pJAK2

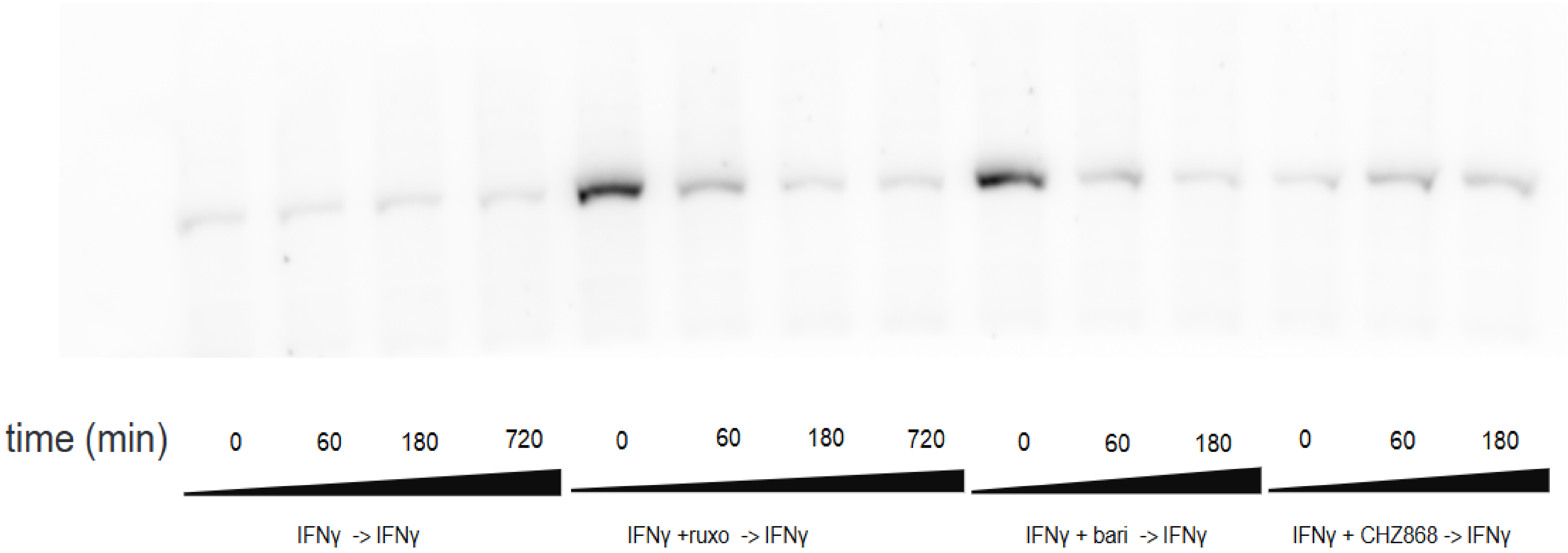

S1 Fig 4 A, GAPDH for pJAK2

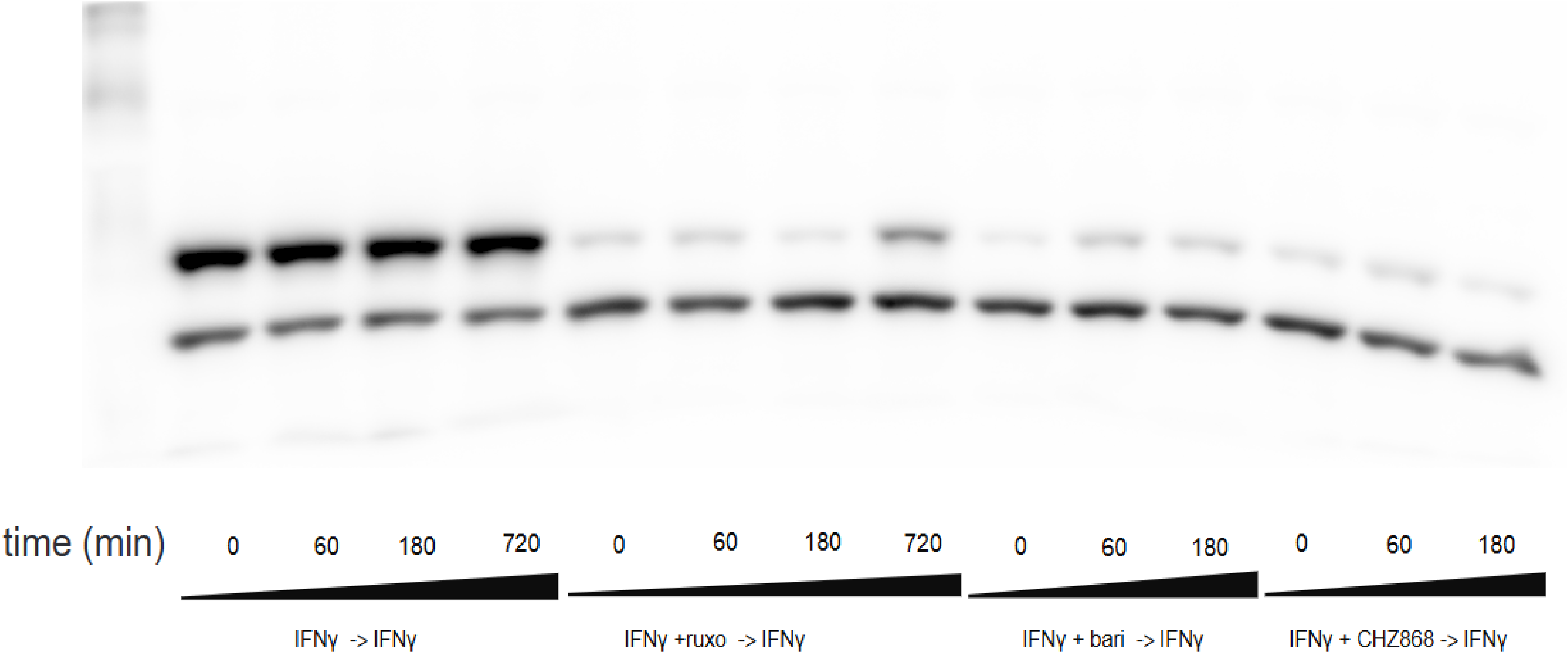

S1 Fig 4 B, tSTAT1

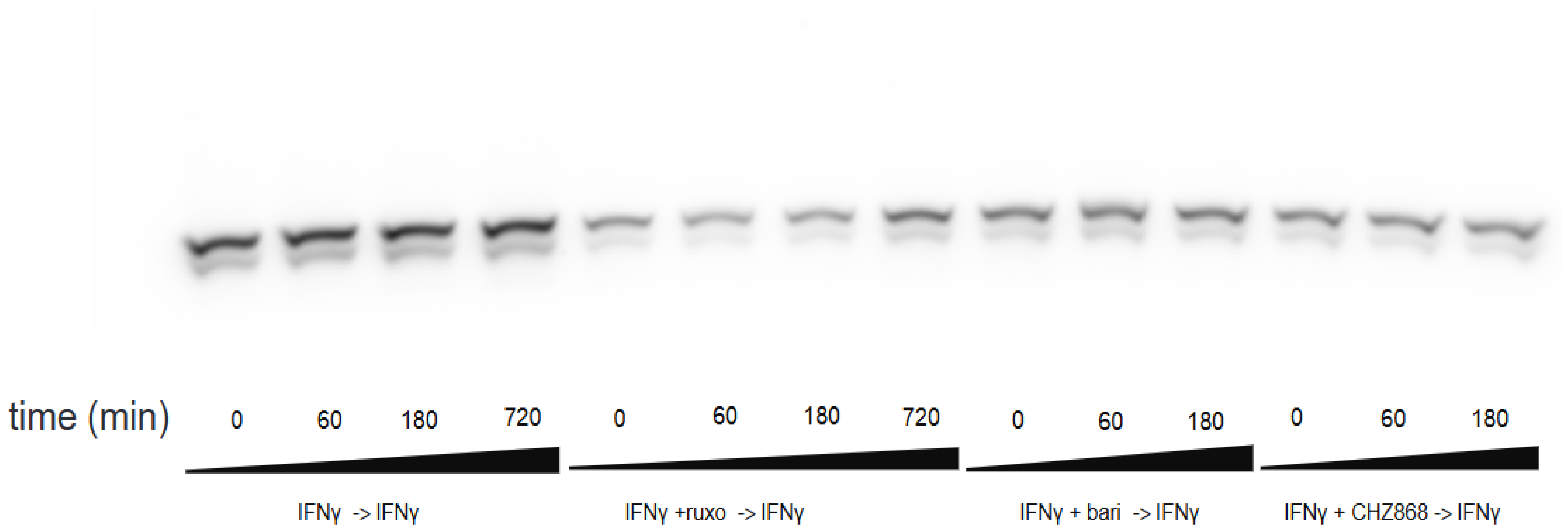

S1 Fig 4 B, GAPDH for tSTAT1

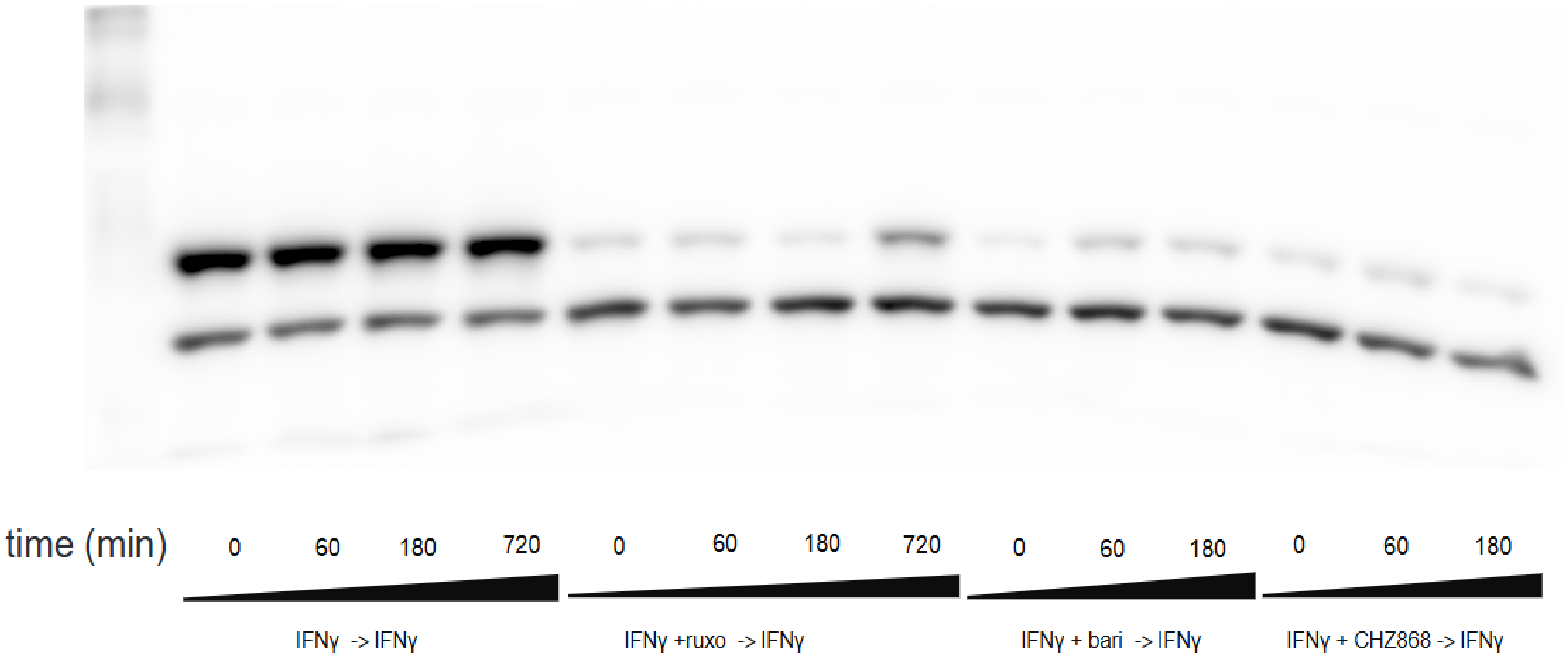

S1 Fig 4 B, pSTAT1

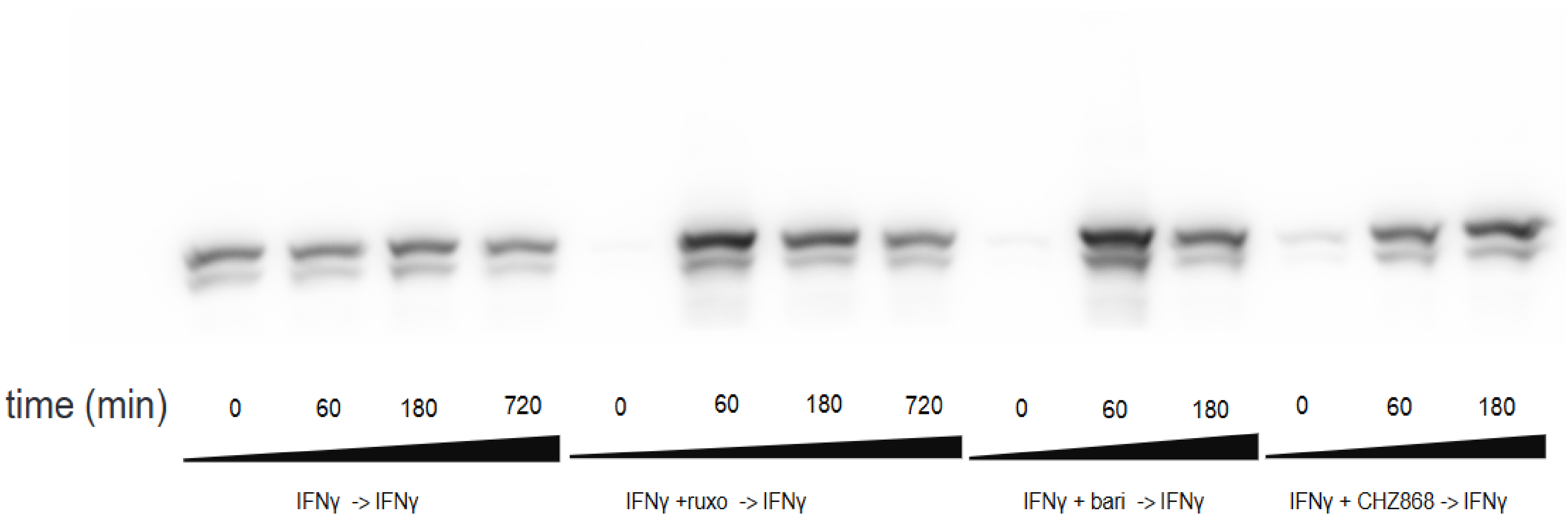

S1 Fig 4 B, GAPDH for pSTAT1

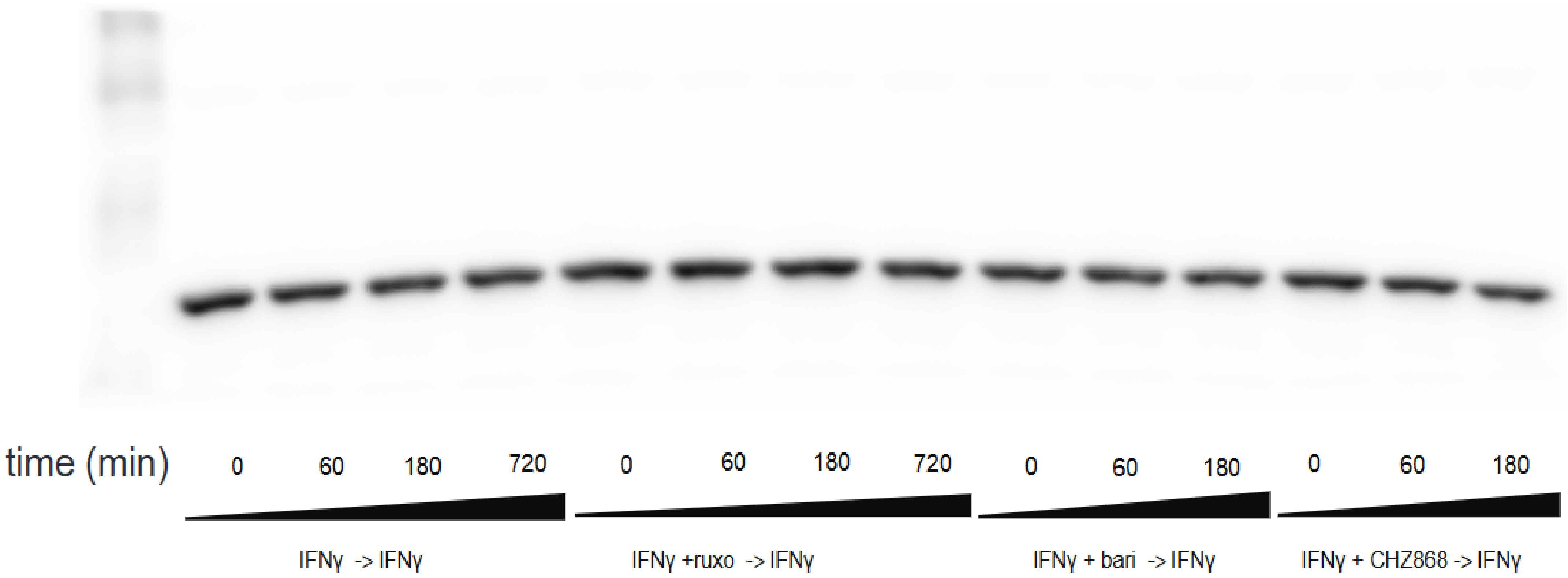

S1 Fig 4 C, tSTAT2

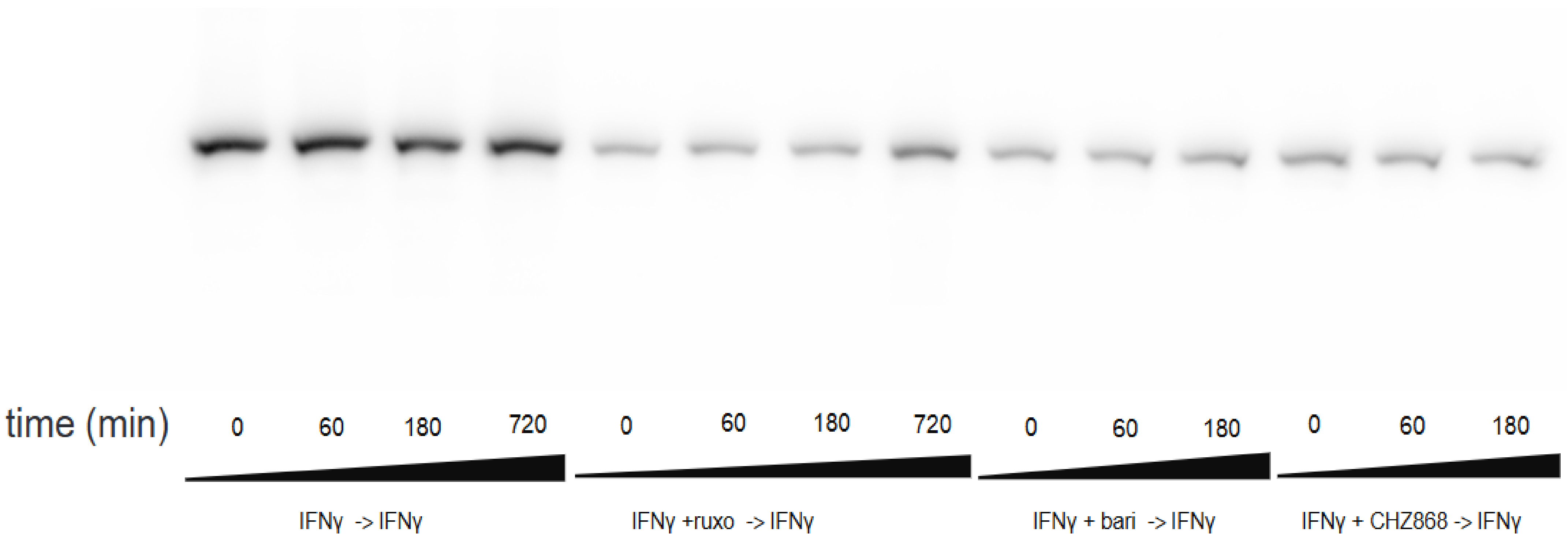

S1 Fig 4 C, GAPDH for tSTAT2

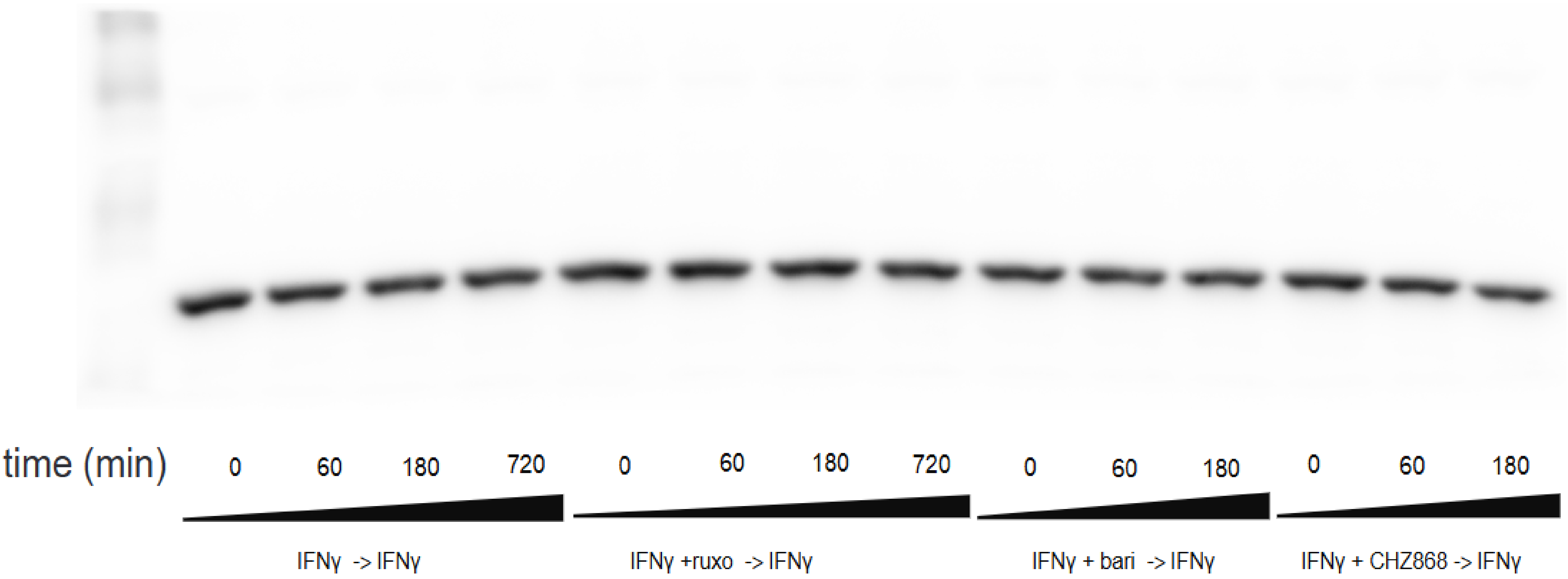

S1 Fig 4 C, pSTAT2

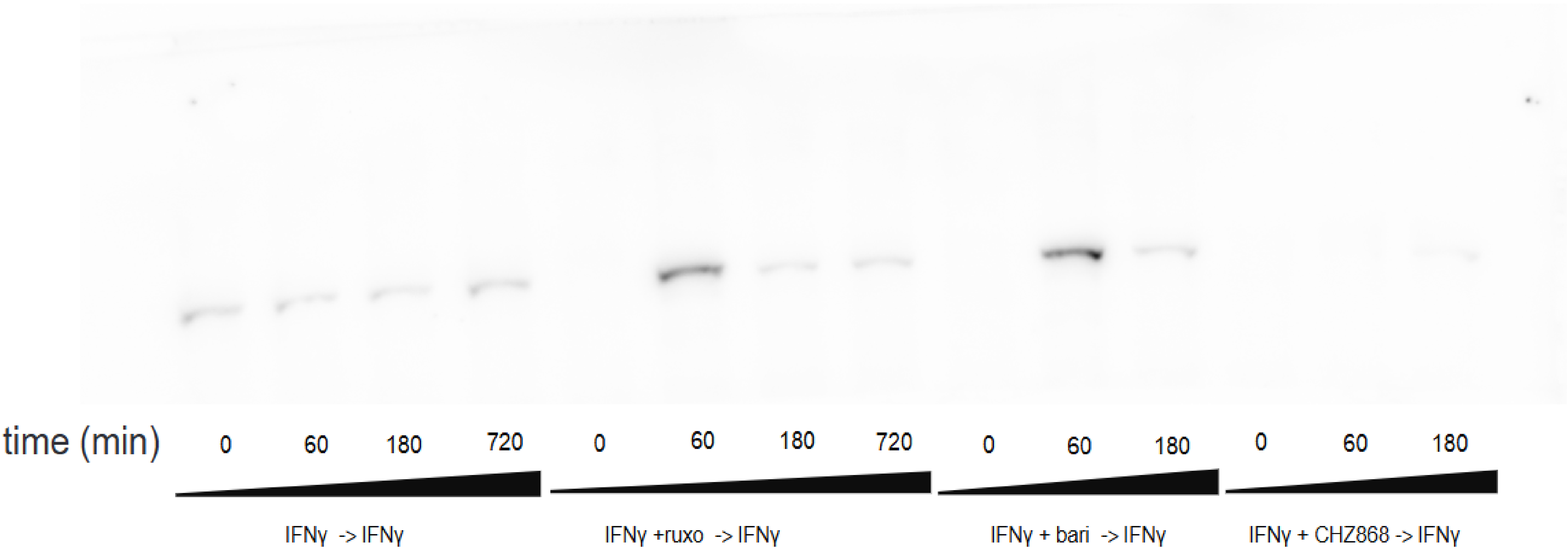

S1 Fig 4 C, GAPDH for pSTAT2

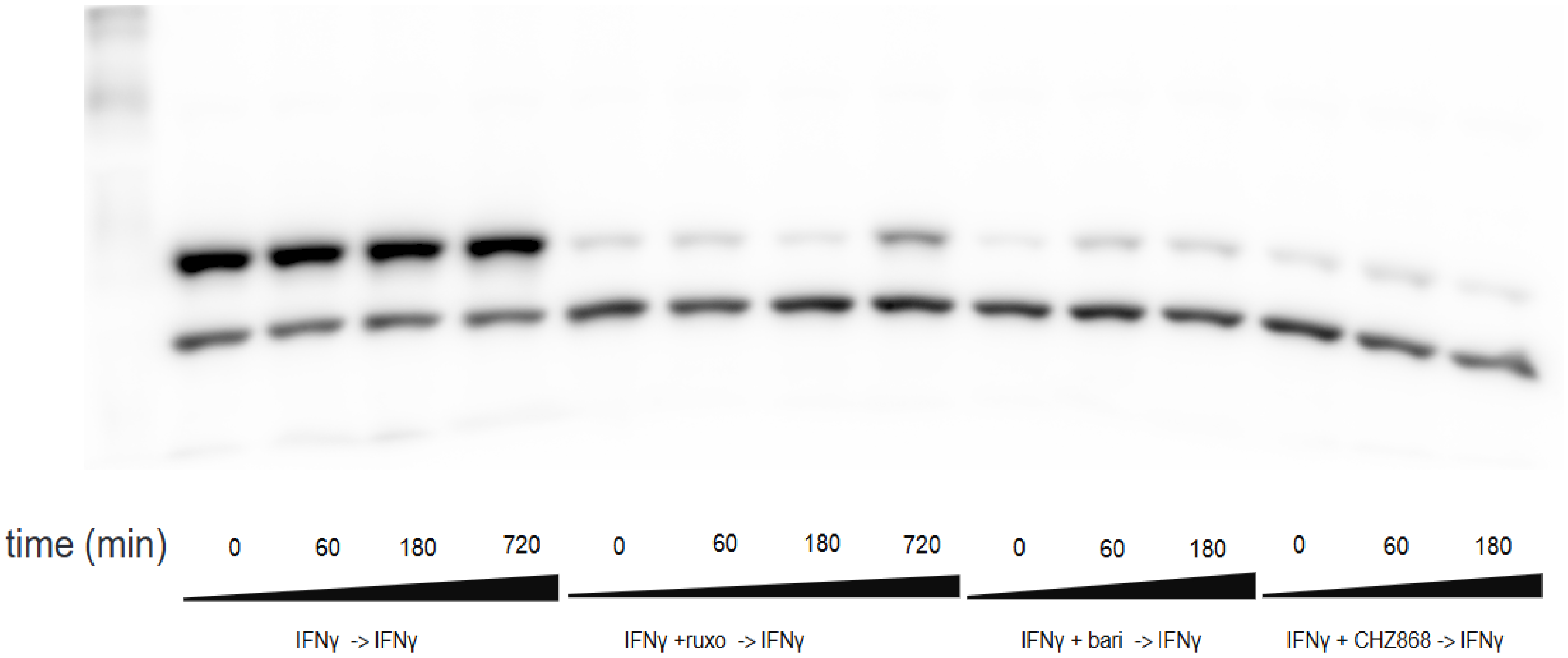

S1 Fig S3 Fig, pSTAT2

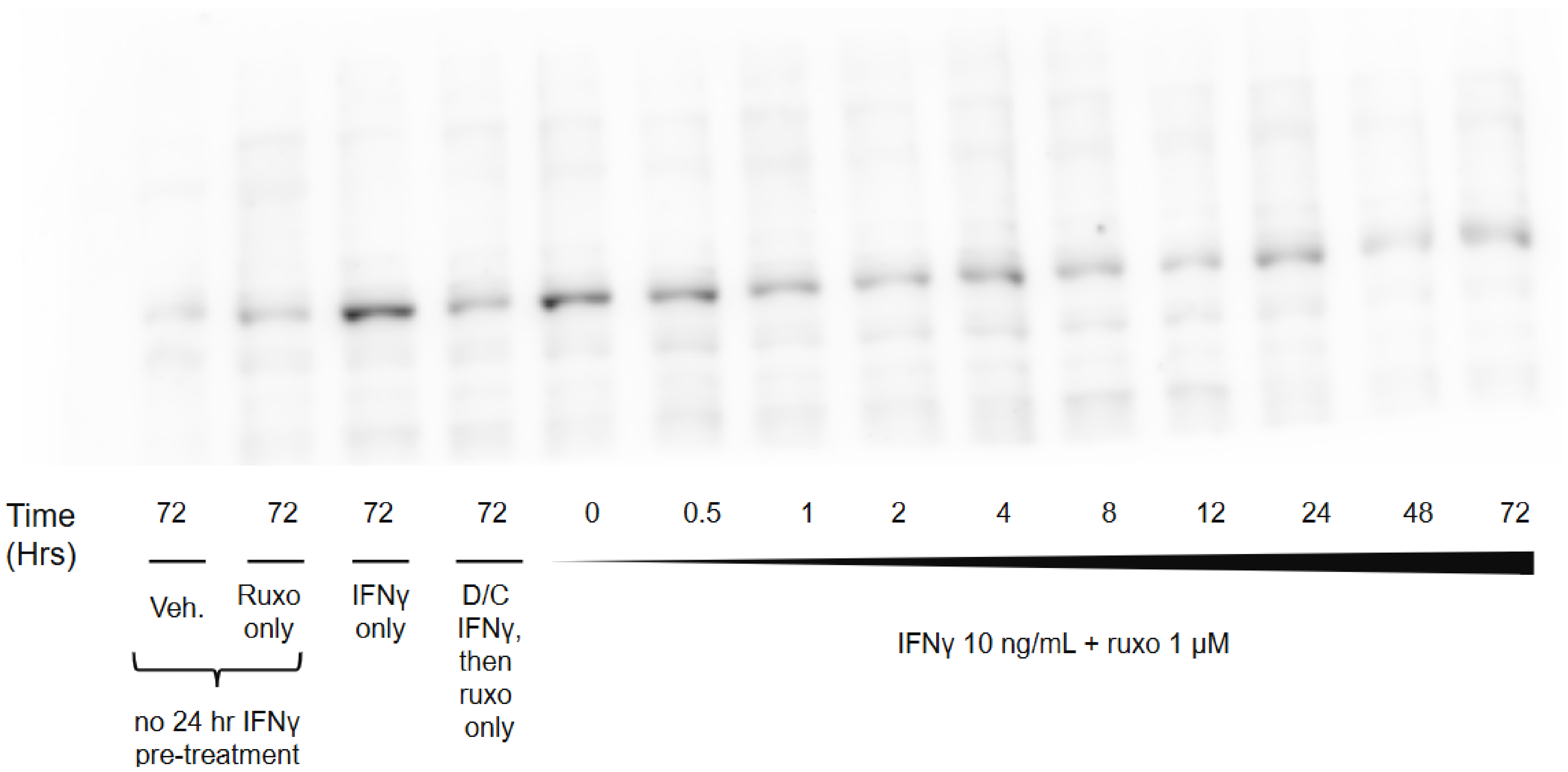

S1 Fig S3 Fig, β-actin

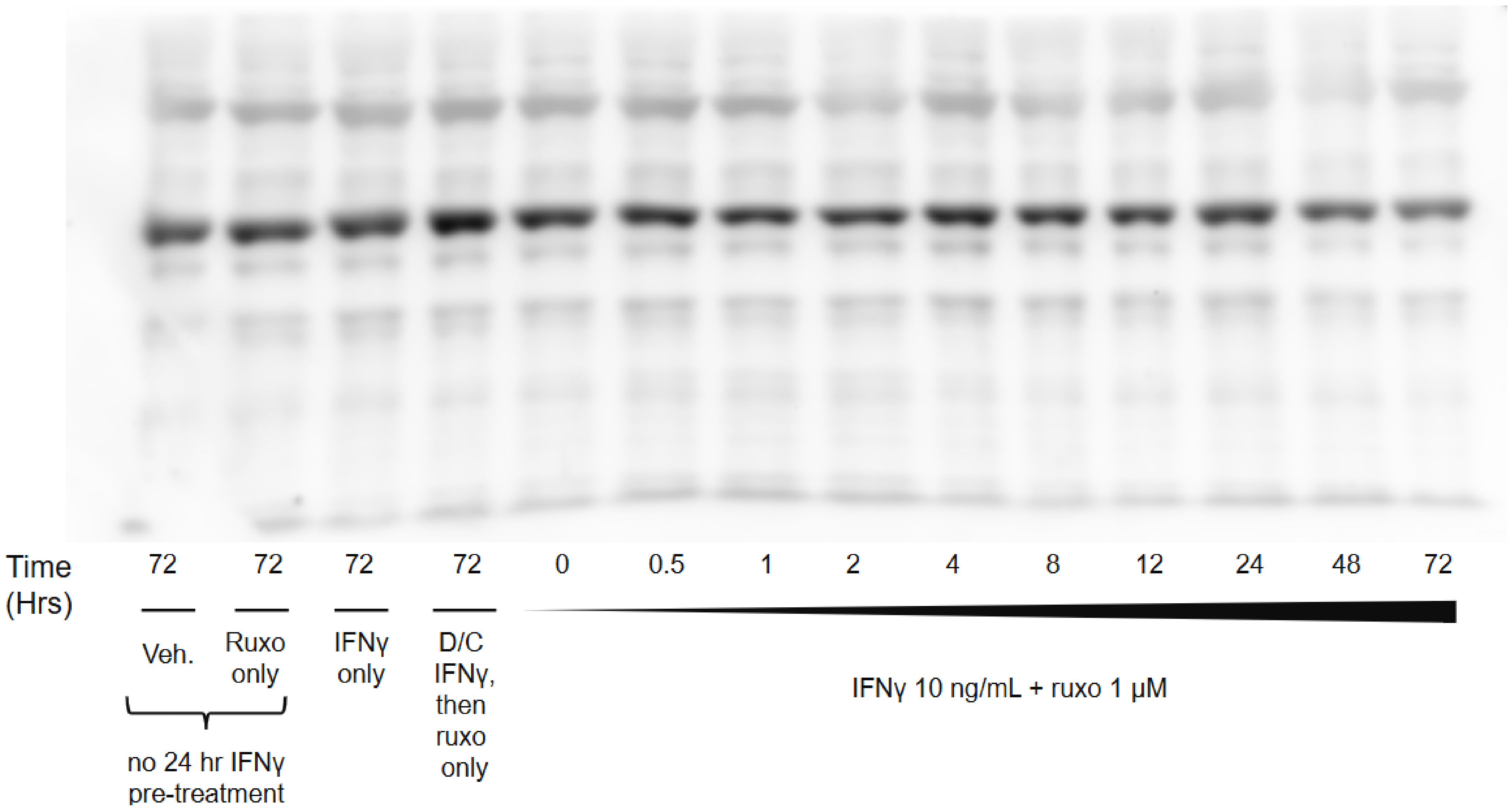

S3 Fig, tSTAT3

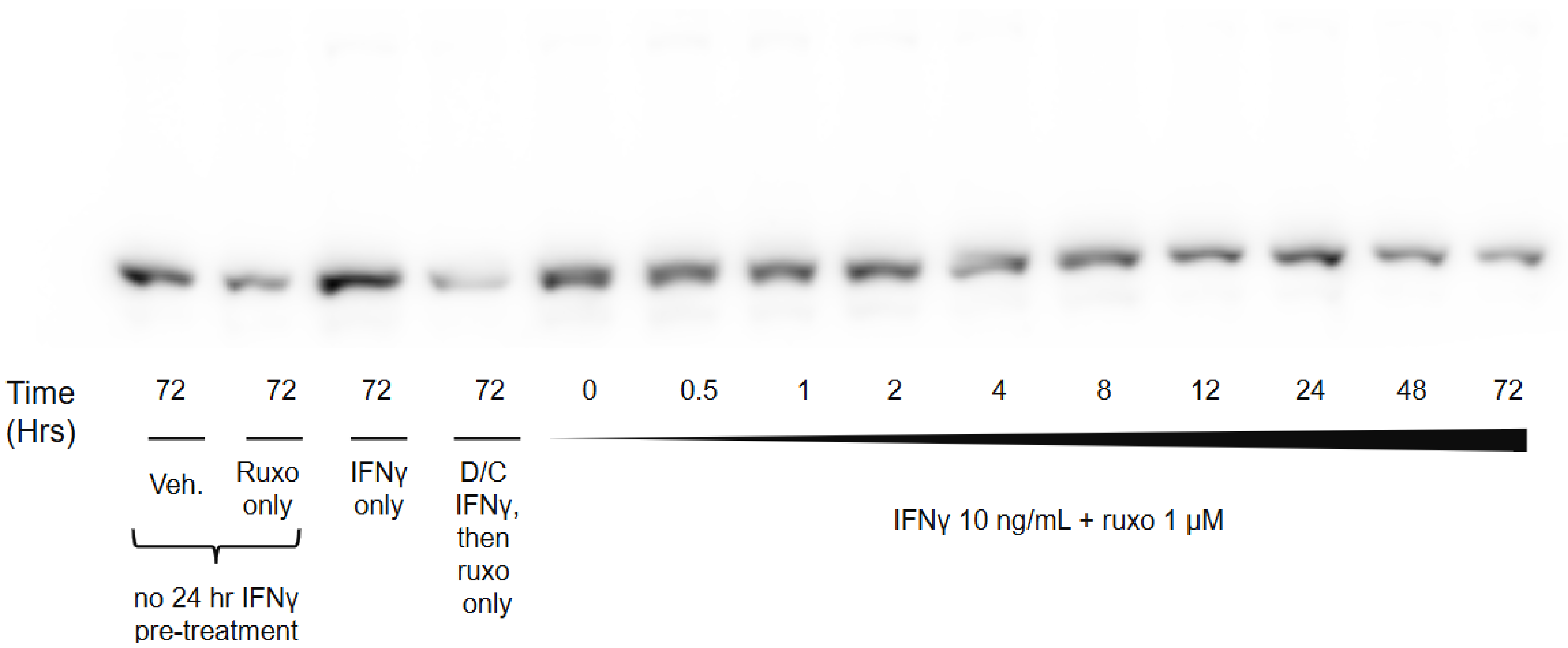

S1 Fig S3 Fig, pSTAT3

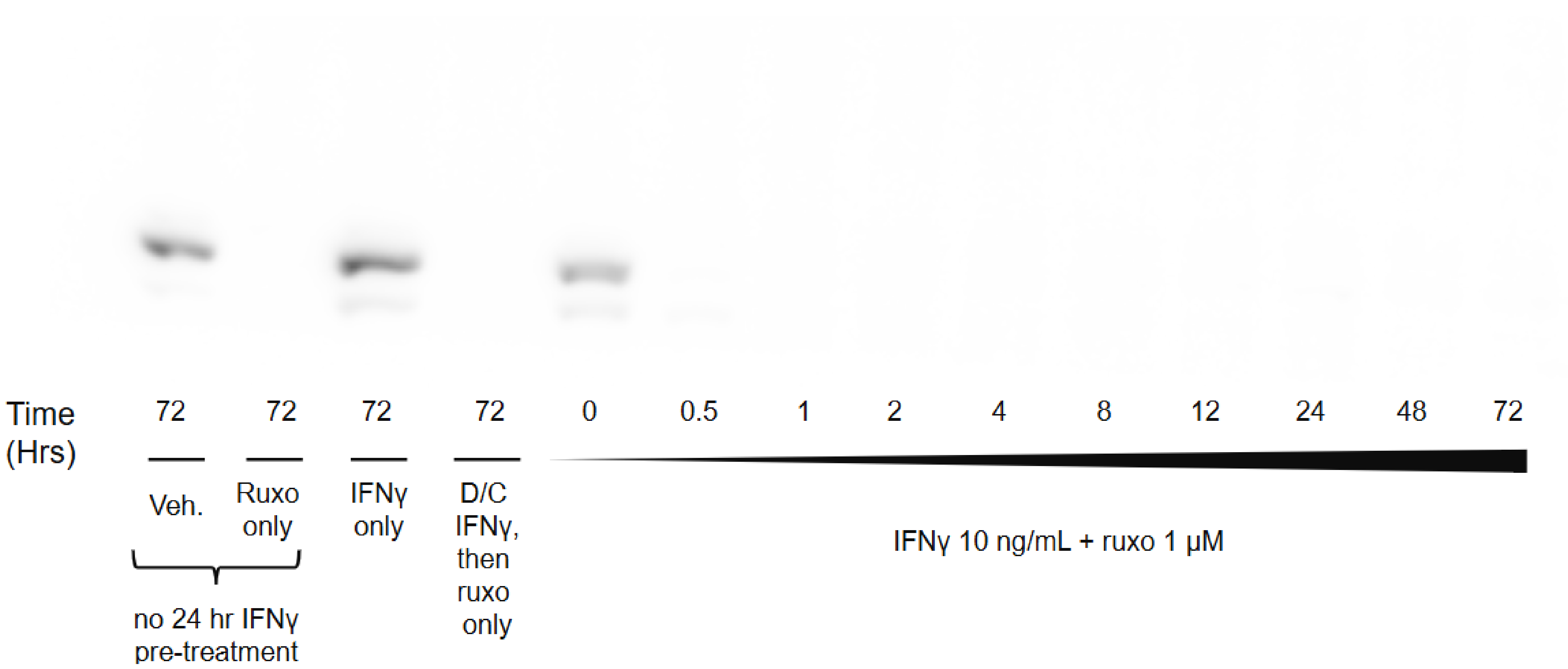

S1 Fig S3 Fig, GAPDH

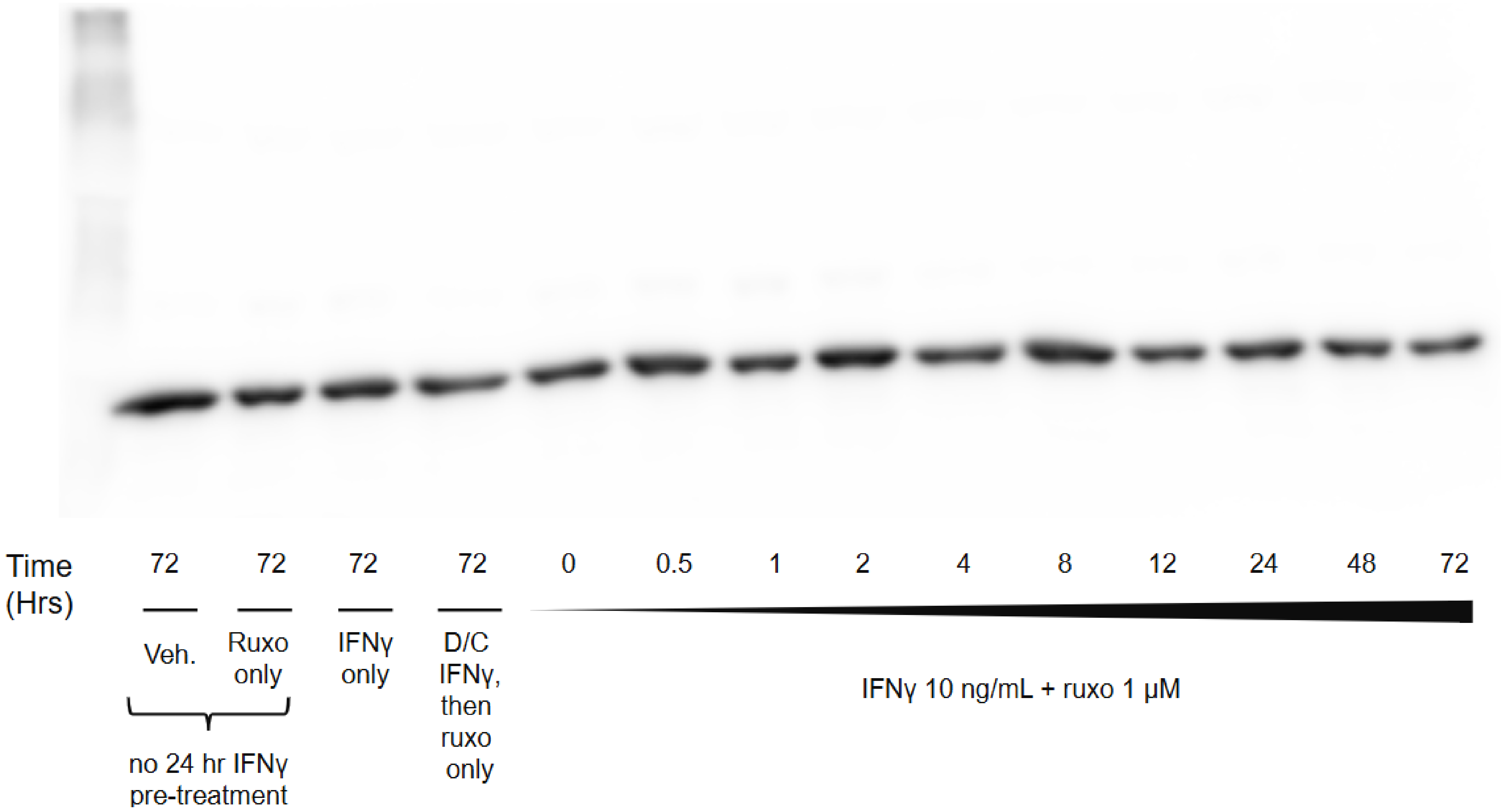

S1 Fig S3 Fig, tSTAT5

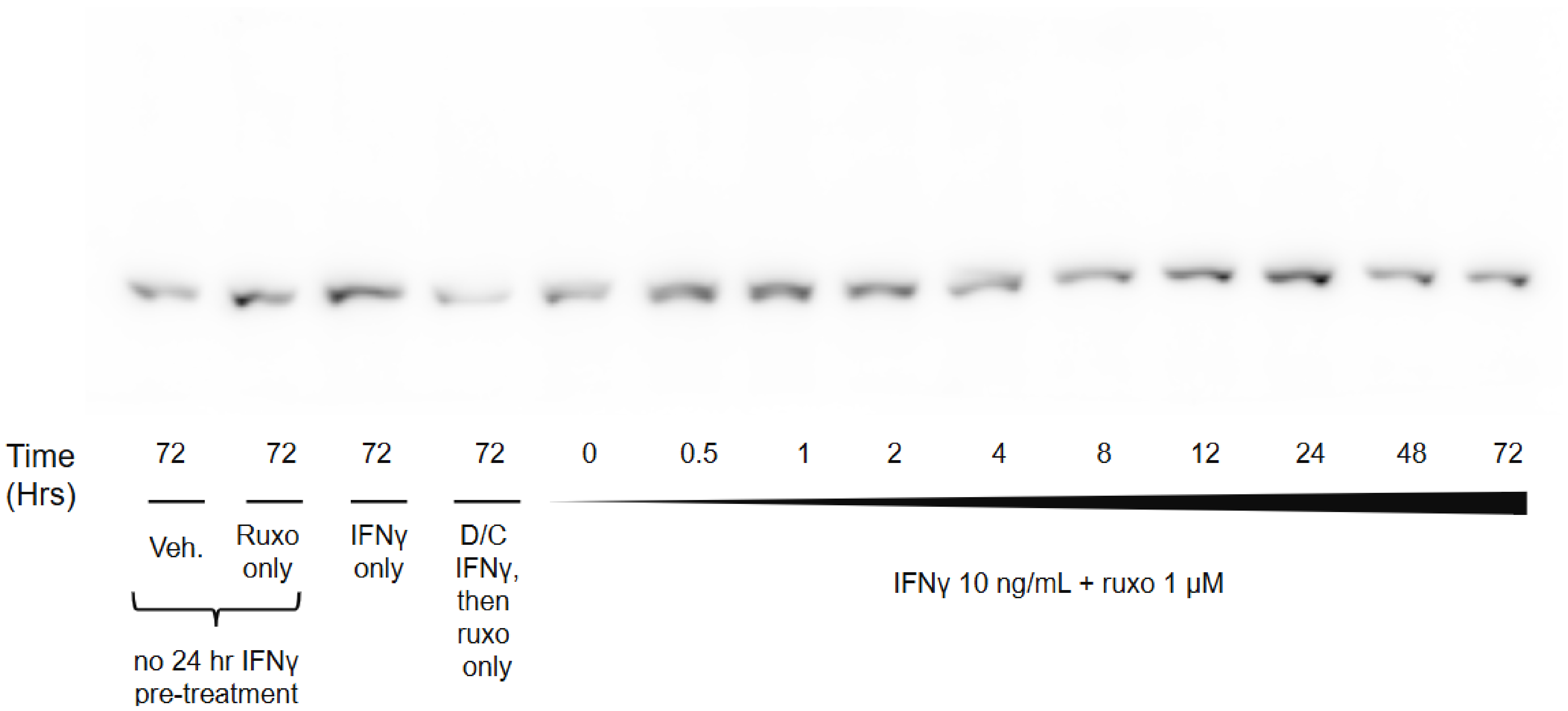

S1 Fig S3 Fig, pSTAT5

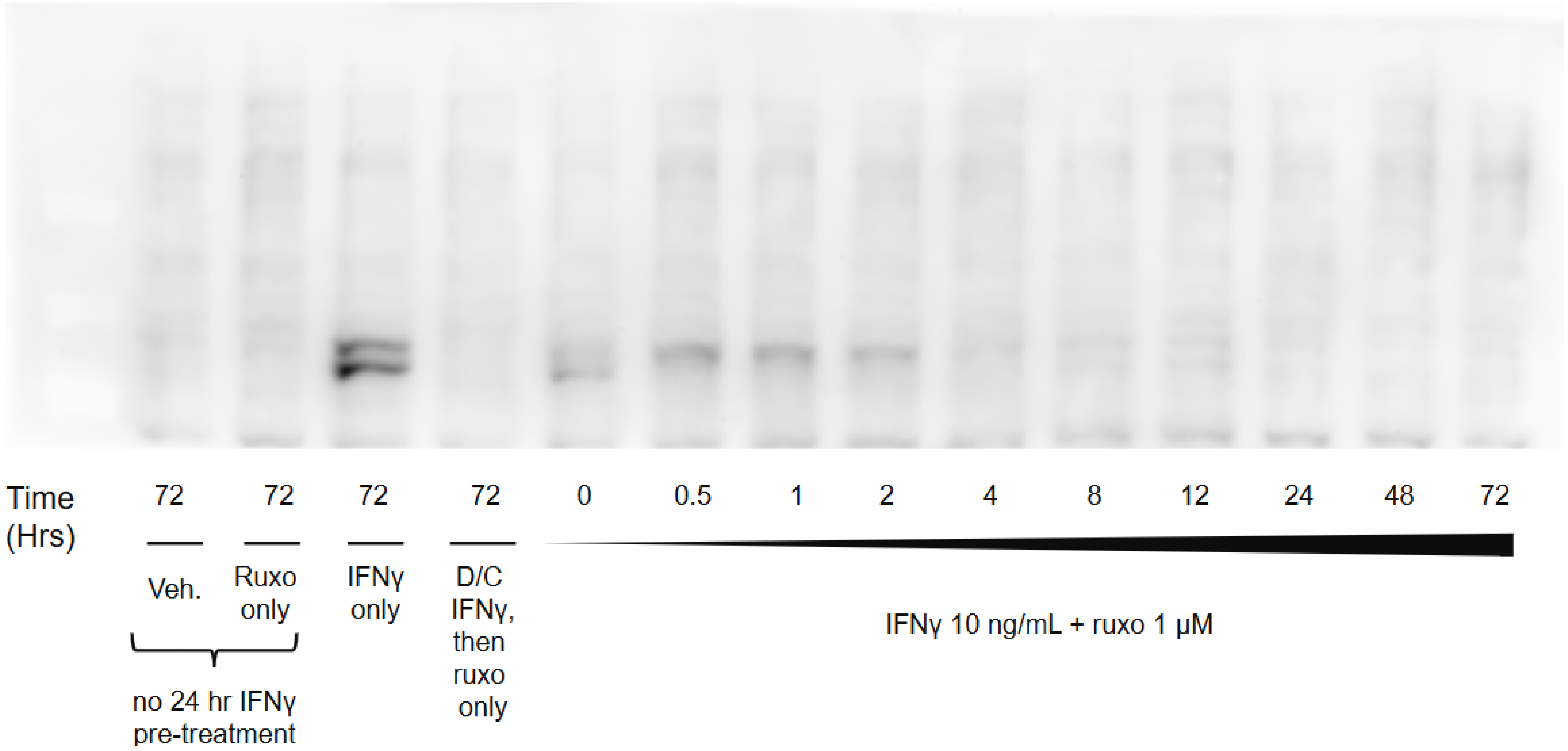

S1 Fig S3 Fig, GAPDH

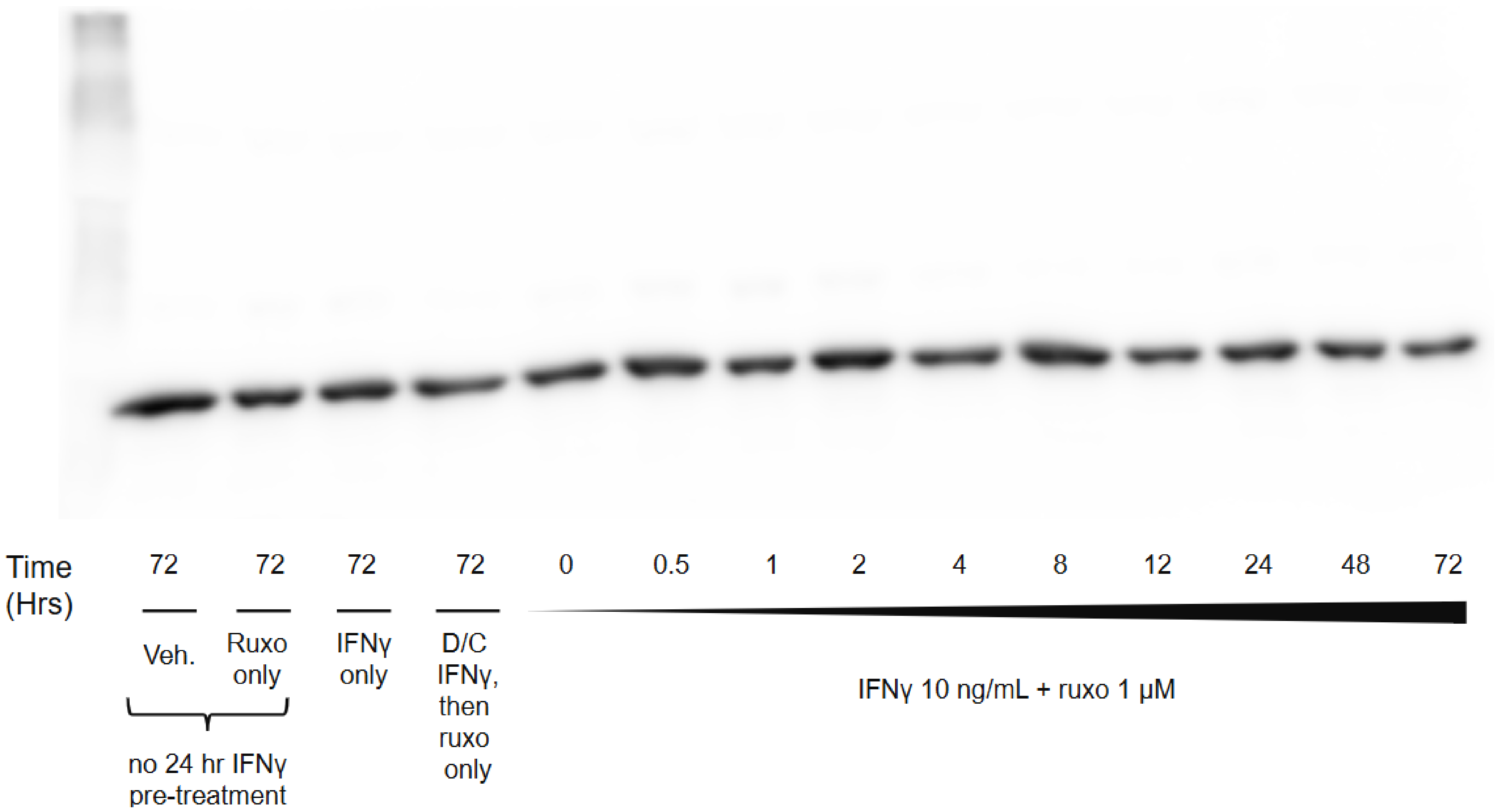

S1 Fig S3 Fig, tTYK2

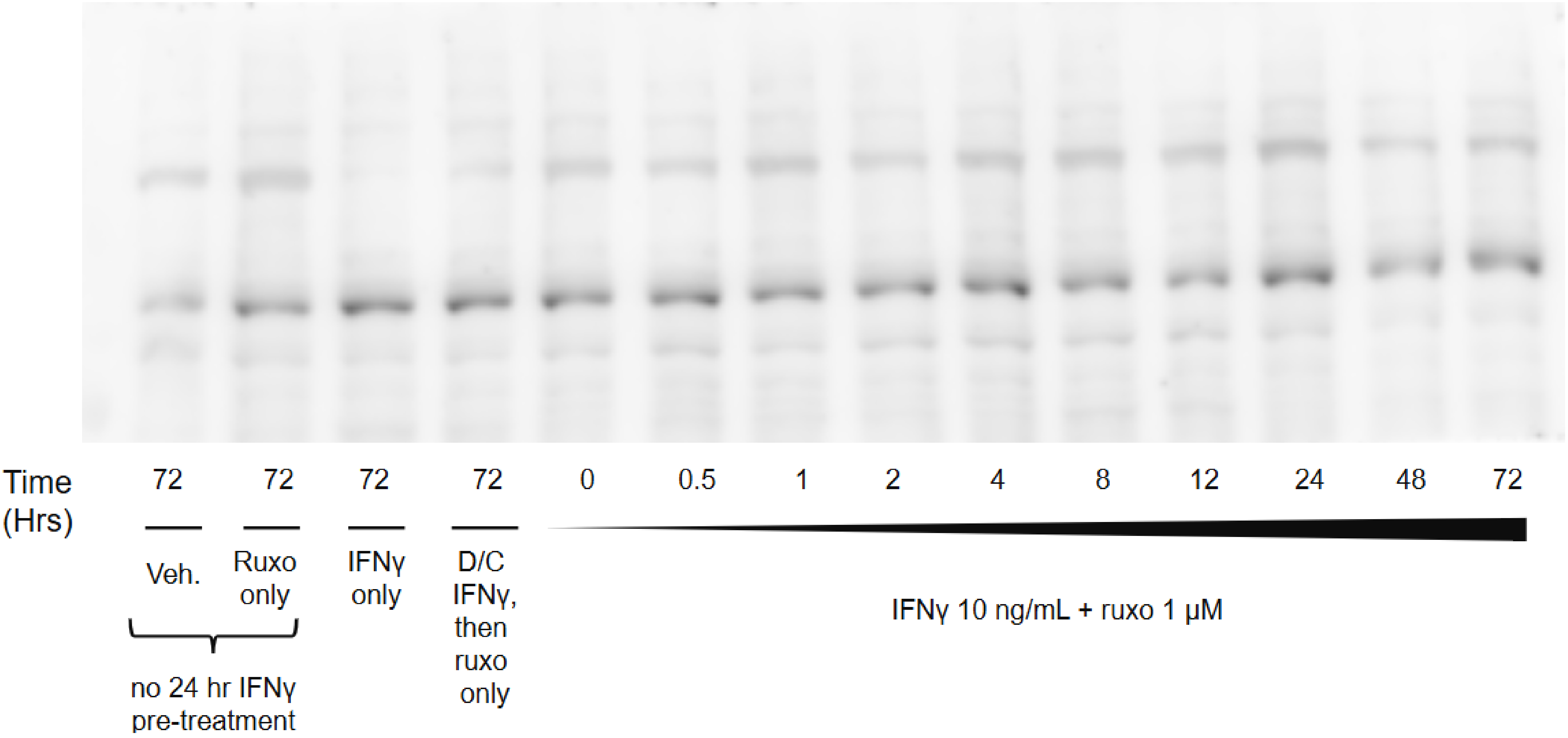

S1 Fig S3 Fig, pTYK2

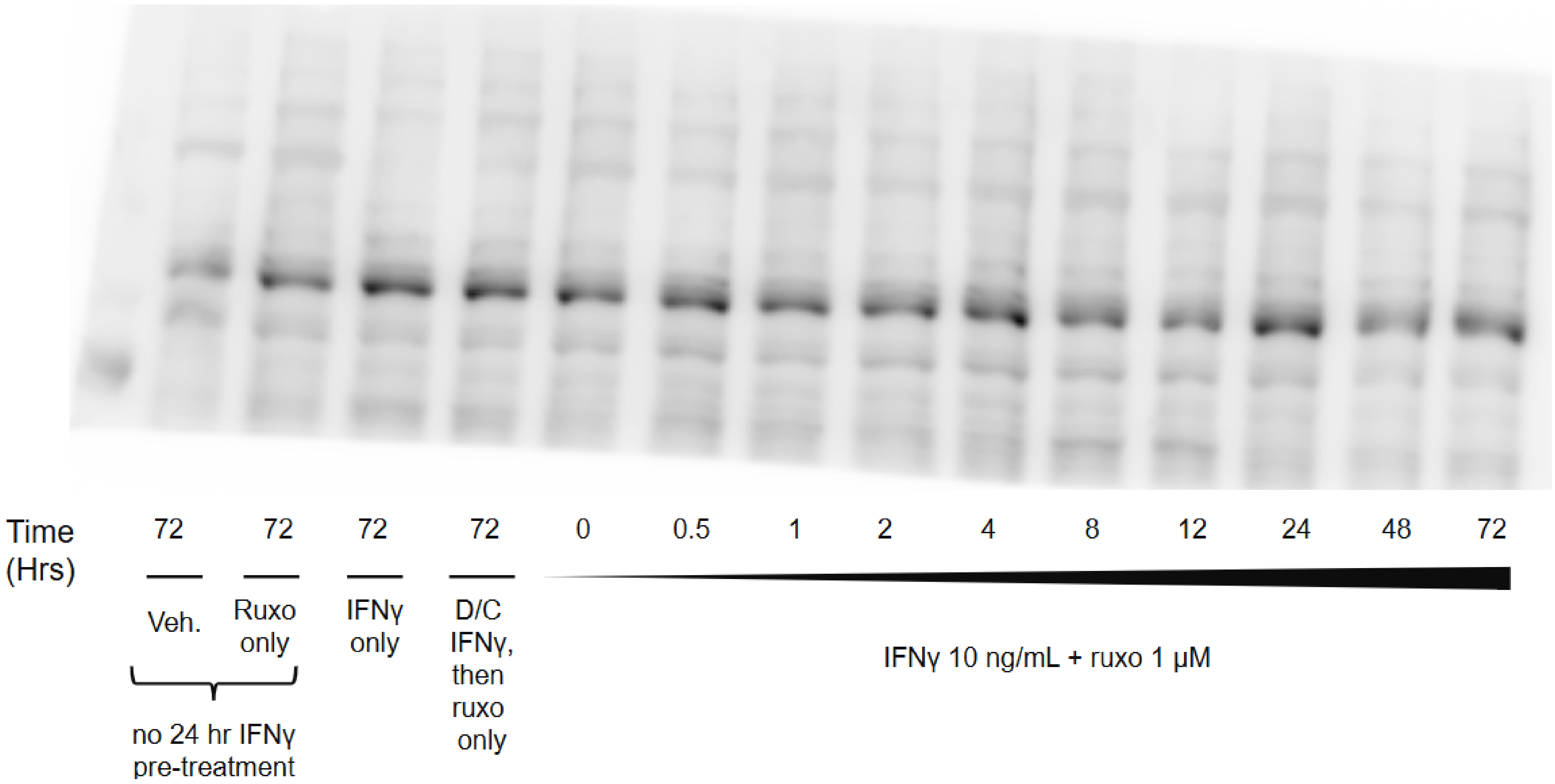

S1 Fig S3 Fig, β-actin

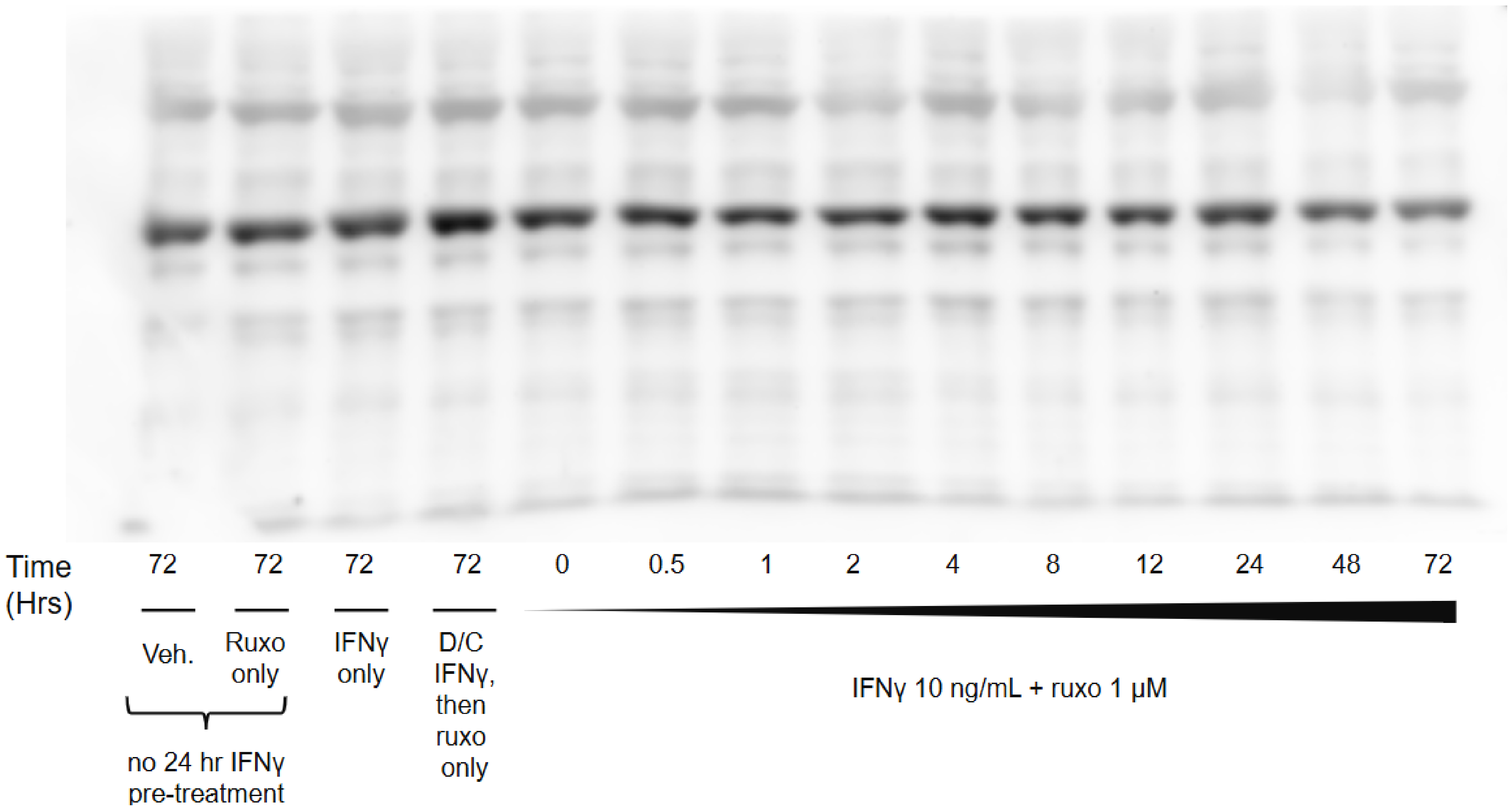

S1 Fig S3 Fig, c-Jun

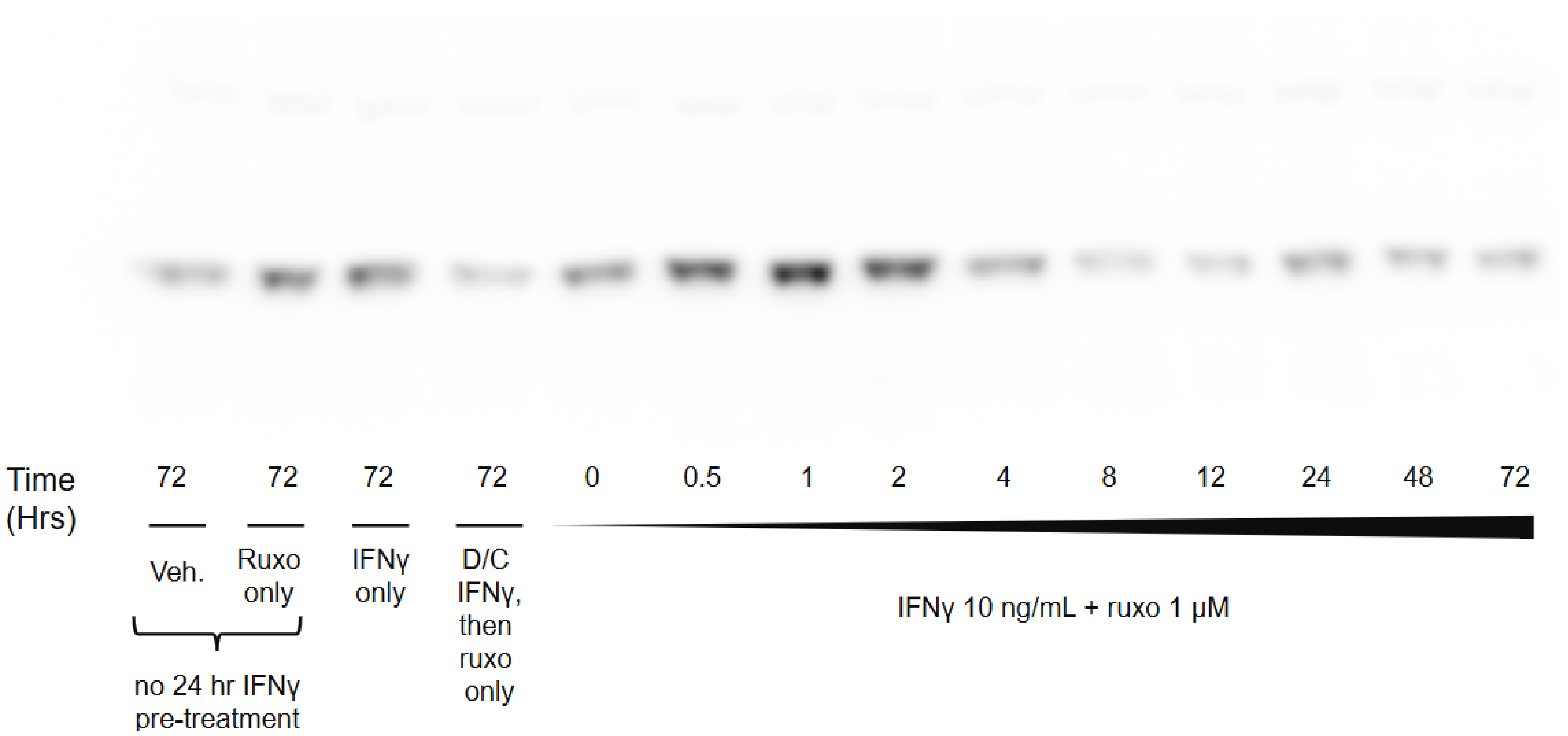

S1 Fig S3 Fig, cyclin D1

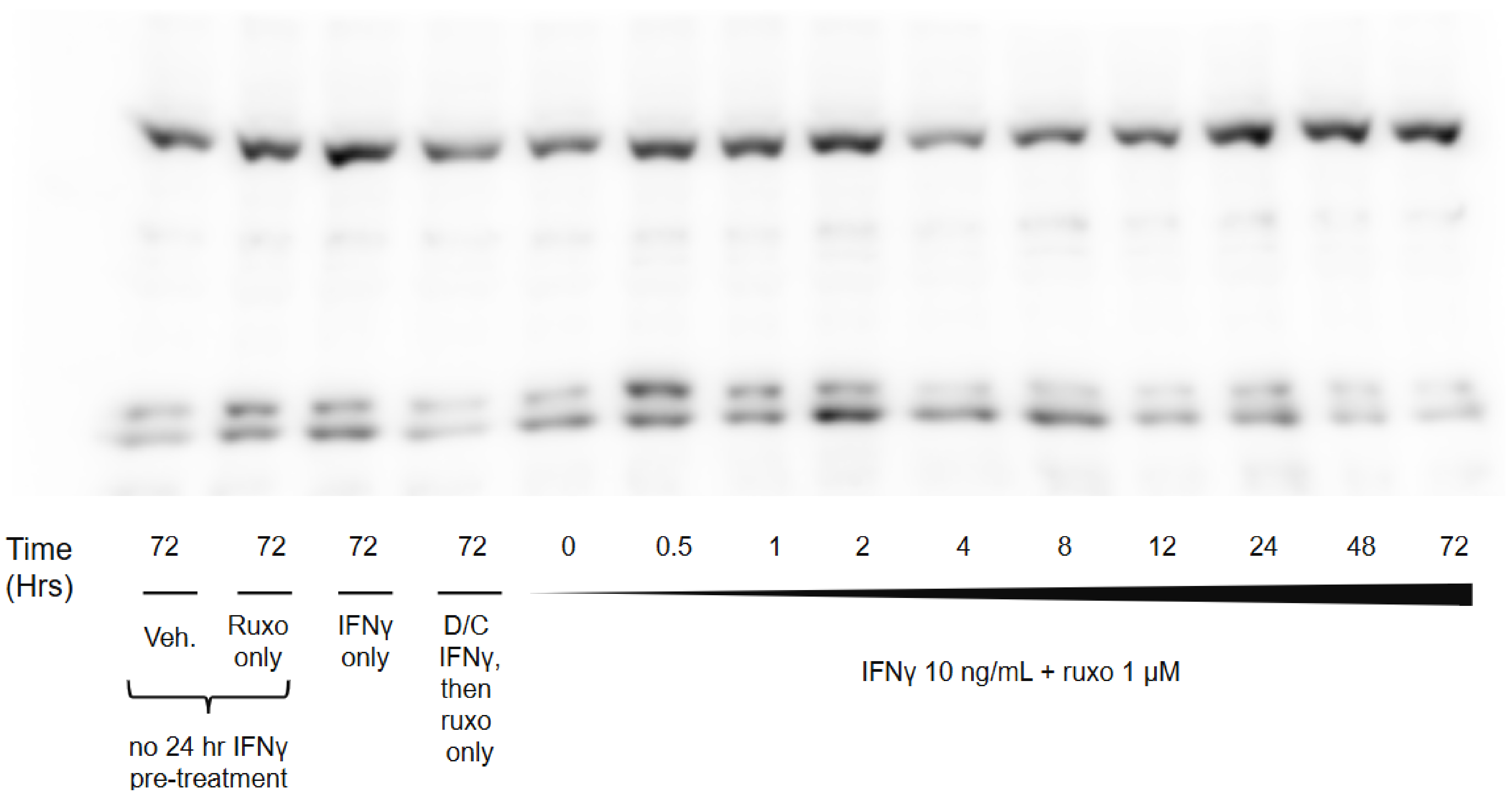

S1 Fig S3 Fig, GAPDH

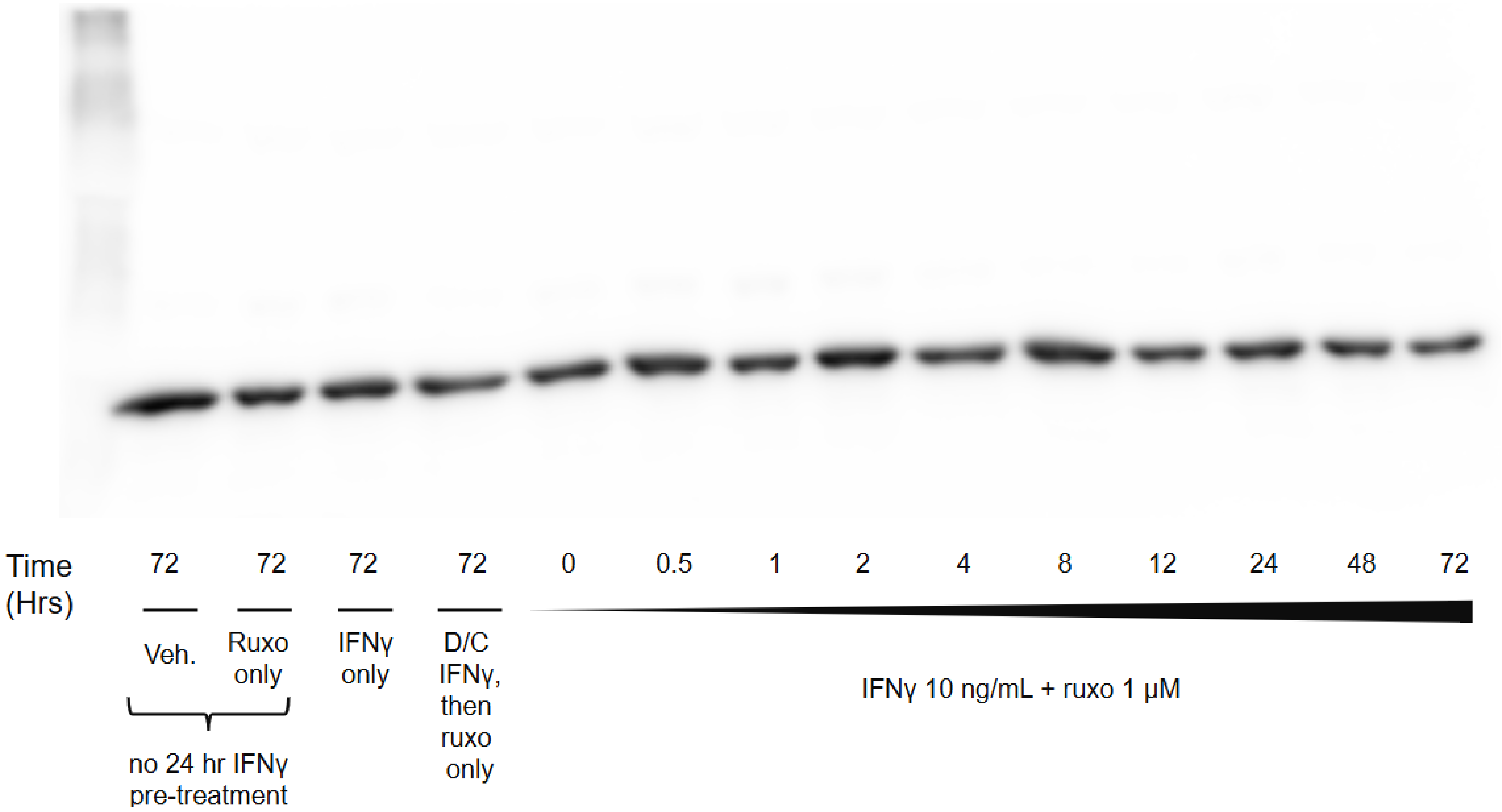

S1 Fig S4 Fig, pJAK1

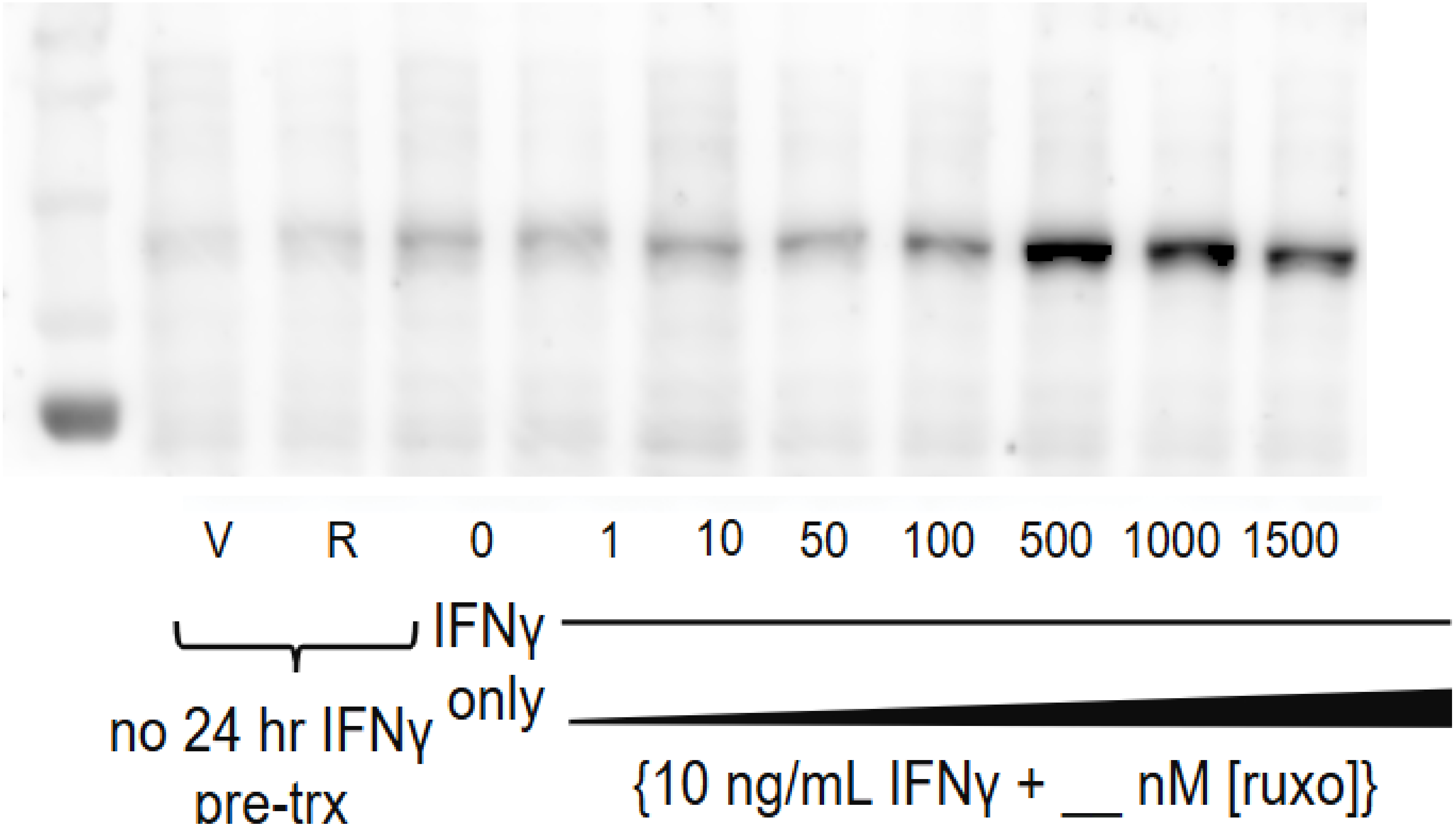

S1 Fig S4 Fig, GAPDH for pJAK1

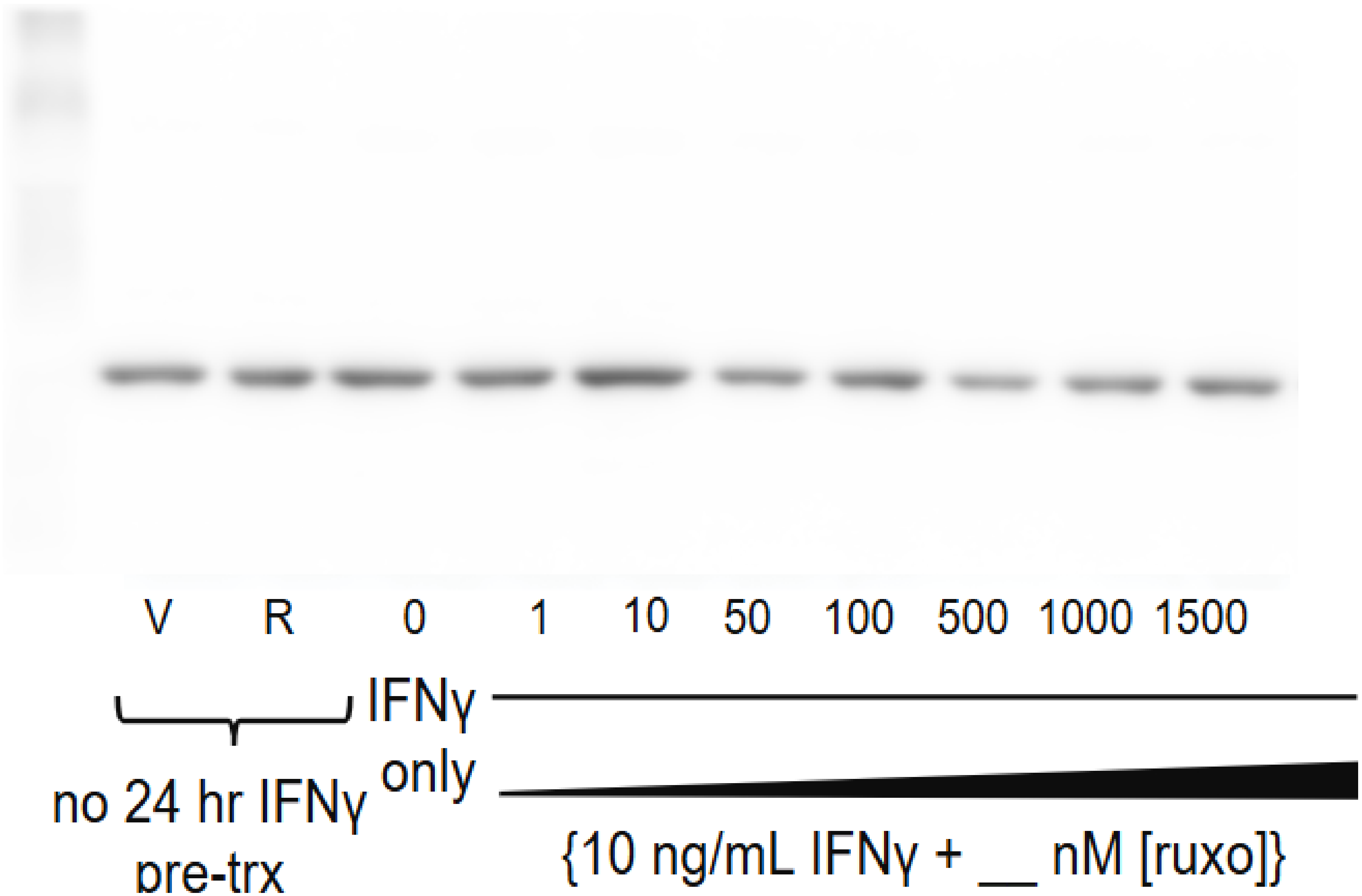

S1 Fig S4 Fig, pJAK2

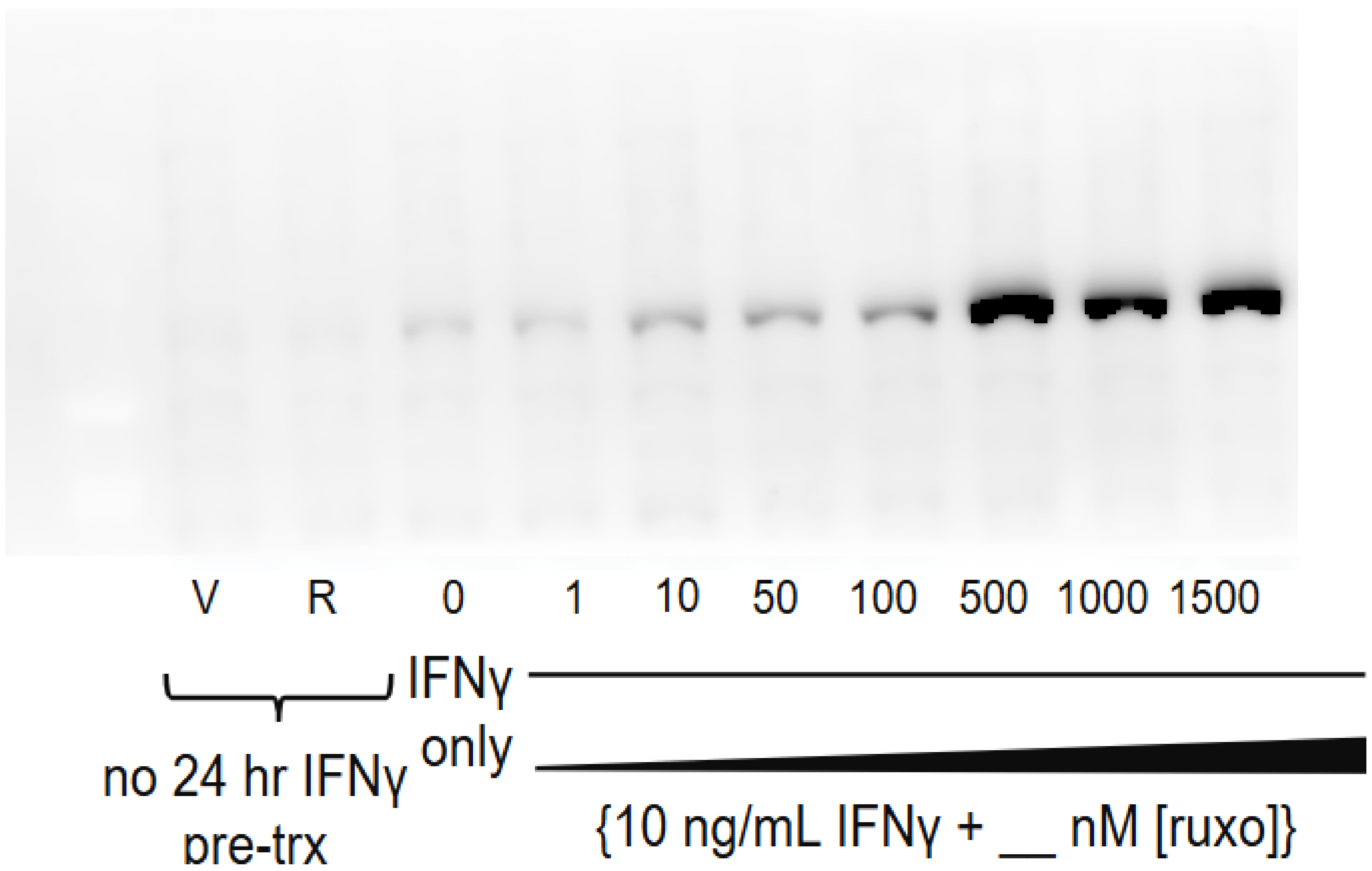

S1 Fig S4 Fig. GAPDH for pJAK2

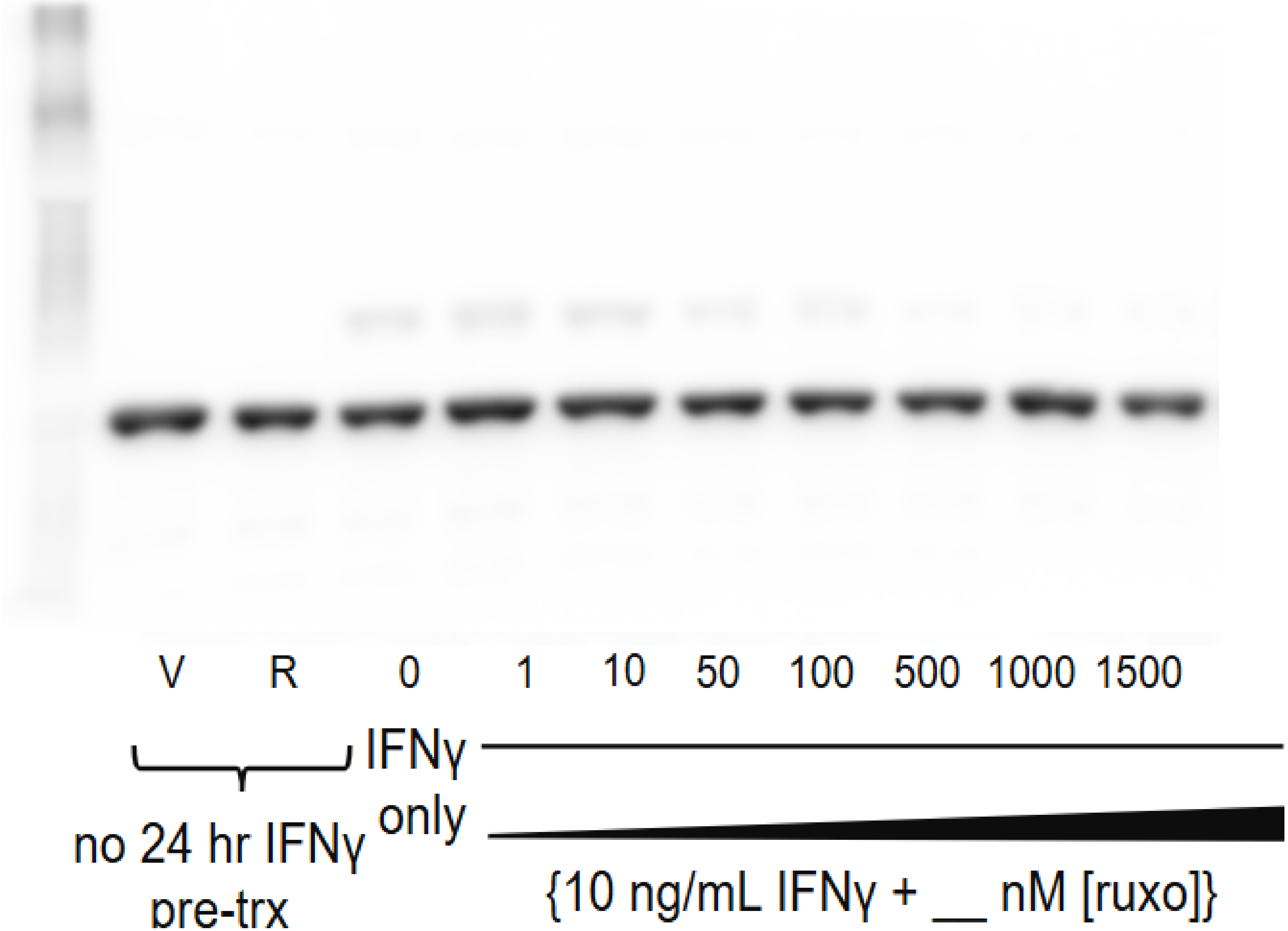

S1 Fig S4 Fig, GAPDH for pJAK2

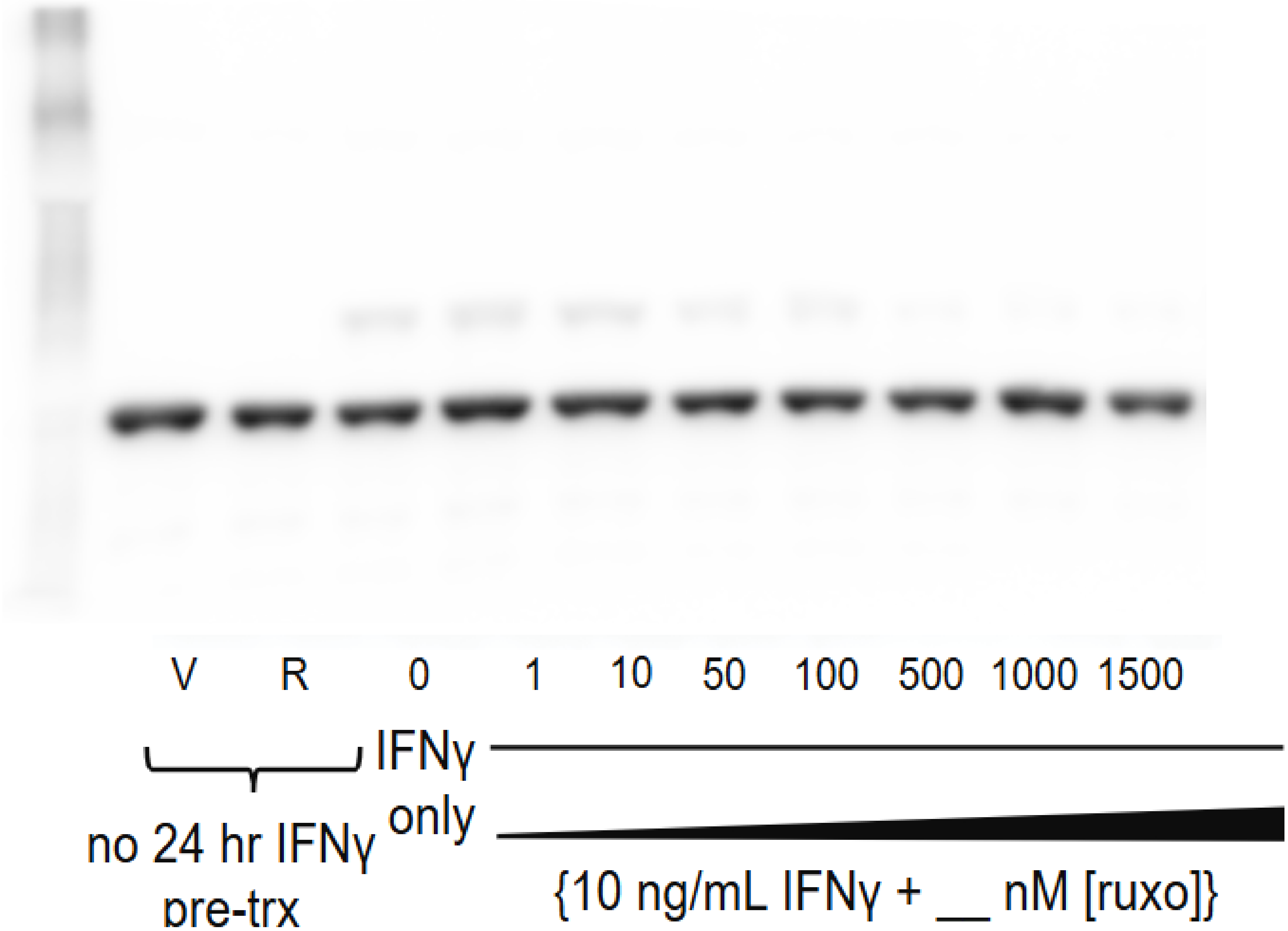

S1 Fig S5 Fig A, tSTAT3

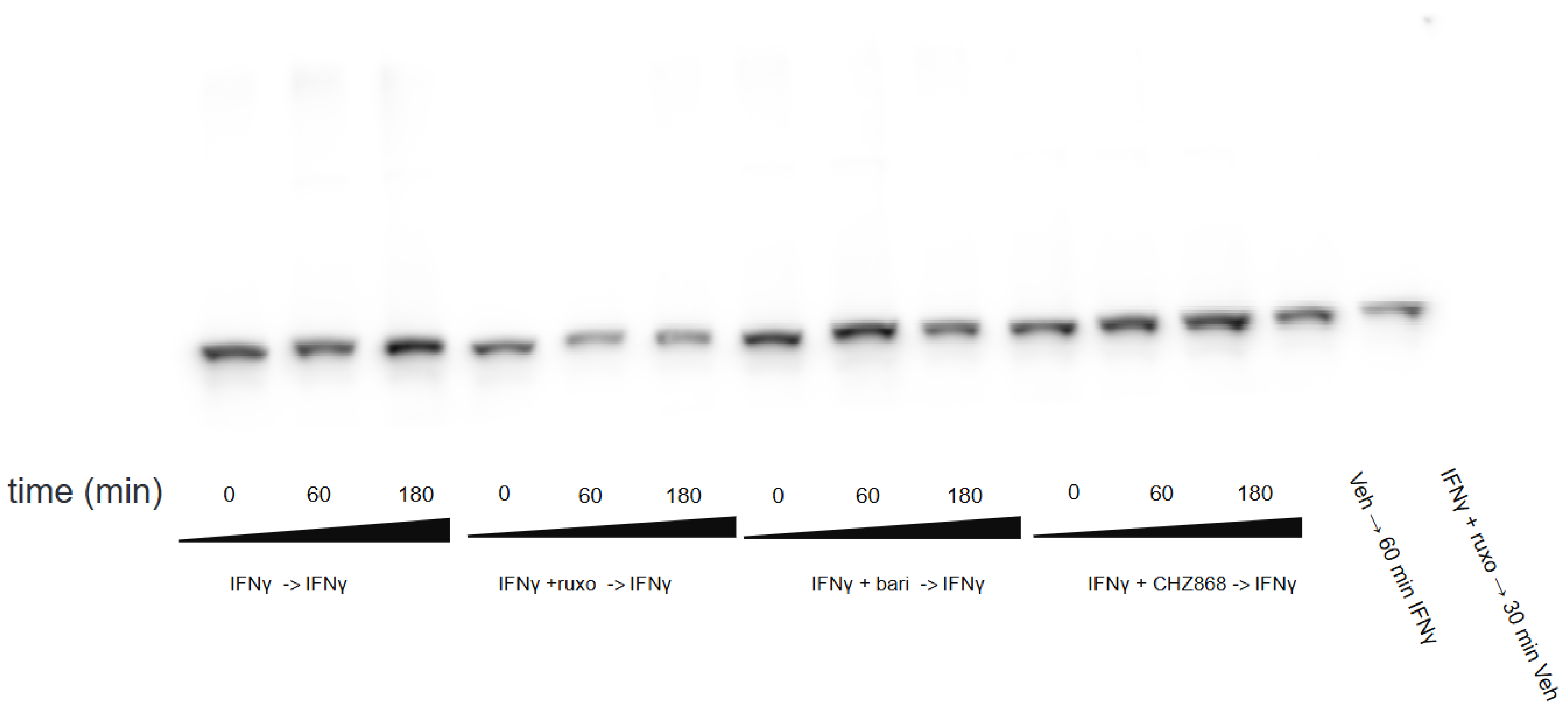

S1 Fig S5 Fig A, GAPDH for tSTAT3

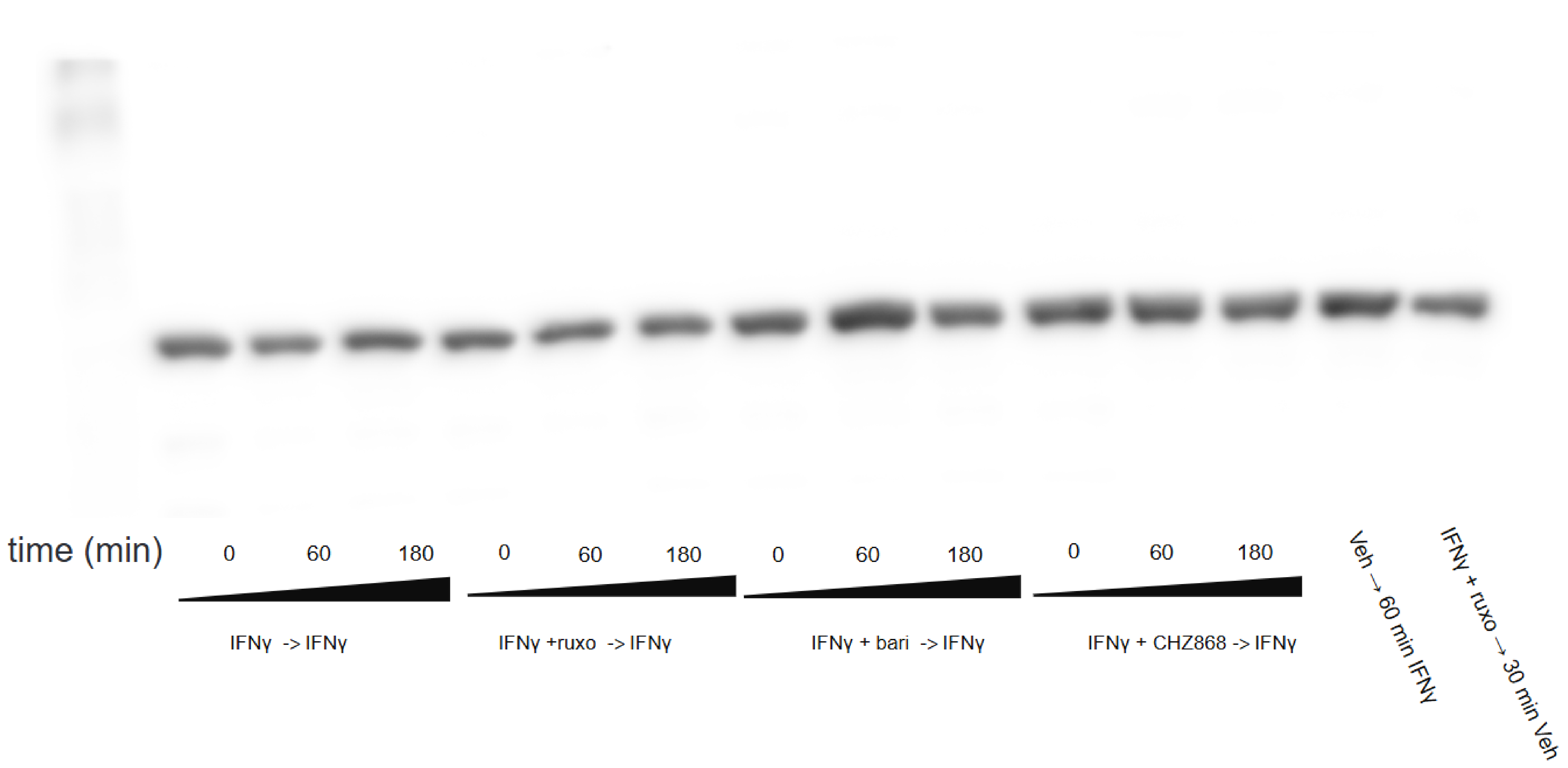

S1 Fig S5 Fig A, GAPDH for pSTAT3

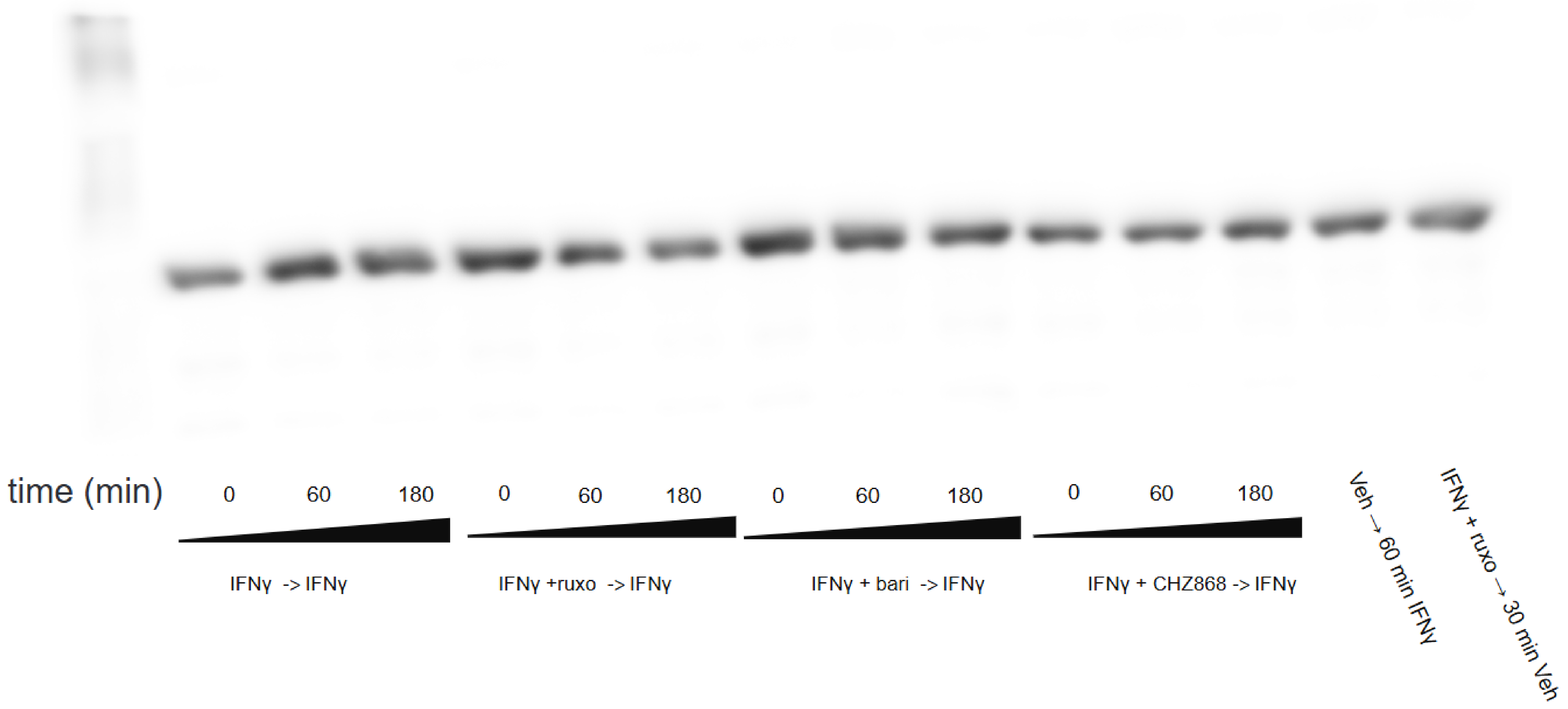

S1 Fig S5 Fig A, pSTAT3

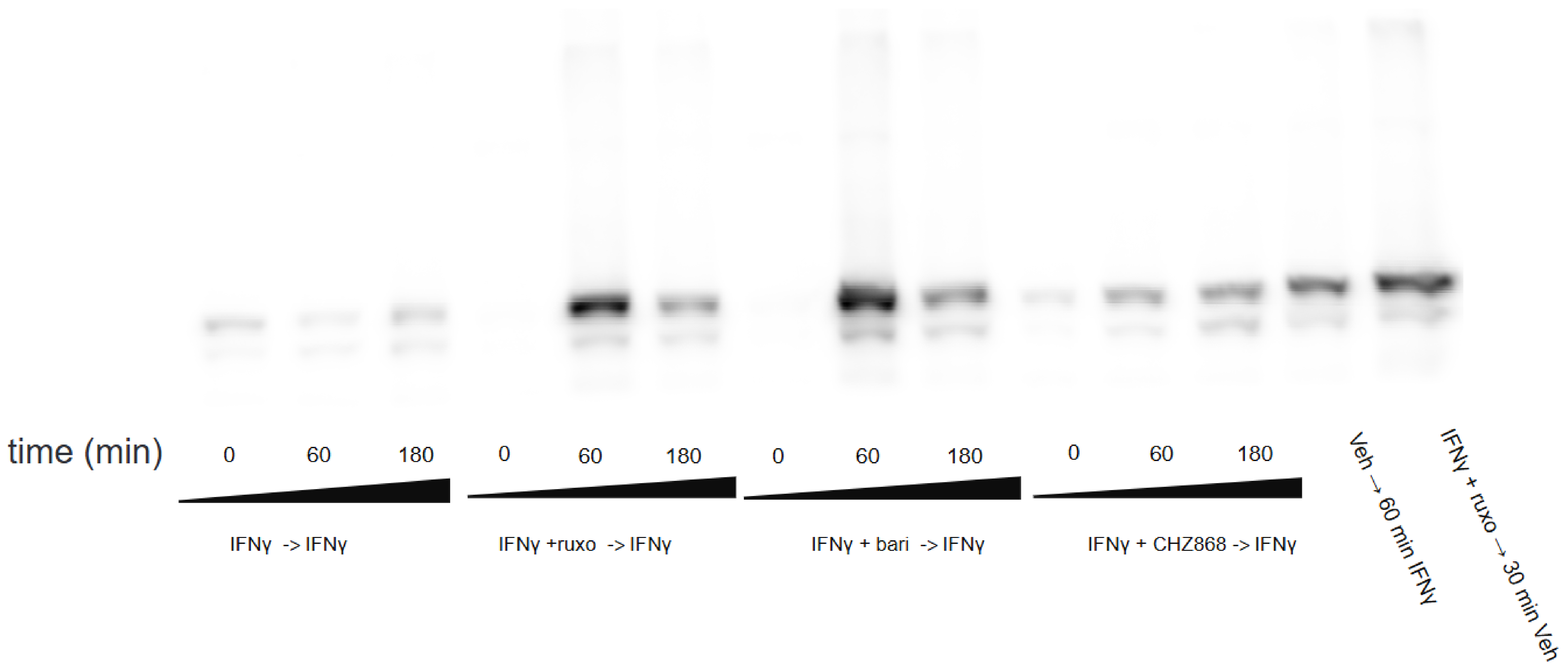

S1 Fig S5 Fig B, pSTAT4

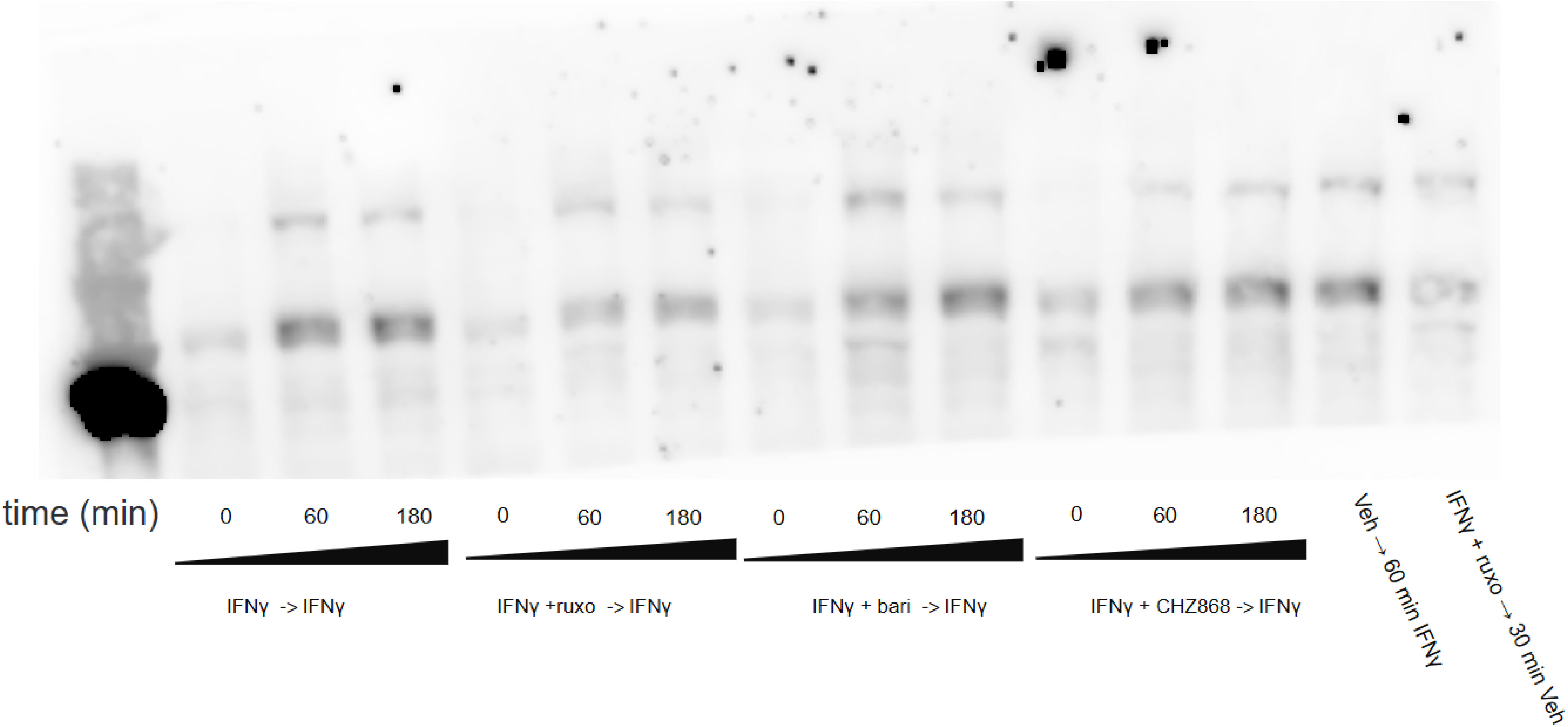

S1 Fig S5 Fig B, tSTAT4

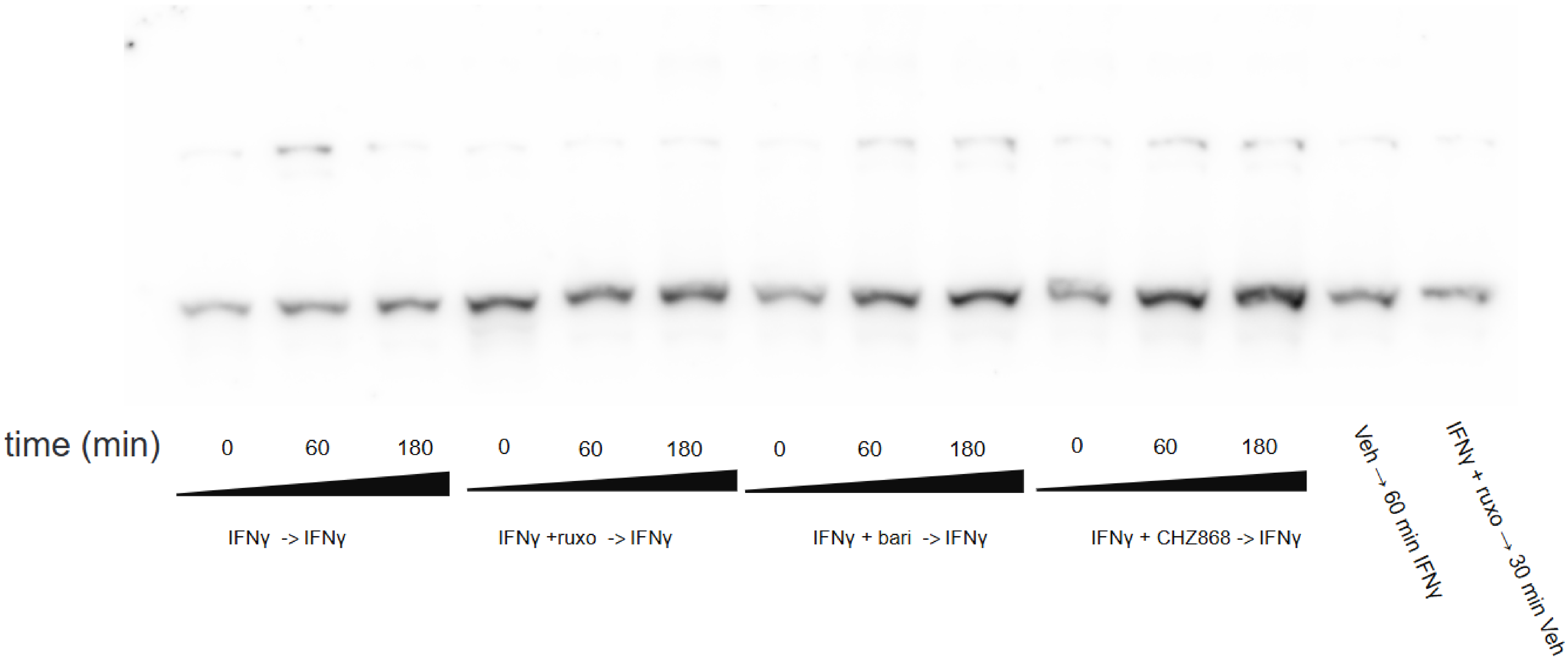

S5 Fig B, GAPDH

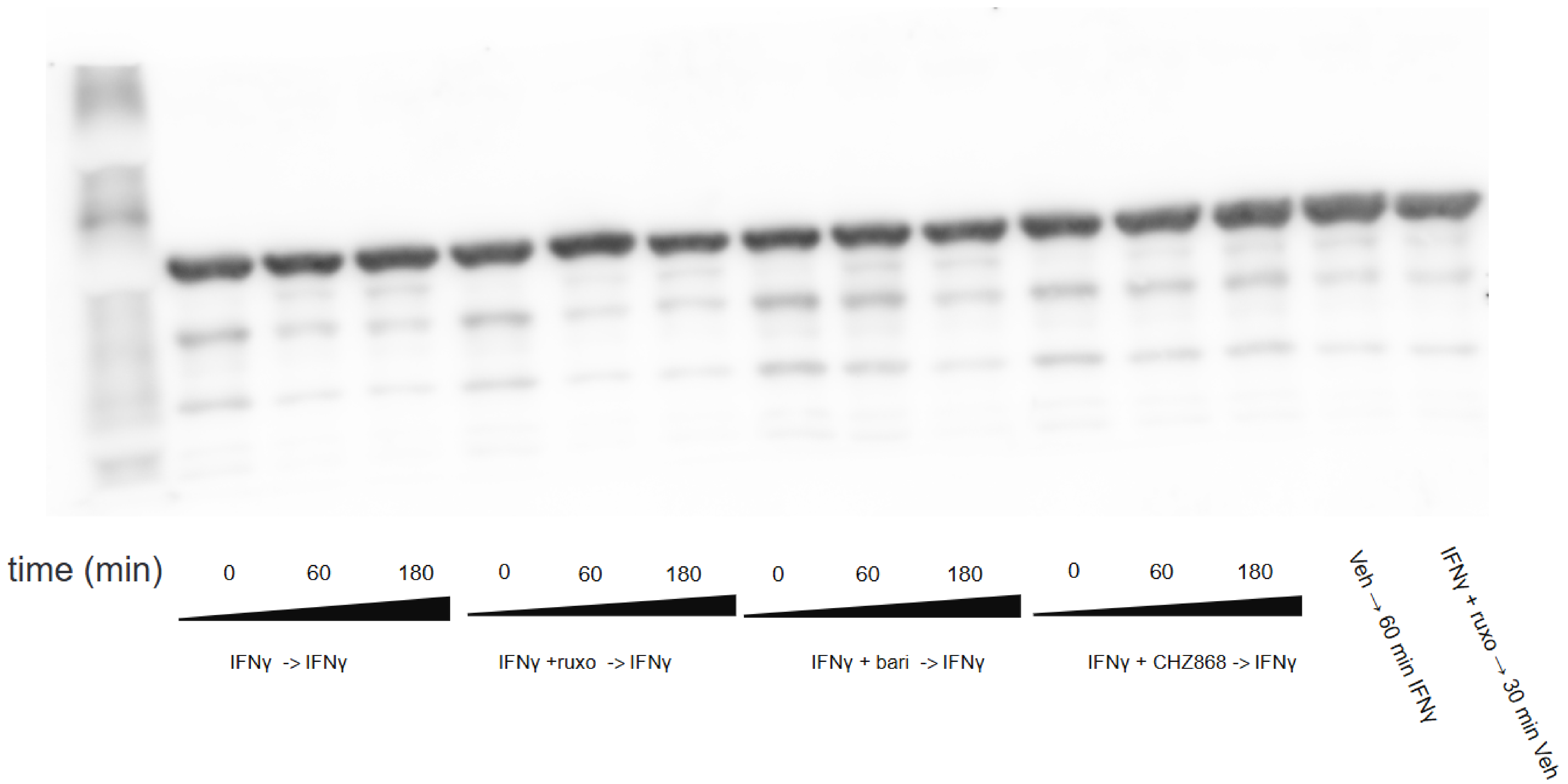

S1 Fig S5 Fig C, tSTAT5

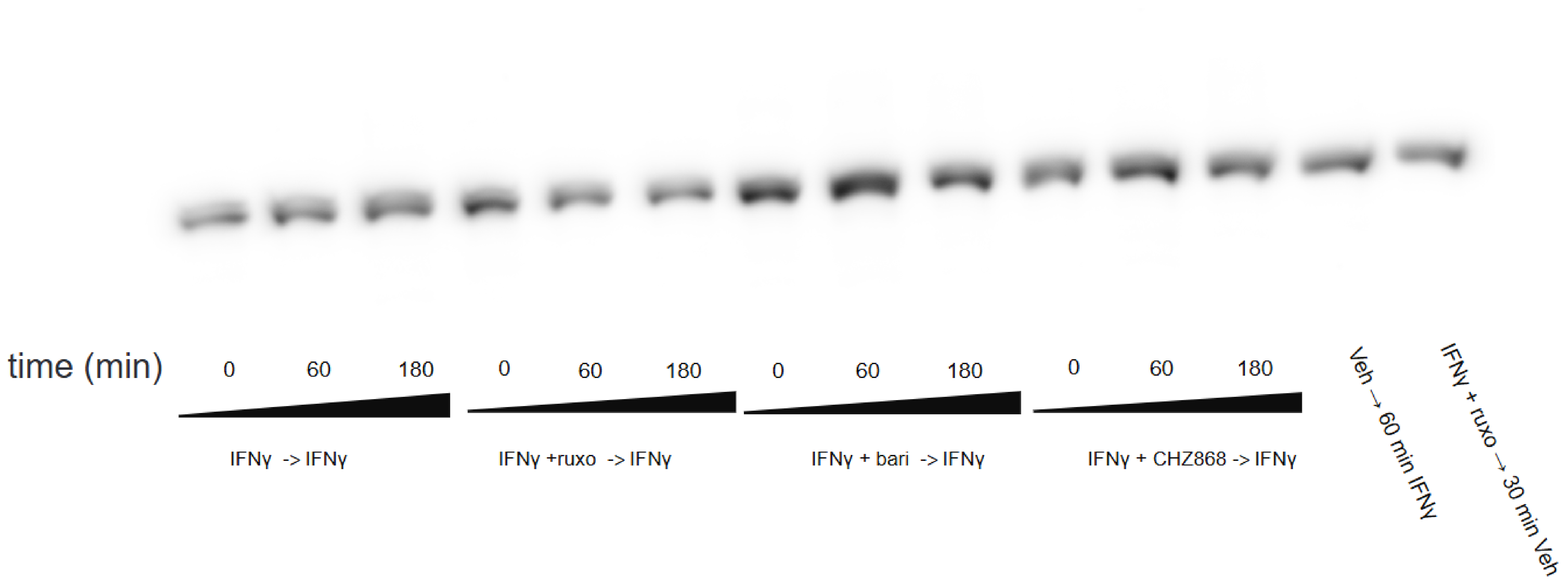

S1 Fig S5 Fig C, pSTAT5

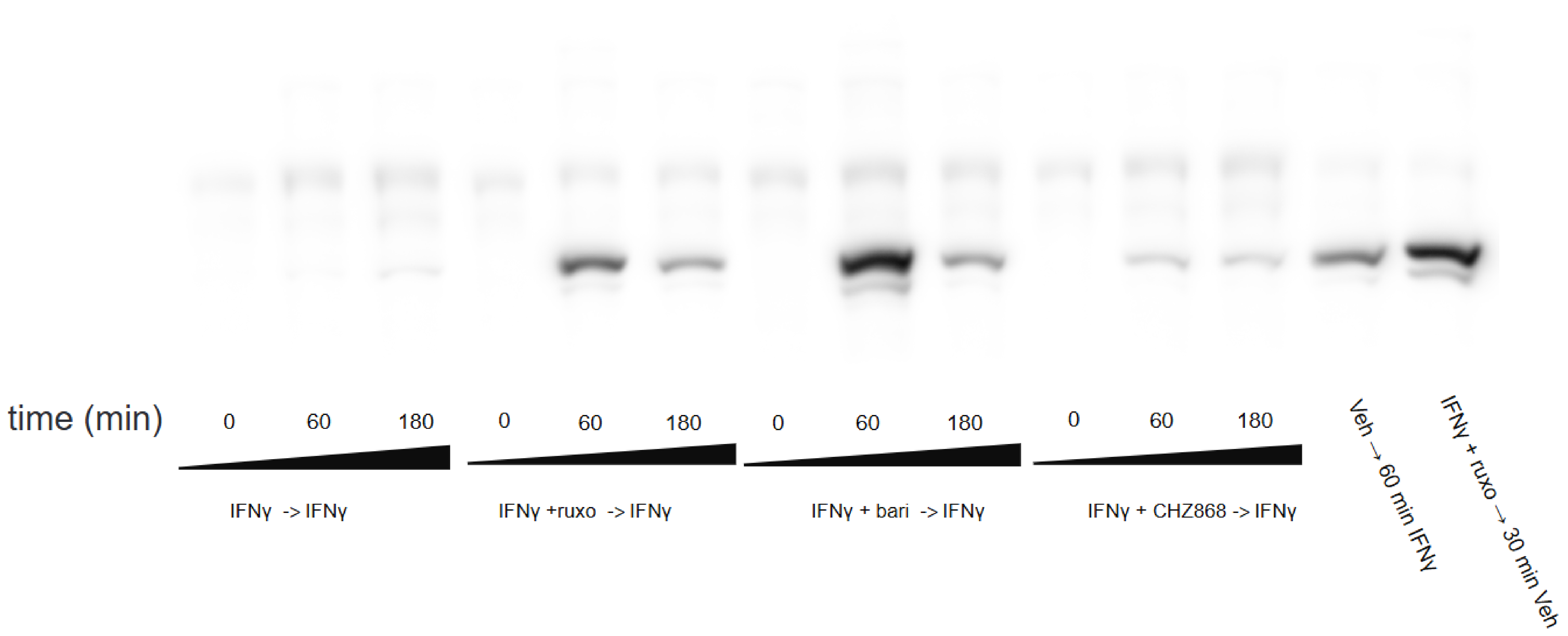

S1 Fig S5 Fig C, GAPDH

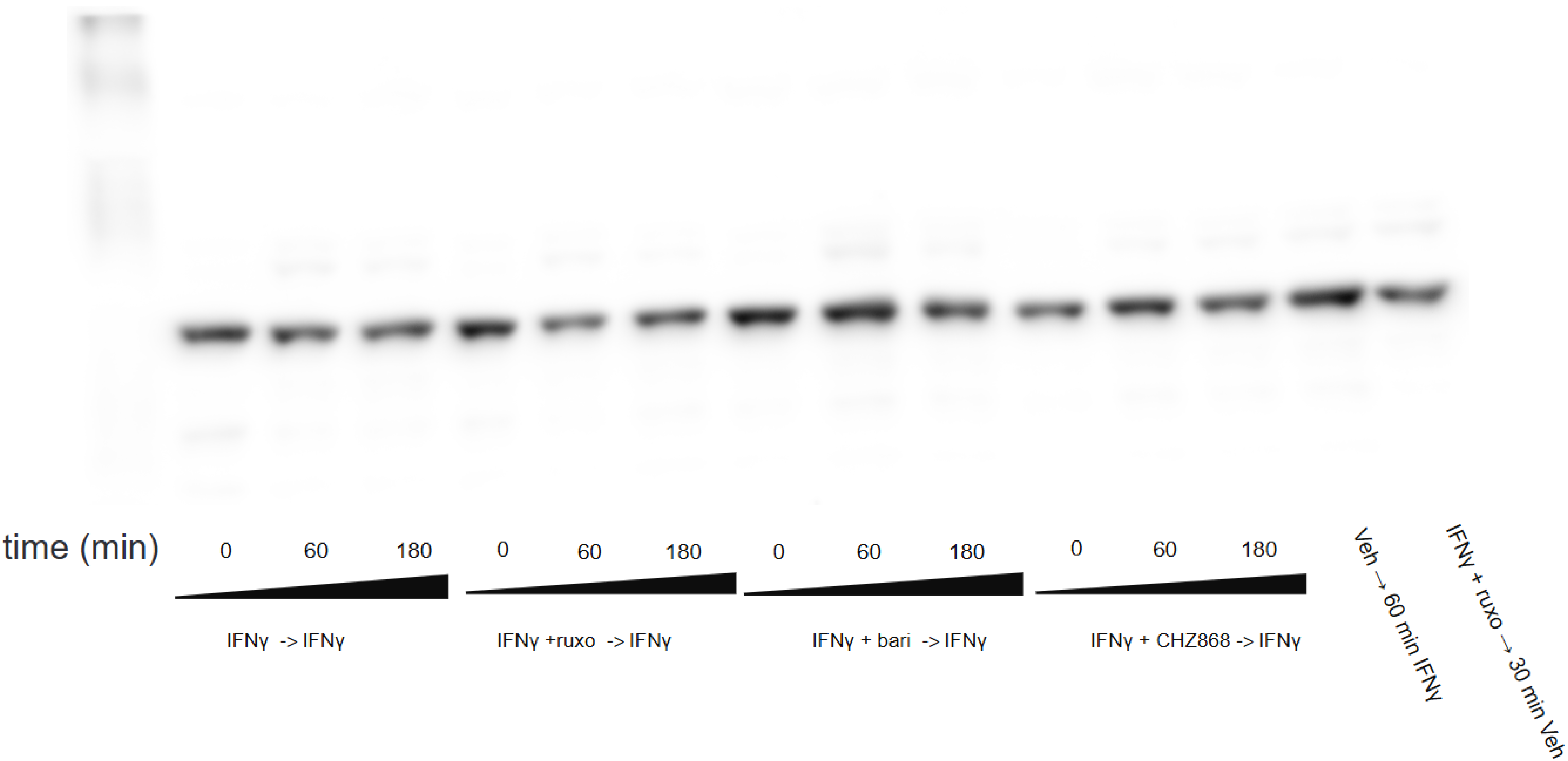

S1 Fig S5 Fig D, tERK

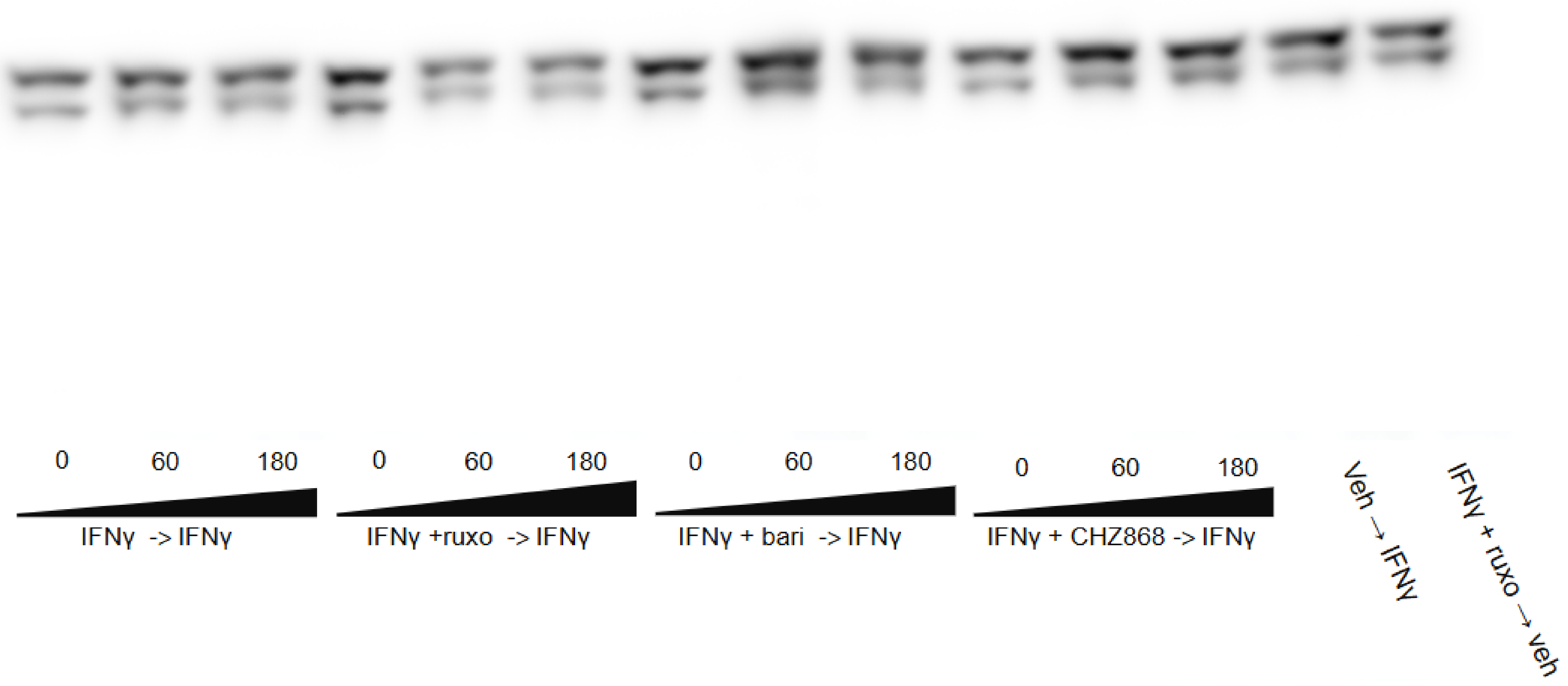

S1 Fig S5 Fig D, pERK

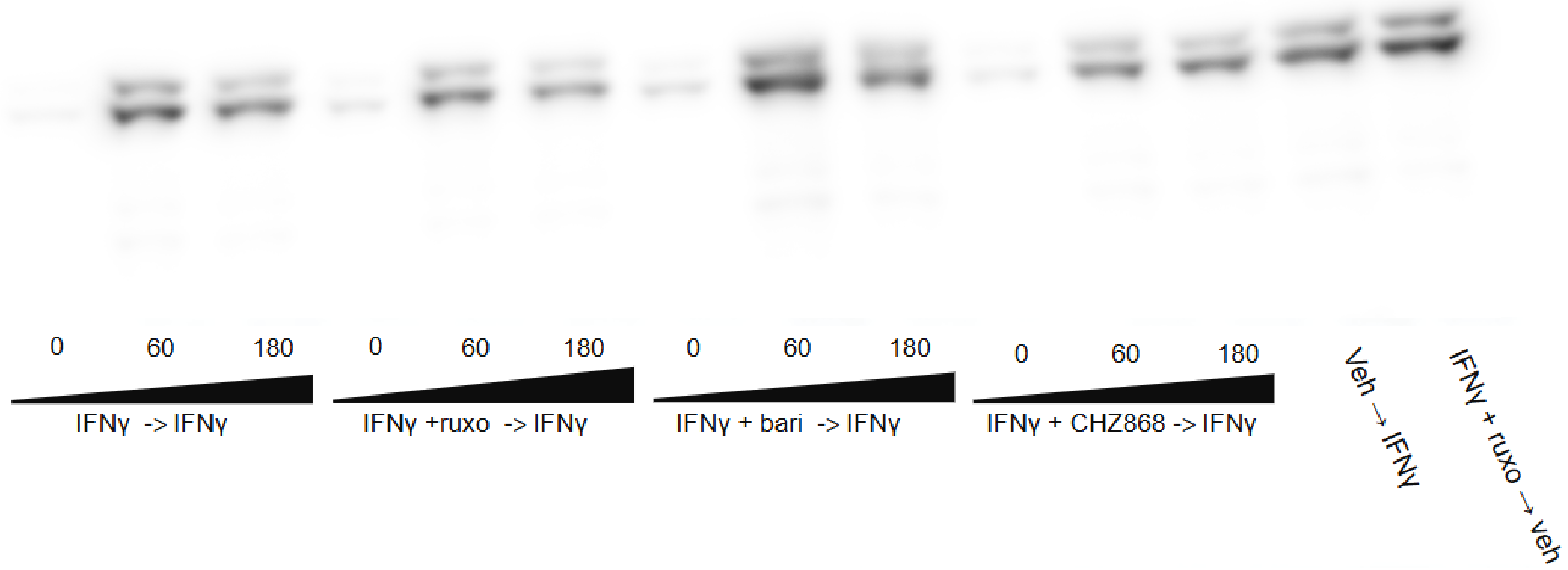

S1 Fig S5 Fig D, GAPDH

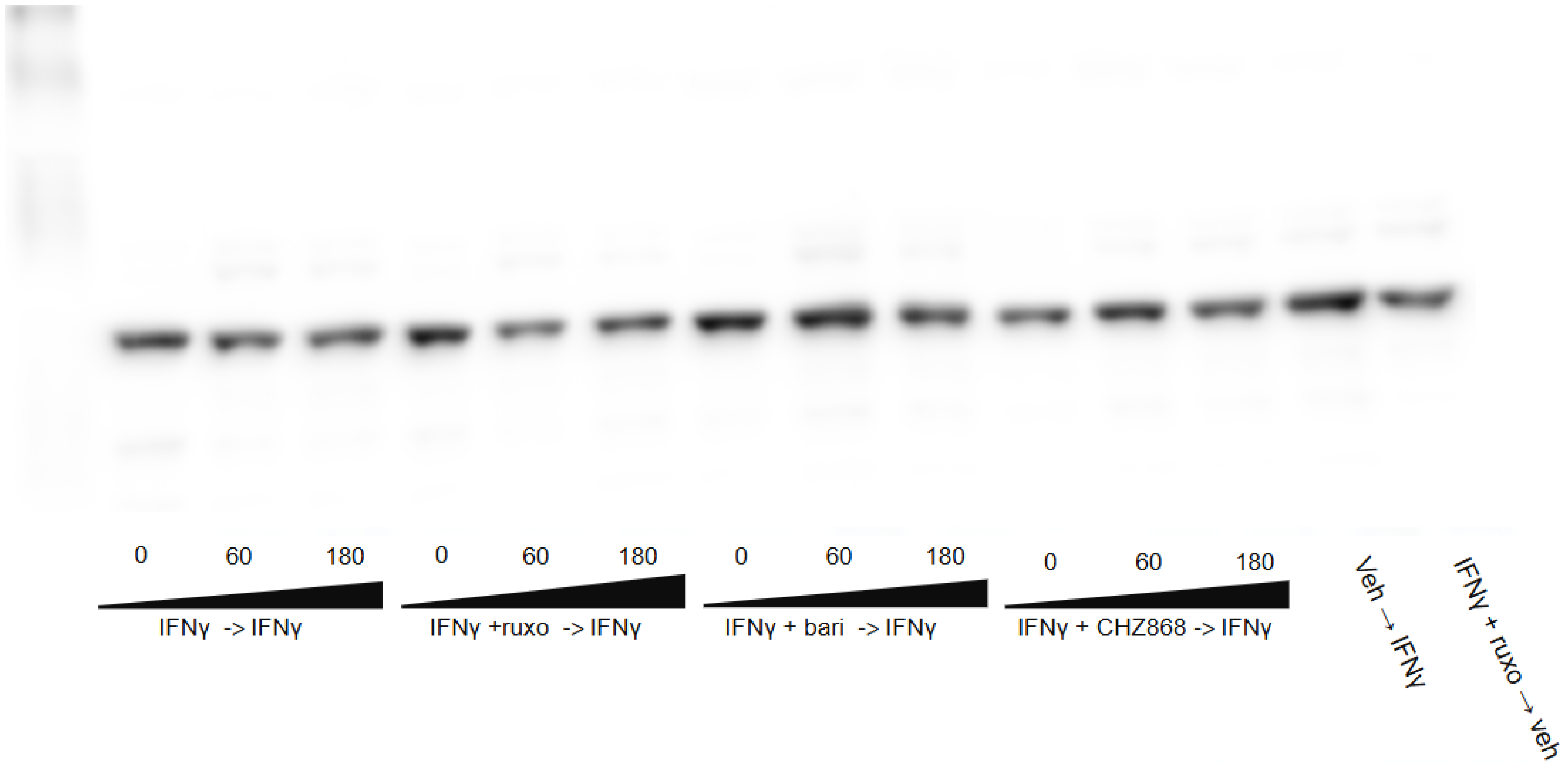

S1 Fig S5 Fig E, tAkt

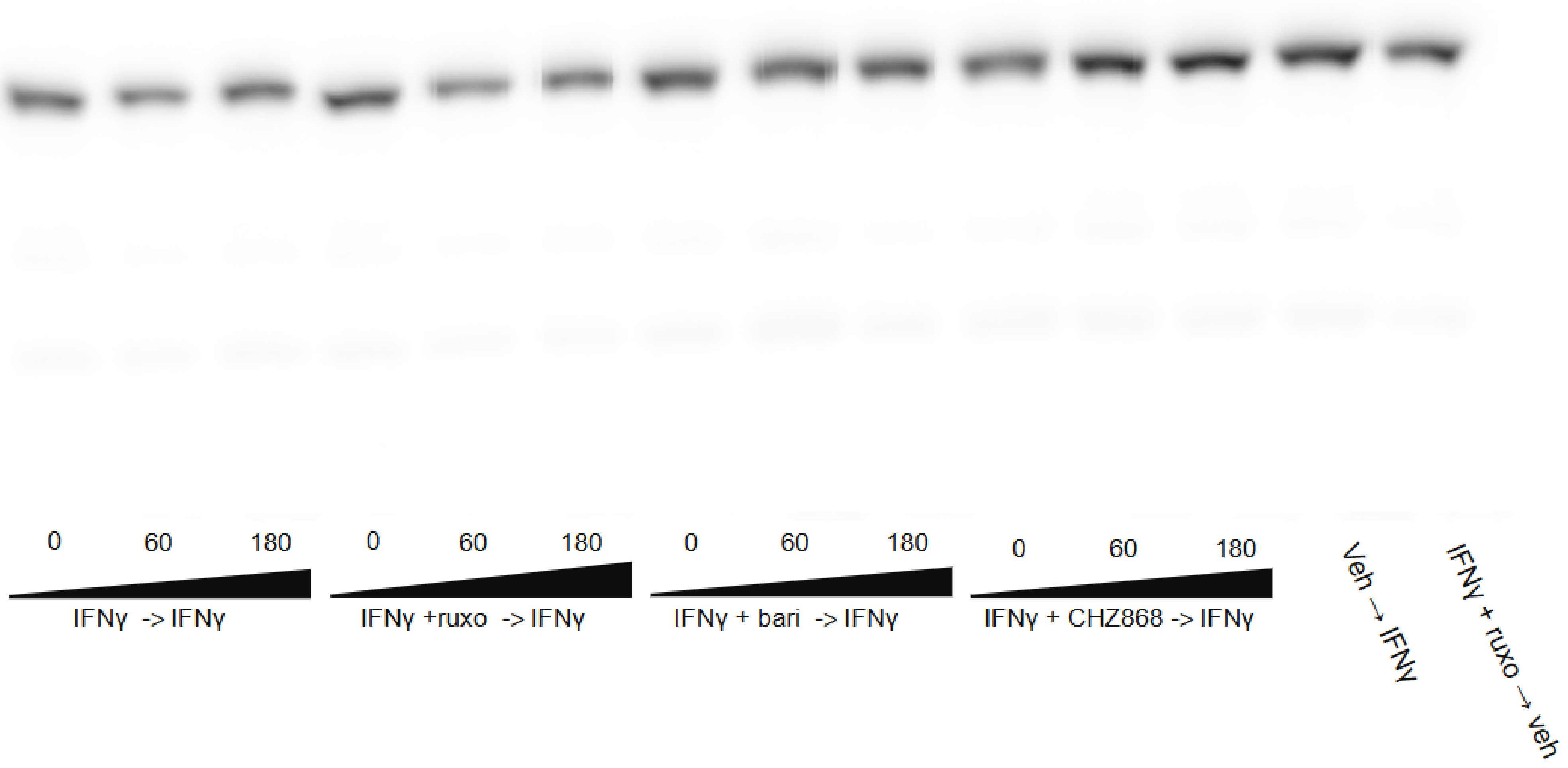

S1 Fig S5 Fig E, GAPDH for tAkt

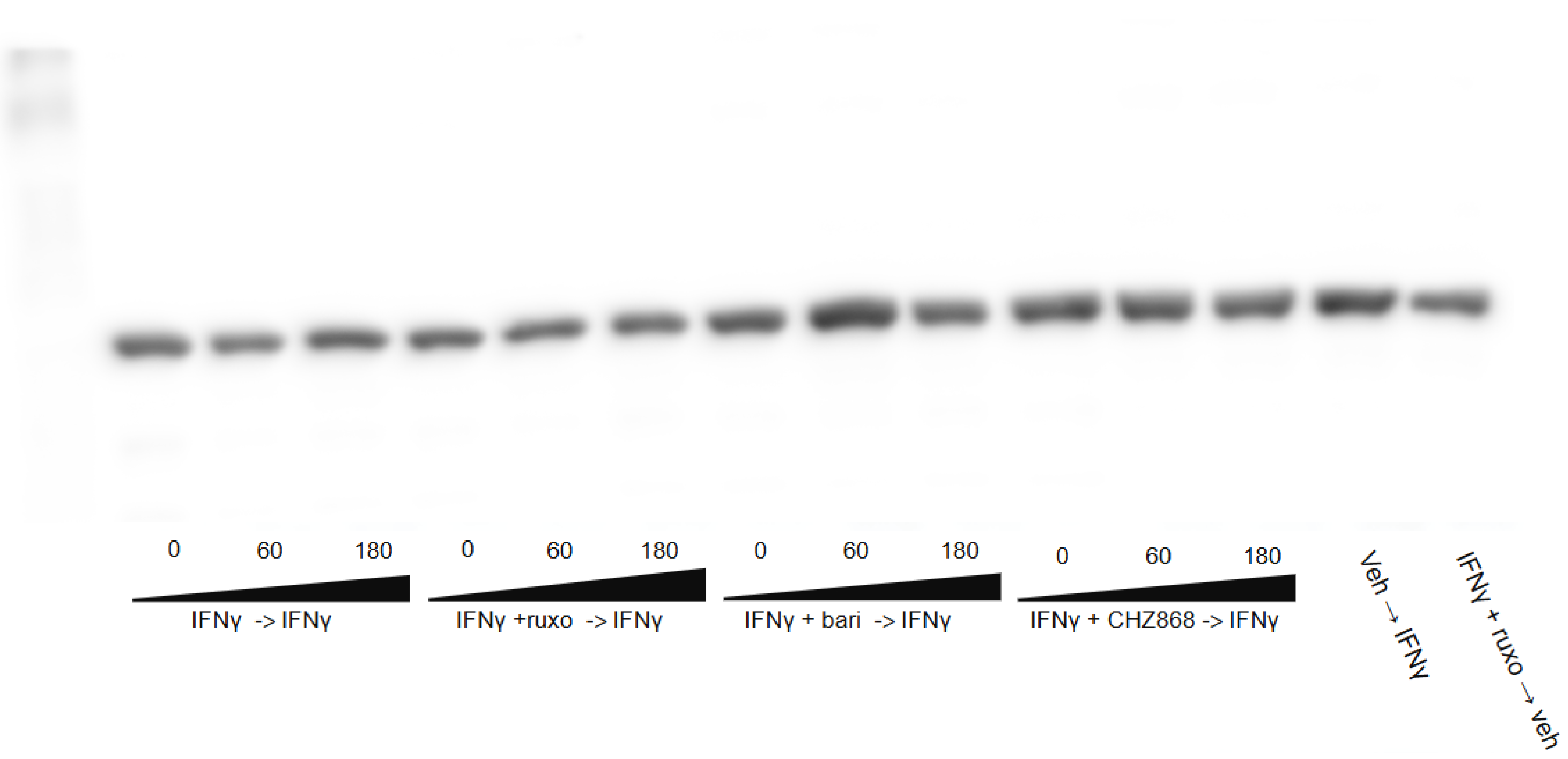

S1 Fig S5 Fig E, pAkt

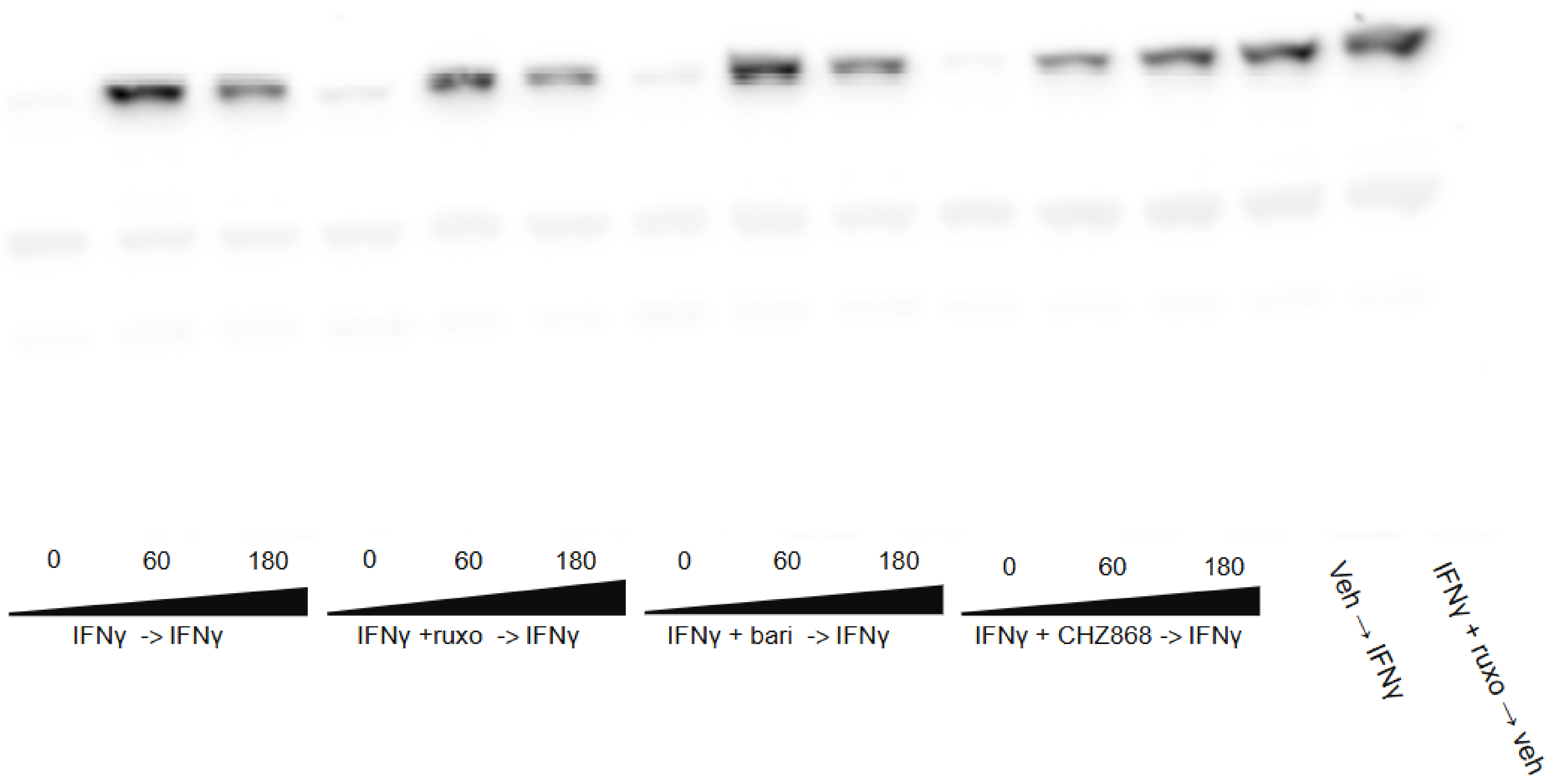

S1 Fig S5 Fig E, GAPDH for pAkt

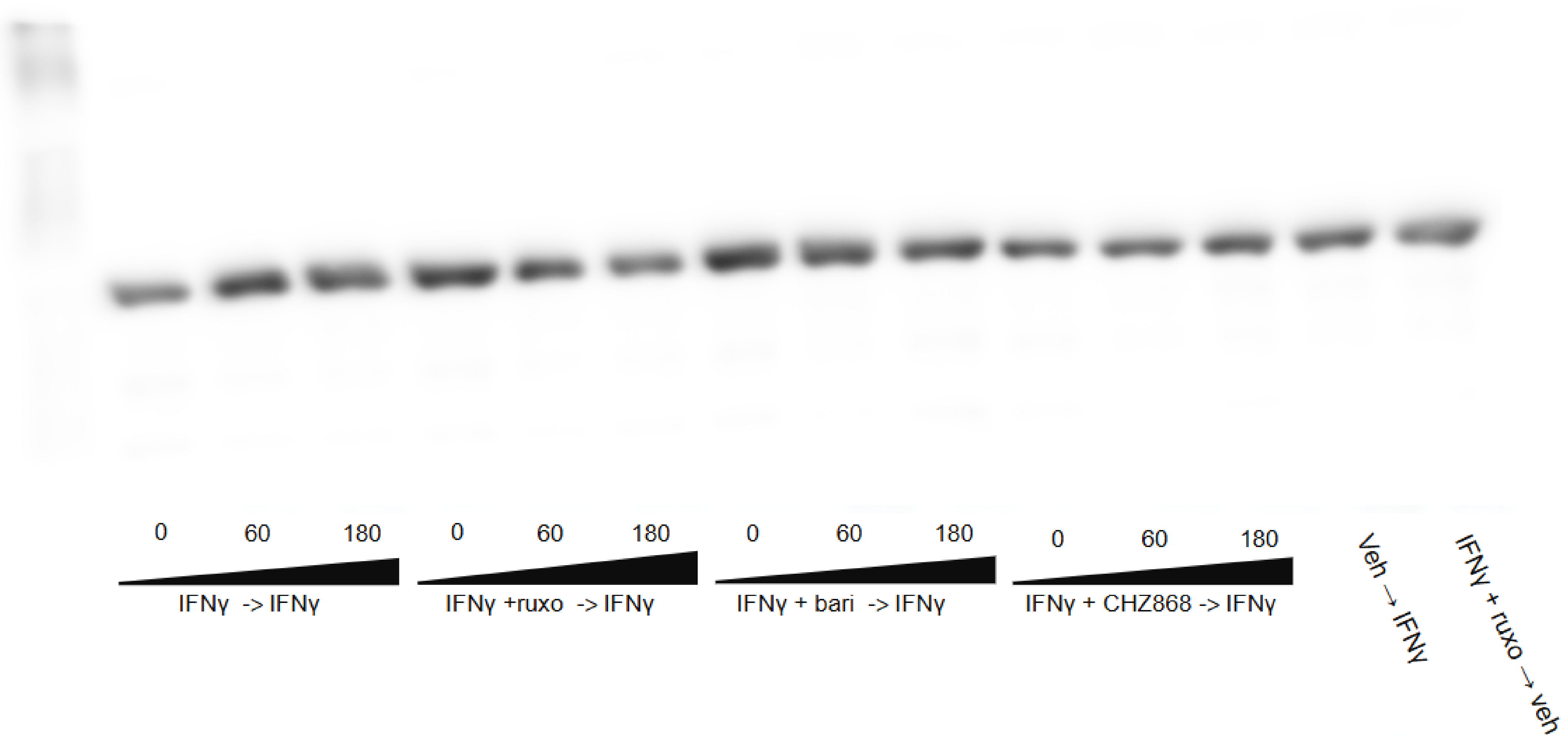

Supplement: S1 Fig — (PDF) [file pone.0311706.s001.pdf]
