# Supplementary figures and images for: JAK inhibitor withdrawal causes a transient pro-inflammatory cascade: A potential mechanism for major adverse cardiac events
Source: PLoS One. 2025 Jun 16;20(6):e0311706. doi: 10.1371/journal.pone.0311706 (PMC12169581; doi:10.1371/journal.pone.0311706)

A.

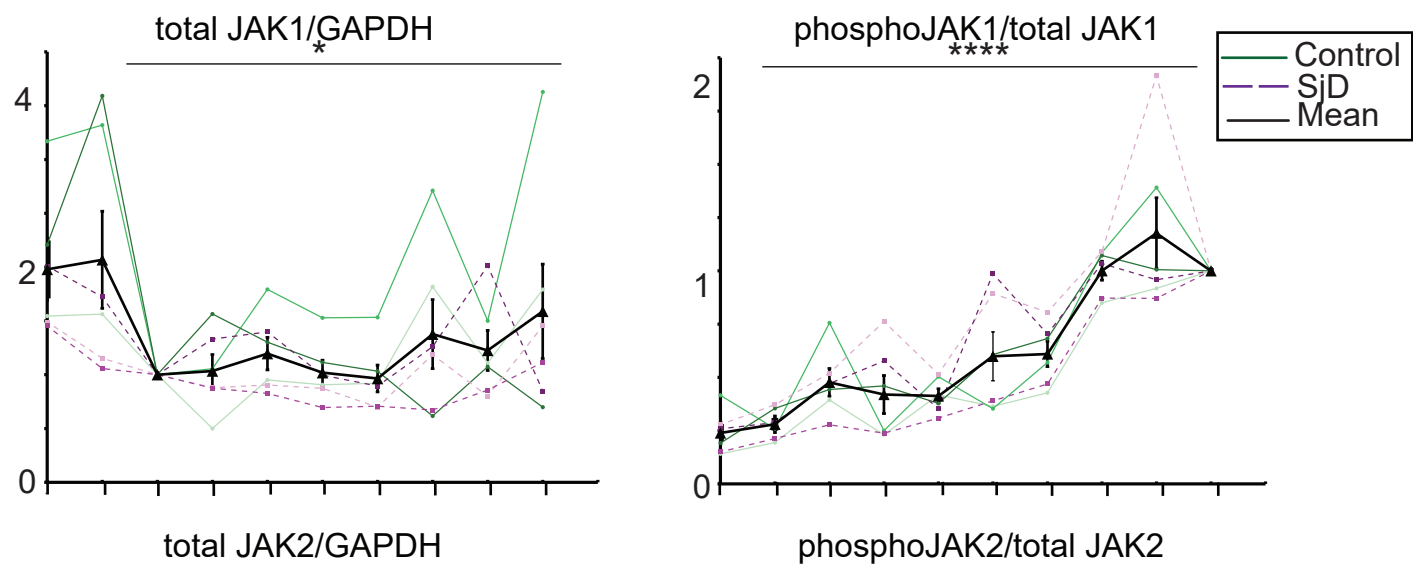

B.

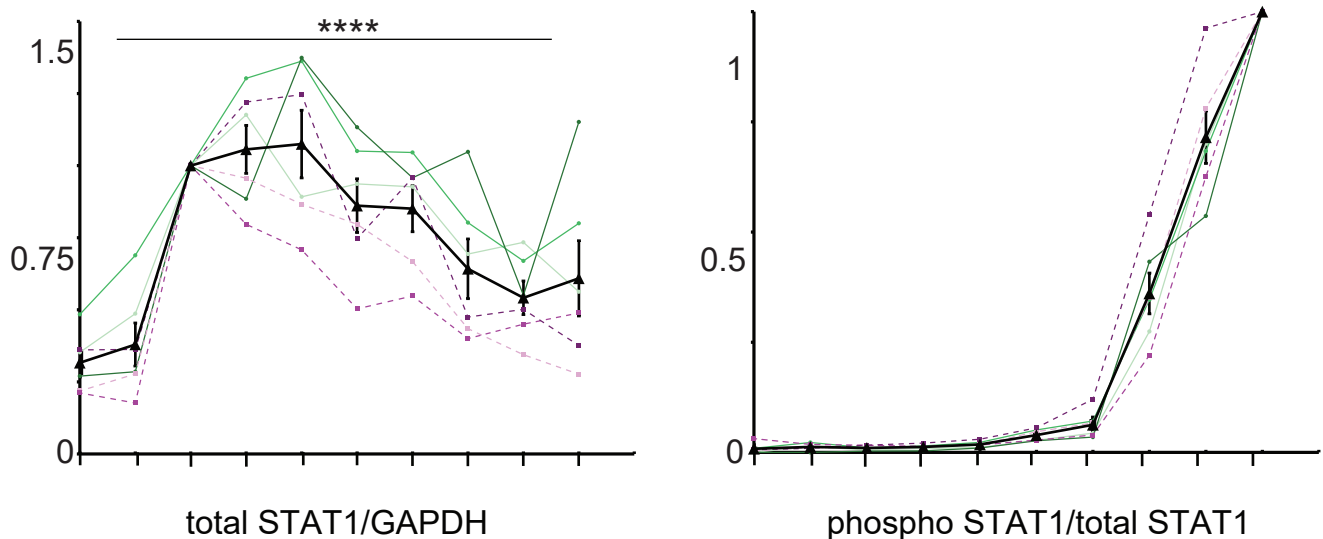

C.

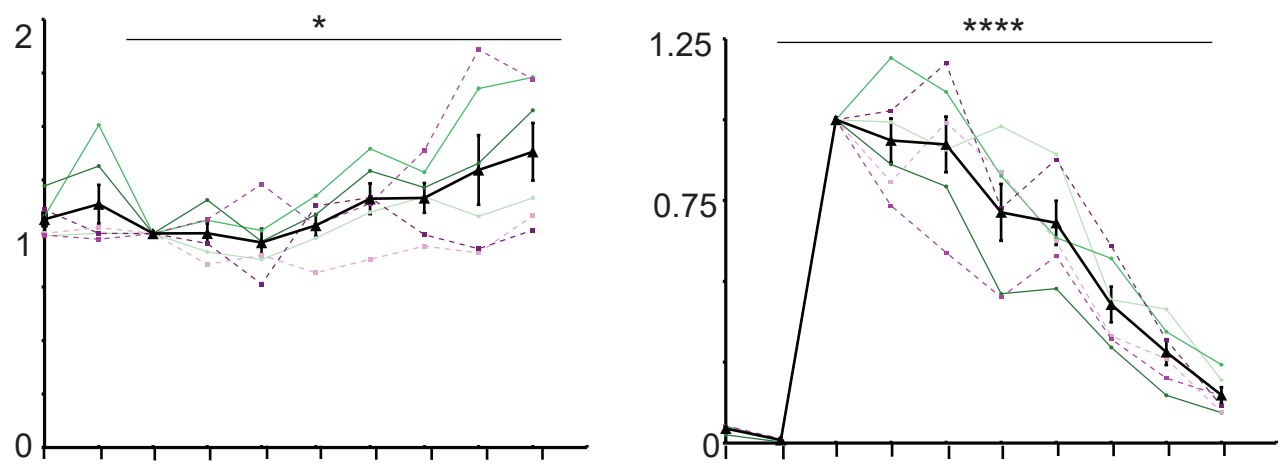

Supplement: S2 Fig — We treated MSCs with vehicle, ruxolitinib (1 µM), IFNγ only (10 ng/mL), IFNγ then switching to ruxolitinib only, or IFNγ 10 ng/mL in combination with ruxolitnib 1 uM for varying periods of time or for 48 hours at varying doses of ruxolitinib (0-1500nM). At each time or dose of ruxolitinib, we harvested cells for protein and performed western blot for the indicated target. (A) total JAK1 remains stable with increasing concentrations of ruxo whereas phosphor JAK1 increases with total ruxo concentration; (B) total JAK2 peaks at 50 nM ruxo, subsequently decreasing, whereas pJAK2 increases with ruxo concentration; (C) Total STAT1 remains stable with increasing rux, whereas pSTAT1 is inversely correlated with ruxo dose. There is no difference in any condition between SjD (n = 3) and control (n = 3) MSCs. * < 0.05; *** = p < 0.0001. (PDF) [file pone.0311706.s002.pdf]

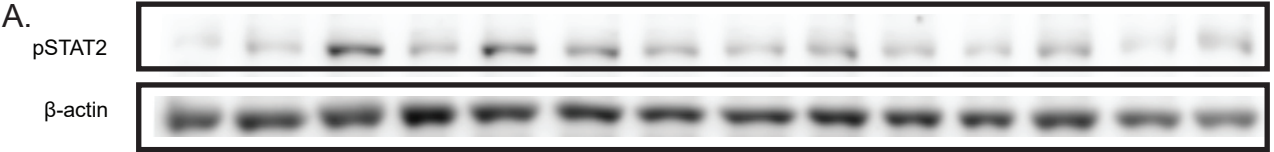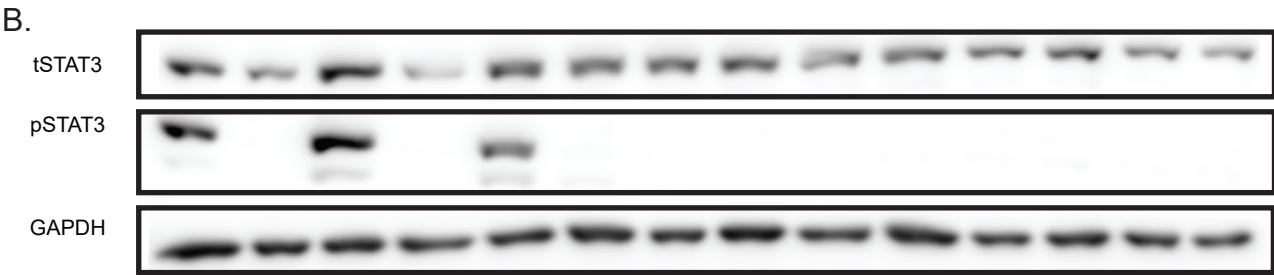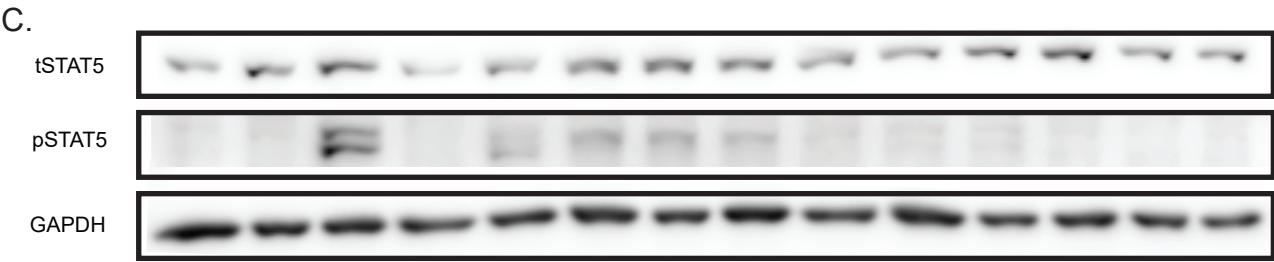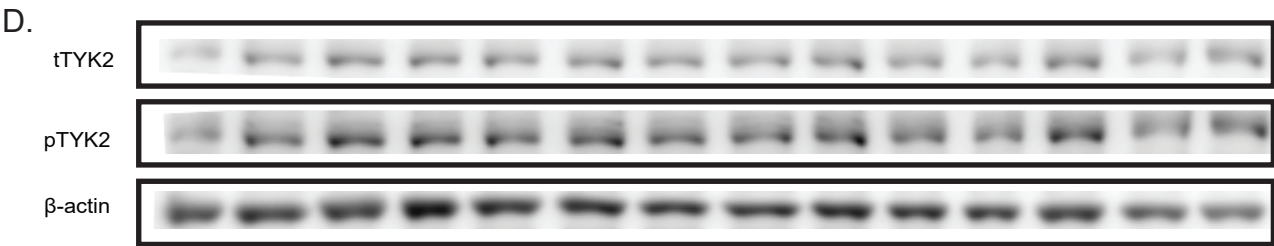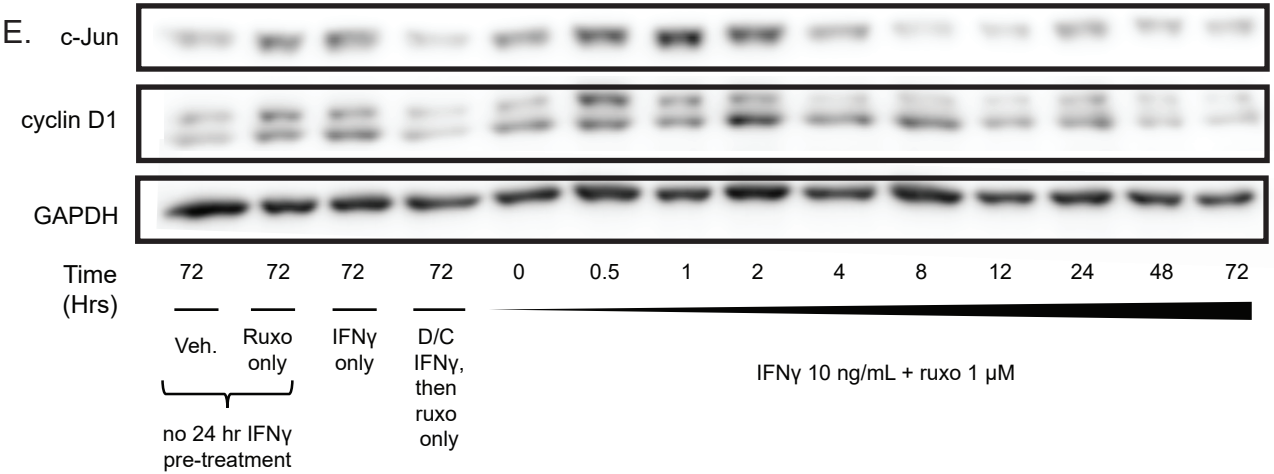

Supplement: S3 Fig — We treated MSCs with vehicle, ruxolitinib, IFNγ only, IFNγ then switching to ruxolitinib only, or IFN with ruxolitinib at varying times. Western blots with indicated antibodies are shown. Results demonstrate treatment of MSCs derived from a control subject. (PDF) [file pone.0311706.s003.pdf]

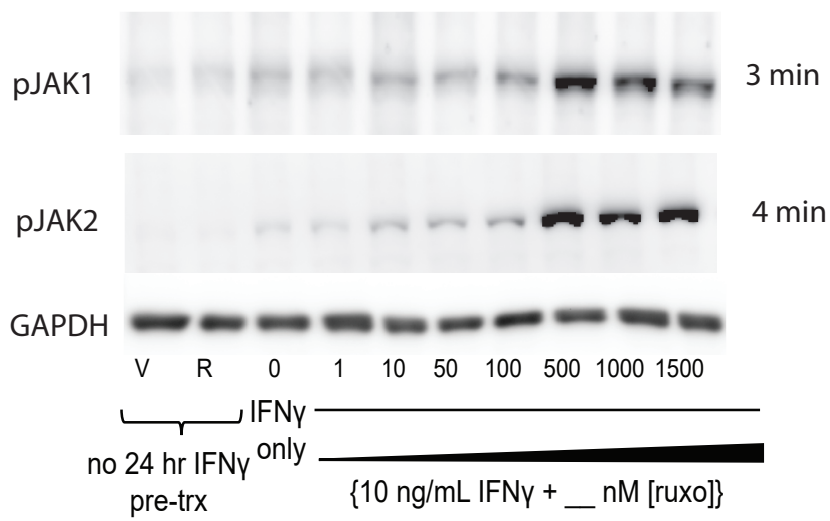

Supplement: S4 Fig — Control subject MSCs were treated with IFN gamma with ruxolitinib at stated doses. The results show western blots of total pJAK1 and pJAK2 with longer exposure times than those shown in Fig 2. (PDF) [file pone.0311706.s004.pdf]

A.

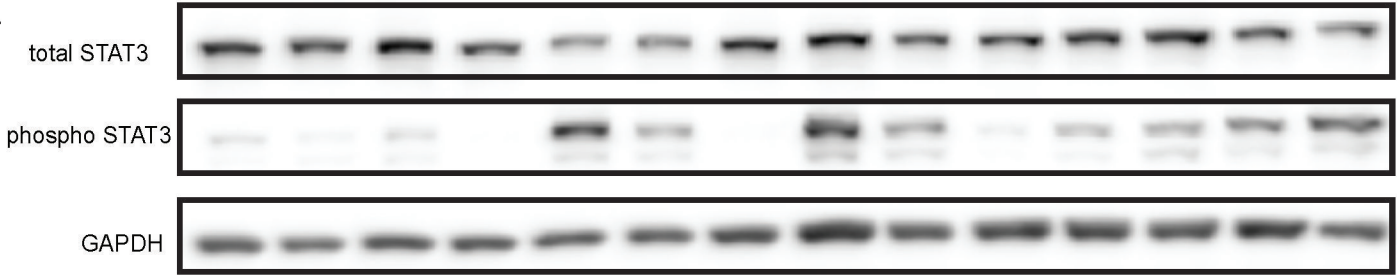

B.

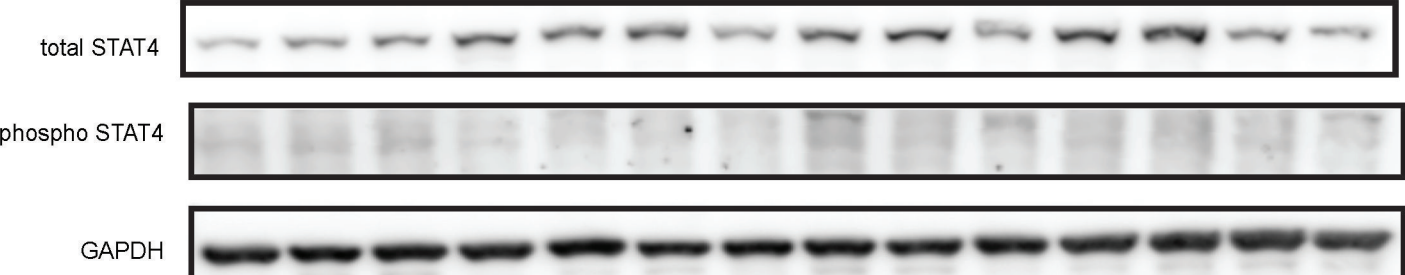

C.

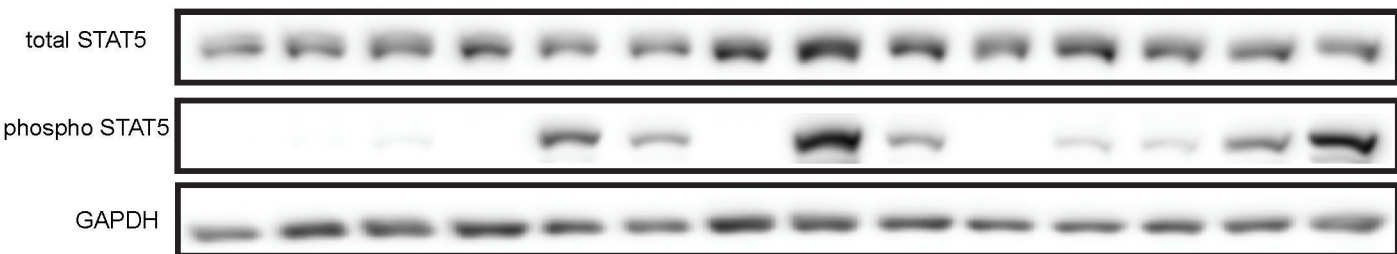

D.

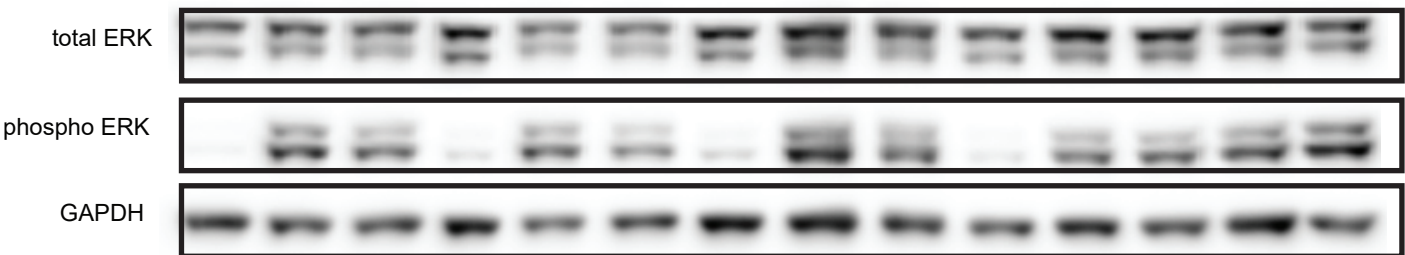

E.

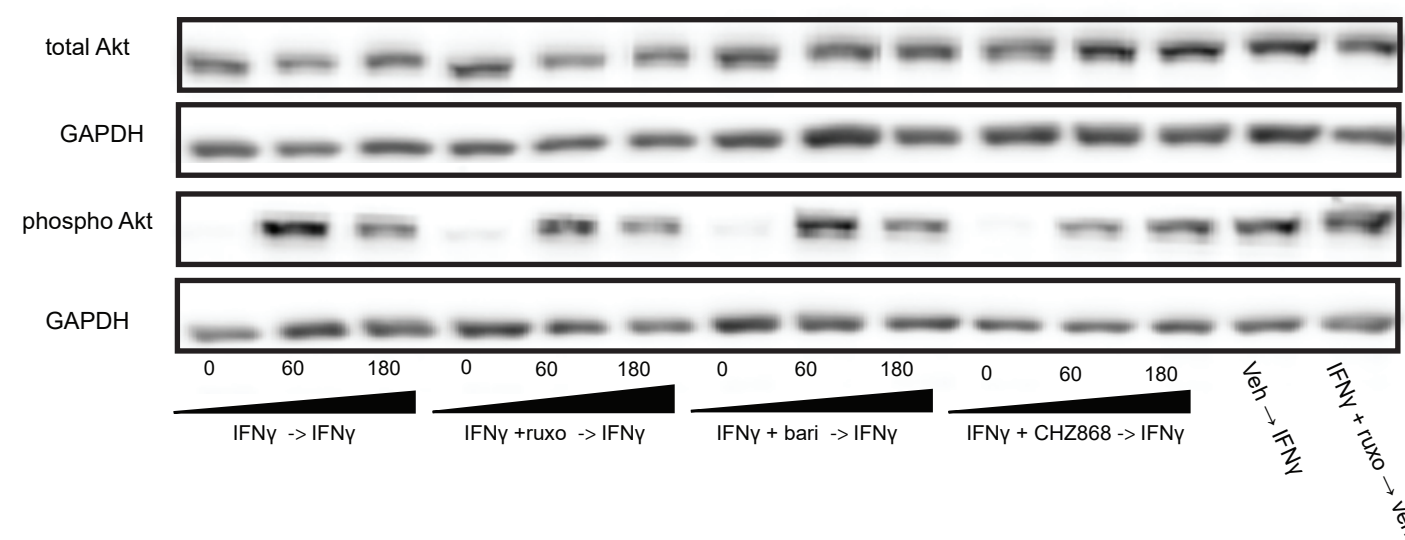

Supplement: S5 Fig — Salivary gland MSCs were treated with IFN gamma for 48 hours. Ruxolitinib, baricitinib, or CHZ868 were added for 24 hours. The cells were washed and replaced with media containing only IFNγ for variable periods of time. GAPDH is the control for each condition. Results demonstrate treatment of MSCs derived from a control subject. Six conditions are shown: 1) IFNγ to IFNγ as a control; 2) ruxolitinib withdrawal; 3) baricitinib withdrawal; 4) CHZ868 withdrawal; 5) vehicle to IFNγ; 6) IFNγ and ruxolitinib to vehicle. The results show western blots of total STAT3–5, pSTAT3–5, total ERK, phosphoERK, total AKT, and phosphoAKT. (PDF) [file pone.0311706.s005.pdf]

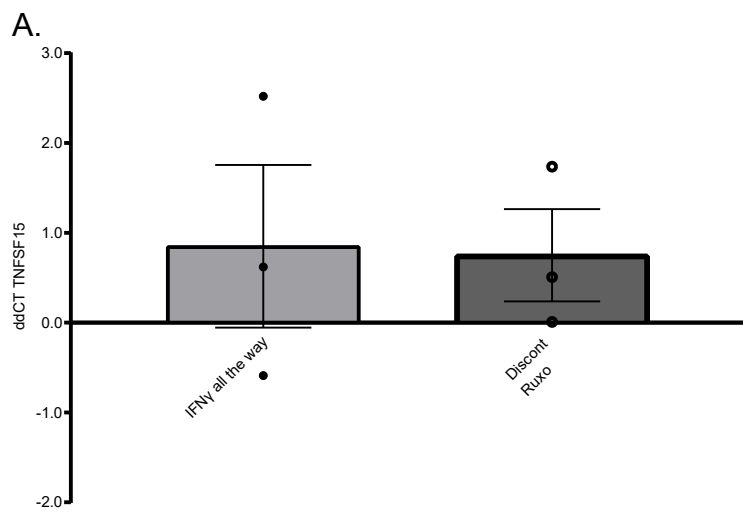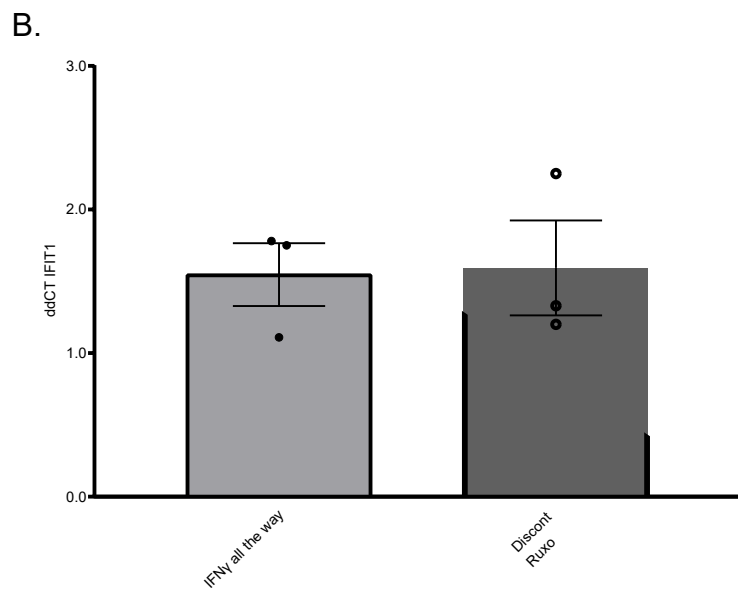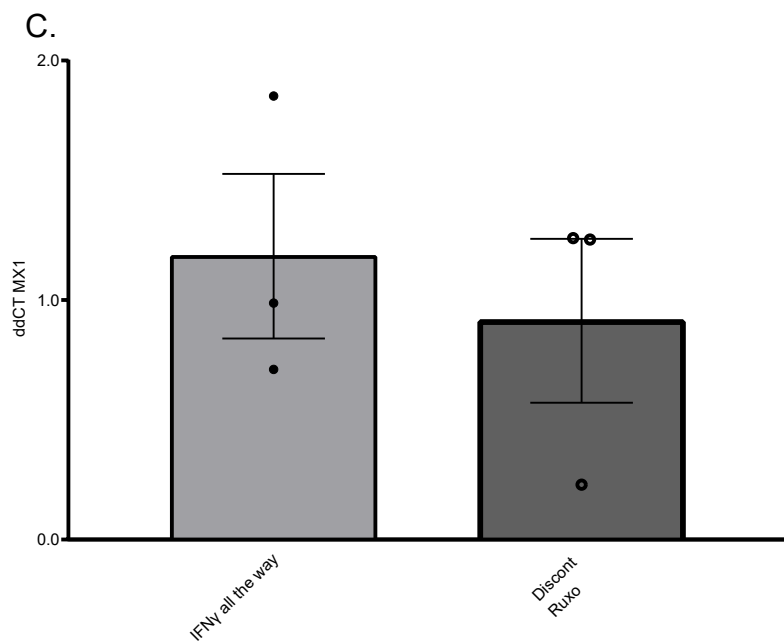

Supplement: S6 Fig. — Ruxolitinib withdrawal increases inflammatory transcripts more than CHZ868. qPCR was performed after MSCs were exposed to the following treatments: IFNγ 10 ng/mL and ruxolitinib (1000 nM), wash cells, then continue treatment only with IFNγ (ruxolitinib withdrawal), or IFNγ and CHZ868 (1000 nM) treatment, wash cells, then continue treatment with only IFNγ (CHZ868 withdrawal). Panels A-C show ddCT of the displayed transcript in the demonstrated condition compared to discontinuation of CHZ868. Unpaired t-testing was performed between delta CT values of the displayed conditions compared to discontinuation of CHZ868. No results achieved significant difference thresholds. (PDF) [file pone.0311706.s006.pdf]
