# Supplementary material for: JAK inhibitor withdrawal causes a transient pro-inflammatory cascade: A potential mechanism for major adverse cardiac events
Source: PLoS One. 2025 Jun 16;20(6):e0311706. doi: 10.1371/journal.pone.0311706 (PMC12169581; doi:10.1371/journal.pone.0311706)
Supplement: S1 Table — (PDF) [file pone.0311706.s007.pdf]

Supplemental Table S1: Characteristics of the patients from which MSCs were derived

Age	Sex	SjD	Figure(s)
39	F	control	5
60	F	SjD	1
52	F	control	1
52	F	control	5
51	F	control	5
30	F	SjD	1
31	F	SjD	1,2,3
31	F	control	1
49	F	control	1

SjD patients met 2016 ACR/EULAR criteria. Control subjects were referred for labial salivary gland biopsy due to dryness symptoms but did not have a defined autoimmune disease.
