# Supplementary material for: JAK inhibitor withdrawal causes a transient pro-inflammatory cascade: A potential mechanism for major adverse cardiac events
Source: PLoS One. 2025 Jun 16;20(6):e0311706. doi: 10.1371/journal.pone.0311706 (PMC12169581; doi:10.1371/journal.pone.0311706)
Supplement: S2 Table — (PDF) [file pone.0311706.s008.pdf]

Supplemental Table S2: Characteristics of the patients from which NK cells were derived

	MTX, n=9 (%)	JAKi, n=16 (%)
Age, mean ± SD	65.2 ± 14.5	57.8 ± 16.6
Women	8 (88.9)	14 (87.5)
**Rheumatoid arthritis**		
Disease duration (years), mean ± SD	4.2 ± 4.2	14.9 ± 8.4
Erosive	3/8 (37.5)	11 (68.8)
Rheumatoid Factor positivity	6/8 (75)	9/15 (60)
ACPA positivity	6/8 (75)	11/15 (73.3)
DAS28, mean ± SD	2.1 ± 1.6	3 ± 1.1
**Treatments**		
Corticosteroids	4 (44.4)	4 (25)
Average corticosteroids dose, mean ± SD	7 ± 3.6	6.2 ± 2.5
MTX	9 (100)	9 (56.2)
Baricitinib	NA	10 (62.5)
Tofacitinib	NA	6 (37.5)
Prior bDMARD	0	16 (100)

Data are n (%) unless otherwise indicated
