# Supplementary material for: JAK inhibitor withdrawal causes a transient pro-inflammatory cascade: A potential mechanism for major adverse cardiac events
Source: PLoS One. 2025 Jun 16;20(6):e0311706. doi: 10.1371/journal.pone.0311706 (PMC12169581; doi:10.1371/journal.pone.0311706)
Supplement: S3 Table — (PDF) [file pone.0311706.s009.pdf]

SUPPLEMENTAL TABLE S3. PRIMER SEQUENCES AND TARGETS

TARGET	Forward	Reverse
**B-ACTIN**	5’-CATCACGATGCCAGTGGTACG-3’	5’-AACCGCGAGAAGATGACCCAG-3’
**IFIT1**	5'-GCCCTGGAGTACTATGAGCGG-3'	5'-GCTGATATCTGGGTGCCTAAGGAC-3'
**MX1**	5'-GGCTGTTTACCAGACTCCGACA-3'	5'-CACAAAGCCTGGCAGCTCTCTA-3'
**MX2**	5'-TGAACGTGCAGCGAGCTT-3'	5'-GGCTTGTGGGCCTTAGACAT-3'
**PLAT**	5’-AGCGAGCCAAGGTGTTTCAAC-3’	5’-TGCCCCTGTAGCTGATGCC-3’
**PLAU**	5’-GCTTCTCTGCGTCCTGGTC-3’	5’-TGGGCAGTTGCACCAGTGA-3’
**TNFSF15**	5'-CACCACATACCTGCTTGTCAGC-3'	5'-TCTCCGTCTGCTCTAAGAGGTG-3'
