# Supplementary material for: JAK inhibitor withdrawal causes a transient pro-inflammatory cascade: A potential mechanism for major adverse cardiac events
Source: PLoS One. 2025 Jun 16;20(6):e0311706. doi: 10.1371/journal.pone.0311706 (PMC12169581; doi:10.1371/journal.pone.0311706)
Supplement: S4 Table — (PDF) [file pone.0311706.s010.pdf]

Supplemental Table S4: Fluorochrome-labelled antibodies used for flow cytometry

Target	Fluorochrome	Manufacturer	Reference
CD3	BV510	Biolegend	317332
CD56	PE-Cy7	Beckman Coulter	A21692
IFNγ	FITC	BD Bioscience	552887
TNF	APC	BD Bioscience	551384
CD107a	FITC	Biolegend	328606
