# Supplementary material for: JAK inhibitor withdrawal causes a transient pro-inflammatory cascade: A potential mechanism for major adverse cardiac events
Source: PLoS One. 2025 Jun 16;20(6):e0311706. doi: 10.1371/journal.pone.0311706 (PMC12169581; doi:10.1371/journal.pone.0311706)
Supplement: S5 Table — (PDF) [file pone.0311706.s011.pdf]

SUPPLEMENTAL TABLE S5. DEG AFTER RUXOLITINIB WITHDRAWAL VS. CHZ868 WITHDRAWAL

GENE_NAME	S1DCruxo_Raw.Read	S2-DCruxo_Raw.Read	S3-DCruxo_Raw.Read	S1DCCHZ_Raw.Read	S2DCCHZ_Raw.Read	S3DCCHZ_Raw.Read	S1DCruxo_Normalized	S2DCruxo_Normalized	S3DCruxo_Normalized	S1DCCHZ_Normalized	S2DCCHZ_Normalized	S3DCCHZ_Normalized	baseMean	log2FoldChange	lfcSE	stat	pvalue	padj
**RGS20**	1025	595	721	241	186	171	982	622	721	233	174	179	485	-1.98664094472105	0.287212657295507	-6.91696864416752	4.61410162897261e-12	7.08864433259063e-08
**RFPL4B**	104	81	95	19	8	9	100	85	95	18	7	9	52	-2.98288436026147	0.480902509025193	-6.20267997001698	5.55096481007842e-10	3.42214334285477e-06
**C3AR1**	127	78	79	17	14	13	122	82	79	16	13	14	54	-2.70859434239529	0.438750571747184	-6.17342635386018	6.68256852734772e-10	3.42214334285477e-06
**MX2**	8413	10588	6103	2947	2255	2229	8061	11070	6102	2855	2112	2333	5422	-1.78919027337591	0.295998811772291	-6.04458599905569	1.49794138080208e-09	5.75321835831559e-06
**RGS4**	5224	8283	4196	1554	1726	1000	5005	8660	4195	1505	1617	1047	3672	-2.09884312440017	0.356660590155835	-5.88470714828103	3.98759420639508e-09	1.22522819585695e-05
**FGF5**	5586	4755	4613	2313	1419	1733	5352	4971	4612	2241	1329	1814	3387	-1.4720610414076	0.260464861490997	-5.65166845531861	1.58897856449558e-08	4.06857961439093e-05
**RPL22L1**	784	638	872	365	343	336	751	667	872	354	321	352	553	-1.15775693776248	0.209546559044143	-5.52505821638705	3.29376376616826e-08	7.22887039137757e-05
**YPEL2**	789	853	770	258	409	328	756	892	770	250	383	343	566	-1.30788467112275	0.253186323745858	-5.16570031024098	2.39540337152074e-07	0.00037490116853608
**CRYBG3**	2820	1860	2378	1146	1205	892	2702	1945	2377	1110	1129	934	1699	-1.14654284930255	0.22102353382321	-5.16222737779334	2.44028619759214e-07	0.00037490116853608
**KAT6B**	332	336	319	678	646	597	318	351	319	657	605	625	479	0.93186317359861	0.180576682187871	5.16781184621046	2.36850567864606e-07	0.00037490116853608
**NIPAL1**	435	128	234	58	42	25	417	134	234	56	39	26	151	-2.68751464853356	0.523139041192437	-5.13728557212565	2.78735226054612e-07	0.000389291752534273
**CRYBG3**	1315	912	1134	573	603	487	1260	953	1134	555	565	510	829	-1.0383263180105	0.205064405267981	-5.06341564570211	4.11810644008478e-07	0.000527220576991854
**IFIT1**	9729	11747	4621	2802	2086	1536	9322	12281	4620	2714	1954	1608	5417	-2.06281360571809	0.409546508371783	-5.03682381	4.73320077080308e-07	0.000559355103398829

																256071		
**NET1**	3769	3067	3164	1942	1901	1391	3611	3206	3163	1881	1781	1456	2517	-0.963480261047032	0.196317588854696	-4.90776331691884	9.2120929586432e-07	0.00101089560088311
**NCOA7**	6278	4116	4918	2567	2358	2556	6015	4303	4917	2487	2209	2675	3768	-1.04757059945568	0.218939430408352	-4.78475072992479	1.7119966595304e-06	0.00175342697502711
**PLAU**	13447	22173	21052	3751	6247	7176	12884	23181	21047	3634	5852	7511	12352	-1.74854335278979	0.367480464258141	-4.75819403439501	1.9533268977662e-06	0.0017564043550494
**AQP3**	302	175	316	104	86	60	289	183	316	101	81	63	172	-1.69039490612091	0.356048370388974	-4.74765522525547	2.0578844230214e-06	0.0017564043550494
**NAV2**	2256	2449	2583	6156	4441	6119	2162	2560	2582	5964	4160	6405	3972	1.17812658753638	0.247883086905478	4.75275099339723	2.0066742519913e-06	0.0017564043550494
**MAGI3**	703	381	531	195	198	233	674	398	531	189	185	244	370	-1.3752062039391	0.299668779023636	-4.5890873531094	4.45188115715531e-06	0.00359969737986195
**CSRP2**	1434	1288	1156	562	811	547	1374	1347	1156	544	760	573	959	-1.04621097025734	0.231024612626822	-4.52856930853207	5.93844029176066e-06	0.00456161291011595
**SPTSSA**	1712	1508	1515	934	921	614	1640	1577	1515	905	863	643	1190	-0.972841246004376	0.21851419442037	-4.45207346179469	8.50450584769015e-06	0.00622165349228875
**TP53INP2**	3069	2303	3316	1338	1343	1585	2941	2408	3315	1296	1258	1659	2146	-1.04018554852234	0.23575459811763	-4.41215381090188	1.02347361159768e-05	0.00708480263265287
**CDK17**	2607	2059	2285	1412	1553	1196	2498	2153	2284	1368	1455	1252	1835	-0.767185425822611	0.174185310592709	-4.40442091937645	1.06066823244819e-05	0.00708480263265287
**COL3A1**	54975	179834	104753	17852	6843	29269	52675	188013	104729	17294	6410	30636	66626	-2.66823485193119	0.613017522520115	-4.35262411580354	1.34517671777673e-05	0.00826637996608155
**DDX58**	13917	19970	10278	5451	6941	4460	13335	20878	10276	5281	6502	4668	10157	-1.43521138368789	0.329297671879822	-4.35840124679553	1.31016016327752e-05	0.00826637996608155
**SOX9**	1431	1079	1206	344	658	242	1371	1128	1206	333	616	253	818	-1.6234897961688	0.374896423823569	-4.32745920345289	1.50839272685071e-05	0.0087595554355266
**P4HA2**	10068	6900	9724	5248	4091	4205	9647	7214	9722	5084	3832	4401	6650	-0.9971407528	0.231090438946586	-4.31493729	1.59648214668193e-05	0.0087595554355266

														75658		217475		
**ERF**	249	232	257	425	471	467	239	243	257	412	441	489	347	0.862032765234222	0.199766945653545	4.31519219760432	1.59464101649204e-05	0.0087595554355266
**GATA6**	1476	2227	1440	557	902	608	1414	2328	1440	540	845	636	1201	-1.35830088384287	0.318830239037583	-4.26026366866274	2.0418588349217e-05	0.0108169232003111
**BCL2L14**	39	44	52	13	9	6	37	46	52	13	8	6	27	-2.30470495224927	0.546791871721129	-4.21495832590704	2.49824316452529e-05	0.012793503245534
**TMEM2**	22412	12001	15554	8976	6938	5524	21474	12547	15550	8695	6499	5782	11758	-1.24070763195324	0.300170800025768	-4.13333885856564	3.57530995069992e-05	0.0163635016940976
**CPNE8**	1618	943	1164	677	600	462	1550	986	1164	656	562	484	900	-1.12066399648317	0.271321337080152	-4.13039390319721	3.62142197226659e-05	0.0163635016940976
**TRIM5**	2273	2759	2533	1376	1605	1391	2178	2884	2532	1333	1504	1456	1981	-0.823102882409218	0.198586413511697	-4.14480964661127	3.40096107016487e-05	0.0163635016940976
**DCAF5**	3957	4230	3776	2651	2613	2330	3791	4422	3775	2568	2448	2439	3241	-0.685410369811941	0.165681241808699	-4.13692197335977	3.51995736229269e-05	0.0163635016940976
**SPSB2**	31	50	37	97	135	122	30	52	37	94	126	128	78	1.55155520600643	0.379359085201908	4.08993817870643	4.31488182199377e-05	0.0189398655517972
**SEC24D**	10776	9994	9666	7164	6909	6527	10325	10449	9664	6940	6472	6832	8447	-0.588344094607459	0.143044844503307	-4.07709177493329	4.56024967423079e-05	0.0194608654847799
**ISG20**	671	456	473	213	300	258	643	477	473	206	281	270	392	-1.07246808399668	0.264805301626526	-4.05002497083406	5.12121679057541e-05	0.0212641225820568
**BRMS1L**	588	491	459	296	317	284	563	513	459	287	297	297	403	-0.80182007270095	0.198515693301245	-4.03907650507105	5.36620615921044e-05	0.0215593717796785
**CCDC109B**	1561	1297	1489	1000	935	792	1496	1356	1489	969	876	829	1169	-0.698948353224455	0.173333058314073	-4.03240074353265	5.52099357478056e-05	0.0215593717796785
**KLF10**	2160	2592	2011	4549	4099	3830	2070	2710	2011	4407	3840	4009	3174	0.852030511582296	0.211500547623872	4.02850262637395	5.61332338206821e-05	0.0215593717796785
**PLEKHA4**	2652	3149	2140	1281	1269	1440	2541	3292	2140	1241	1189	1507	1985	-1.01804370153983	0.253142307891541	-4.02162605697666	5.77977629746973e-05	0.0216572444670799

**LEPRE1**	3903	4394	4025	2591	2507	2543	3740	4594	4024	2510	2349	2662	3313	-0.716541877449495	0.179275093377103	-3.99688469798903	6.41815424416601e-05	0.0234766913459815
**SLC30A7**	5720	4770	4809	3362	3564	2645	5481	4987	4808	3257	3339	2769	4107	-0.705938250382288	0.178110779795744	-3.96347852270285	7.38655117155507e-05	0.026390601313628
**LRRC8C**	4066	1808	2087	1212	1015	673	3896	1890	2087	1174	951	704	1784	-1.47631095076637	0.373535630107796	-3.95226273418772	7.7415673967907e-05	0.0266798724984064
**ZSWIM6**	3542	1794	2967	1386	1305	1179	3394	1876	2966	1343	1223	1234	2006	-1.116242597406	0.282915630139281	-3.94549639005972	7.96348318642068e-05	0.0266798724984064
**XPO1**	4579	4510	4754	7319	7010	6936	4387	4715	4753	7090	6567	7260	5795	0.594229716831929	0.150638322380292	3.94474465356681	7.98850572757075e-05	0.0266798724984064
**BLZF1**	3338	3321	2678	1972	1951	1453	3198	3472	2677	1910	1828	1521	2434	-0.829737762620723	0.210779385206512	-3.93652235871066	8.26709210749796e-05	0.027022837456913
**DUSP6**	8813	9028	5663	2352	4504	1830	8444	9439	5662	2278	4219	1915	5326	-1.48458383868178	0.382268221542634	-3.88361824242354	0.000102913448771942	0.0322665166017009
**SLC20A1**	10622	8138	8716	5719	4784	3054	10178	8508	8714	5540	4482	3197	6770	-1.05156448416454	0.270707375154852	-3.88450622582047	0.000102538065442567	0.0322665166017009
**LMO2**	249	227	370	104	53	116	239	237	370	101	50	121	186	-1.63936201746043	0.423288178169845	-3.87292181073538	0.00010753833541439	0.0326799709418132
**MMP19**	1297	1173	1057	787	605	625	1243	1226	1057	762	567	654	918	-0.830111903516989	0.214455834236936	-3.87078256215618	0.000108486527242887	0.0326799709418132
**RASL11A**	164	194	182	83	105	66	157	203	182	80	98	69	132	-1.12641802194998	0.292826538527535	-3.84670743169019	0.000119715780186172	0.0353691063653877
**C4ORF32**	1202	1853	1129	705	701	566	1152	1937	1129	683	657	592	1025	-1.12608356163995	0.29547797128152	-3.81105757818764	0.000138373529491587	0.0366522850617113
**SPRED1**	10820	10203	7873	5247	5504	2918	10367	10667	7871	5083	5156	3054	7033	-1.12058319392872	0.29356305813749	-3.81718054389492	0.000134985394720745	0.0366522850617113
**ELOVL6**	2286	1963	2009	900	1470	878	2190	2052	2009	872	1377	919	1570	-0.980394814342323	0.256964099022563	-3.81529878326014	0.000136018254241204	0.0366522850617113

**SCYL2**	4009	3196	3696	2560	2215	2167	3841	3341	3695	2480	2075	2268	2950	-0.672924319320111	0.175560638946874	-3.83300222280315	0.000126588809220043	0.0366522850617113
**GBP5**	4879	6668	5706	9477	15260	11885	4675	6971	5705	9181	14295	12440	8878	1.04966789503898	0.27534590438483	3.81217907484087	0.000137747018153568	0.0366522850617113
**BRICD5**	36	28	28	77	78	107	34	29	28	75	73	112	59	1.49818798283006	0.392021855775751	3.82169504265362	0.000132537527555241	0.0366522850617113
**SMAP2**	1660	1642	2088	1096	761	943	1591	1717	2088	1062	713	987	1359	-0.966132199055545	0.253825815150006	-3.80628380043099	0.000141070498397741	0.0367333231674477
**TPST1**	2634	2085	2125	1363	1594	1062	2524	2180	2125	1320	1493	1112	1792	-0.798563898725708	0.21027119287518	-3.79778079824633	0.000145997344057355	0.0373826199458857
**RCOR3**	331	294	356	664	528	694	317	307	356	643	495	726	474	0.92671339398267	0.244349756726658	3.79256933338935	0.000149096603466757	0.037550346214095
**SLC37A3**	6583	5867	5254	3901	4196	3348	6308	6134	5253	3779	3931	3504	4818	-0.657911053100257	0.173669912519833	-3.78828458858877	0.000151691033553009	0.0375875013669166
**MIDN**	670	537	912	1199	2090	1689	642	561	912	1162	1958	1768	1167	1.20820885706641	0.31956040054821	3.78084660988566	0.000156295953943922	0.0381138847688963
**RP11-39K24.14**	42	43	30	12	8	5	40	45	30	12	7	5	23	-2.23668354823066	0.592271633613798	-3.77644888137447	0.000159080189622913	0.0381867023933878
**NCR3LG1**	659	453	580	358	272	269	631	474	580	347	255	282	428	-0.932152617902822	0.248485896692091	-3.7513300767242	0.000175898947341786	0.0415743927386439
**RP11-443P15.2**	162	267	172	424	475	520	155	279	172	411	445	544	334	1.20791425341919	0.323238794603525	3.73690990557238	0.000186295637110094	0.0420891157782702
**IDO2**	0	5	3	25	39	25	0	5	3	24	37	26	16	3.42122639925899	0.91395618410448	3.74331555359101	0.000181607925089245	0.0420891157782702
**SPOCK2**	2	6	3	13	38	68	2	6	3	13	36	71	22	3.42287774091106	0.915629955889689	3.73827627514126	0.000185286272379036	0.0420891157782702
**ATF3**	7702	5945	6508	4585	4265	4278	7380	6215	6507	4442	3995	4478	5503	-0.638327324721081	0.171125104219717	-3.73017931899401	0.000191343540376377	0.0426030552290186
**MGP**	33	264	43	10	17	12	32	276	43	10	16	13	65	-3.19787566896708	0.859817391801997	-3.71924980752599	0.000199815359987708	0.0438537625070165
